# FIRM: Flexible integration of single-cell RNA-sequencing data for large-scale multi-tissue cell atlas datasets

**DOI:** 10.1093/bib/bbac167

**Published:** 2022-05-14

**Authors:** Jingsi Ming, Zhixiang Lin, Jia Zhao, Xiang Wan, The Tabula Microcebus Consortium, T T M Consortium, C Ezran, S Liu, Can Yang, Angela Ruohao Wu

**Affiliations:** 1 Academy of Statistics and Interdisciplinary Sciences, KLATASDS-MOE, East China Normal University, Shanghai, China; 2 Department of Mathematics, The Hong Kong University of Science and Technology, Hong Kong SAR, China; 3 Department of Statistics, The Chinese University of Hong Kong, Hong Kong SAR, China; 4 Shenzhen Research Institute of Big Data, Shenzhen, China; 5 Pazhou Lab, Guangzhou, China; 6 Division of Life Science, The Hong Kong University of Science and Technology, Hong Kong SAR, China; 7 Department of Chemical and Biological Engineering, The Hong Kong University of Science and Technology, Hong Kong SAR, China

**Keywords:** single-cell RNA sequencing, data integration, cell atlas, bioinformatics

## Abstract

Single-cell RNA-sequencing (scRNA-seq) is being used extensively to measure the mRNA expression of individual cells from deconstructed tissues, organs and even entire organisms to generate cell atlas references, leading to discoveries of novel cell types and deeper insight into biological trajectories. These massive datasets are usually collected from many samples using different scRNA-seq technology platforms, including the popular SMART-Seq2 (SS2) and 10X platforms. Inherent heterogeneities between platforms, tissues and other batch effects make scRNA-seq data difficult to compare and integrate, especially in large-scale cell atlas efforts; yet, accurate integration is essential for gaining deeper insights into cell biology. We present FIRM, a re-scaling algorithm which accounts for the effects of cell type compositions, and achieve accurate integration of scRNA-seq datasets across multiple tissue types, platforms and experimental batches. Compared with existing state-of-the-art integration methods, FIRM provides accurate mixing of shared cell type identities and superior preservation of original structure without overcorrection, generating robust integrated datasets for downstream exploration and analysis. FIRM is also a facile way to transfer cell type labels and annotations from one dataset to another, making it a reliable and versatile tool for scRNA-seq analysis, especially for cell atlas data integration.

## Introduction

The advent of single-cell RNA-sequencing (scRNA-seq) technology has enabled discovery of new cell types [1], understanding of dynamic biological processes [2, 3] and spatial reconstruction of tissues [4]. Ongoing advancement in scRNA-seq technology has led to vast improvements in the scale and cost of the experiments [5–8], providing unprecedented opportunities for biological insight. Prominent examples include recent efforts to generate cell atlases for whole organisms, including human [9–11], mouse [12–15] and mouse lemur [16]. These projects have generated scRNA-seq datasets encompassing a comprehensive set of tissues from the organism of interest, and to ensure both technical sensitivity and scale in cell numbers profiled, many of these atlases employ multiple different single-cell profiling technology platforms, including SMART-seq2 (SS2) and 10X Chromium (10X). Integrating datasets from different tissue types, samples and experiments, and from different platforms, not only enables the transfer of cell-type labels and annotations from one dataset to another but also makes the atlases more comprehensive and cohesive, which benefits downstream biological analyses. However, complex technical variations and heterogeneities that exist between datasets make integration challenging.

Existing methods have been designed for the integration of scRNA-seq datasets across different samples, experiments, species or types of measurement, but they do not account for the integration of datasets across multiple platforms. Specifically, SS2 and 10X are two frequently used scRNA-seq platforms with their unique strengths and weaknesses. SS2 is a plate-based full-length approach with high transcriptome coverage per cell and greater sensitivity [17], whereas the microfluidic droplet-based method, 10X, generally has lower coverage per cell and a higher dropout rate [18]. But 10X is able to profile hundreds of thousands of cells per study with low per cell costs [8], which enables more reliable detection of rare cell types, and the inclusion of unique molecular identifiers (UMIs) in 10X allows the removal of amplification bias and in turn enables more accurate transcript abundance quantification [19]. Harmonizing datasets across multiple platforms for integrative analysis can take advantage of the strengths of each technology and achieve higher accuracy, better comparison across datasets and studies, and higher statistical power for downstream analysis. Furthermore, integration enables use of 10X for discovering new cell types, while taking advantage of the depth and sensitivity of SS2 to investigate details such as transcript isoforms, splicing [20–22] and allelic expression [21, 23]. This is particularly important for large-scale cell atlas projects, which are intended to serve as robust and comprehensive reference datasets for future mining. Due to technical variations and characteristic differences in SS2 and 10X datasets, not accounting for platform-specific characteristics during integration can lead to inaccuracies under different scenarios: sometimes resulting in poor alignment of cells from the same cell type; other times mixing cells from different cell types inappropriately, giving rise to overcorrection. An ideal method requires identification of the main technical variation for integration and designing a specific approach to address it.

Through comprehensive data exploration, we found that the differences in depth of expression profiles are the main technical variation between SS2 and 10X datasets and the heterogeneity in cell type composition accounts for the main problem preventing accurate integration. Datasets with different cell type compositions have different directions of maximum variance chosen by principal component analysis (PCA) and perform differently after standard preprocessing procedures including normalization and scaling. We have developed a flexible algorithm, FIRM, to specifically account for this composition effect, thereby harmonizing datasets across multiple tissue types, platforms and experimental batches. Authors of other methods such as Mutual Nearest Neighbor (MNN) [24] and Scanorama [25] have also observed the influence of cell type composition on integration and tried to reduce this effect by modifying the underlying expression data to align cells with high similarity. However, using this approach, overcorrection can occur, where close but not identical cell types may be merged into the same cluster inappropriately, and this is especially common in atlas projects when there are often dataset-specific cell types. In contrast, FIRM applies a re-scaling procedure based on subsampling for both datasets in a unified workflow. Overcorrection can be avoided with this approach and the original structure for each dataset can be largely preserved, generating a reliable input for downstream analysis. We applied FIRM to integrate numerous scRNA-seq datasets generated using different platforms and sample types. Compared with existing state-of-the-art methods, FIRM not only demonstrates superior integration accuracy but also effectively avoids overcorrection in all tested datasets.

## Materials and methods

### Datasets

We adopted scRNA-seq datasets from three cell atlas projects as the benchmark datasets in this study. We used 44 779 cells profiled using SS2 from 20 organs and 54 865 cells profiled using 10X from 12 mouse organs in Tabula Muris [12], 12 329 cells profiled using SS2 from 20 organs and tissues in 3 individuals and 231 752 cells profiled using 10X from 25 organs and tissues in 4 individuals from Tabula Microcebus [16], and 3987 cells profiled using SS2 and 9744 cells profiled using 10X for Patient 1 from Human Lung Atlas [26]. For specific information of the datasets, please see the ‘Data availability’ section.

### Key problem

**Figure 1:**
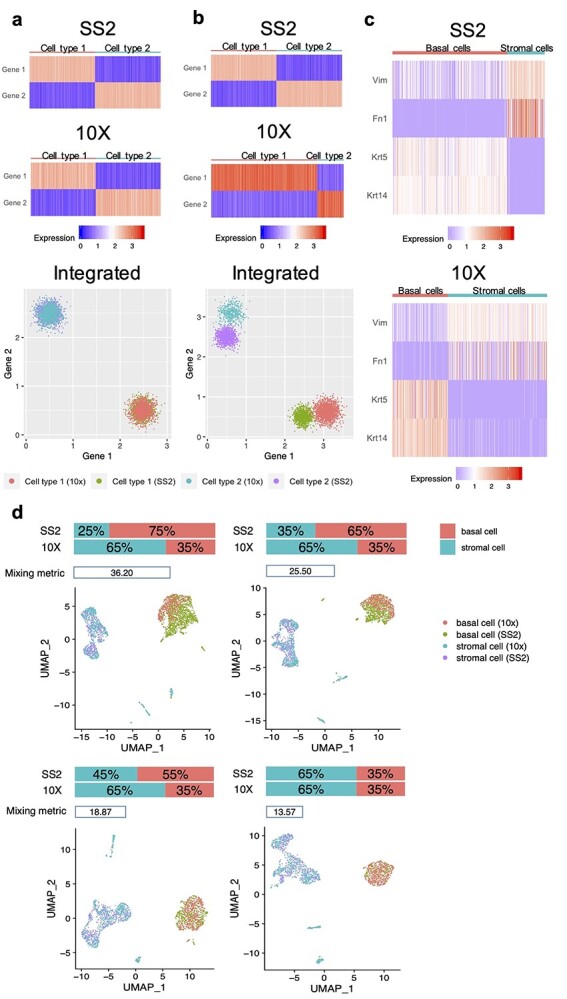
Illustration of the influence of cell type composition for scRNA-seq datasets integration based on hypothetical datasets (**A** and **B**) and real datasets (**C** and **D**). A and B, Gene expressions for cells in SS2 dataset, 10X dataset and integrated dataset after scaling to unit variance for each gene. Each row represents one gene and each column represents one cell. The color gradient shows the gene expression levels in the cells. (A) In the first scenario, the cell type compositions in the hypothetical datasets are the same across datasets (SS2: 50% cell type 1 and 50% cell type 2; 10X: 50% cell type 1 and 50% cell type 2). (**B**) In the second scenario, the cell type compositions are different across datasets (SS2: 50% cell type 1 and 50% cell type 2; 10X: 80% cell type 1 and 20% cell type 2). (**C** and **D**) Illustration of the key problem for integration based on the mammary gland scRNA-seq datasets generated by SS2 and 10X from Tabula Muris, withholding only the basal cells and stromal cells. (**C**) Marker expressions for basal cells and stromal cells in SS2 dataset and 10X dataset after scaling to unit variance for each gene, where the cell type compositions are different across datasets (SS2: 75% basal cells and 25% stromal cells; 10X: 35% basal cells and 50% stromal cells). (**D**) Uniform manifold approximation and projection (UMAP) visualization and mixing metric for the integrated dataset with different cell type composition by subsampling basal cells in SS2 dataset.

We found that differences in cell type composition is a major factor preventing accurate integration of scRNA-seq data generated by different technology platforms. To specifically investigate the influence of cell type composition on integration outcomes, we consider a toy example with two scenarios using hypothetical datasets in which cells from the same type have similar expression patterns across different platforms. In the first scenario, the cell type proportions are consistent across different platforms (SS2: cell type 1/cell type 2 = 50%/50%, 10x: cell type 1/cell type 2 = 50%/50%); in the second scenario, the cell type proportions are different (SS2: cell type 1/cell type 2 = 50%/50%, 10x: cell type 1/cell type 2 = 80%/20%). We scaled the expression value for each gene to unit variance for each dataset, which is the standard preprocessing procedure applied to prevent the dominance of highly expressed genes and is also necessary to reduce the difference in sequencing depth for dataset integration across platforms. In the first scenario, cells belonging to the same cell type have similar gene expression levels after scaling and are well mixed across platforms (Figure 1A). However, in the second scenario, the scaled expression values in SS2 and 10X datasets for cells of the same type show large differences, resulting in poor integration of these two datasets (Figure 1B). This demonstrates that when cell-type composition is skewed between the two datasets being integrated, it impacts the integration outcome and can result in inaccurate cell merging.

To verify our hypothesis using real scRNA-seq datasets, we extracted the basal cells and stromal cells from the Tabula Muris [12] mouse mammary gland scRNA-seq data that was generated using SS2 and 10X, in which their relative proportions across platforms are vastly different (SS2: basal cells/stromal cells = 75%/25%; 10X: basal cells/stromal cells = 35%/65%). After data preprocessing, the expression levels for the same cell type marker (stromal cells: *Vim* and *Fn1*; basal cells: *Krt5* and *Krt14*) across platforms are different in expression modes and dispersions (Figure 1C). We then integrated the dataset by concatenating the scaled SS2 and 10X expression data matrices, and in visualizing the outcome we found that basal cells across platforms did not correctly merge into one single cluster (Figure 1D, left top panel). In order to confirm whether this poor alignment is caused by the difference in cell type proportions, we performed subsampling to gradually reduce the proportion of basal cells in SS2 dataset from 75 to 35%, to match that of the 10X dataset. Then, we integrated the 10X dataset with these subsets of SS2 dataset, and evaluated the performance. In addition to the UMAP [27] plot for visualization (see discussions of the variability of UMAP in Supplementary Document and Supplementary Figures 1 and 2, see Supplementary Data available online at https://academic.oup.com/bib), we also calculated the mixing metric (Supplementary Document) to measure how well the datasets mixed after integration, where a lower score typically indicates better mixing performance. We indeed observed that more consistent cell type proportions gave rise to better alignments (Figure 1D). Therefore, we concluded that the effects of heterogeneity in cell type composition between SS2 and 10X datasets account for one of the main technical variation preventing accurate integration of scRNA-seq data across platforms.

### Overview of FIRM method

FIRM harmonizes datasets while accounting for the difference in cell type composition. Here we use the integration of one SS2 and one 10X dataset to illustrate the alignment workflow of FIRM. FIRM takes two scRNA-seq expression matrices as the input, and performs the following steps (see Supplementary Figure 3, see Supplementary Data available online at https://academic.oup.com/bib, for a graphical illustration): (i) for each dataset, we conduct pre-processing procedure which includes normalization, scaling and feature selection; (ii) then, we perform dimension reduction for each dataset using PCA and cluster cells based on the obtained low-dimensional representations; (iii) in order to align clusters in 10X dataset with clusters in SS2 dataset representing the same cell types, we check the alignment via subsampling to avoid overcorrection; (iv) for each pair of aligned clusters, we subsample the cells within the cluster to ensure that cell-type proportions are the same in SS2 and 10X datasets, and then based on these subsampled cells, we calculate the standard deviation to perform re-scaling on each of the full datasets; (v) finally, we merge the scaled data to obtain the integrated dataset. In our cluster alignment procedure, we used the clusters in the 10X dataset as anchors to query clusters in the SS2 dataset. For more general integration scenarios with two datasets, we treat the one with more cells as the anchor dataset. More technical details are presented in the following sections.

### Data preprocessing

For all scRNA-seq datasets, we performed normalization, scaling and feature selection. More specifically, for each dataset, we used the gene expression matrix $\boldsymbol{X}$, where ${X}_{ij}$ is the number of reads (for SS2) or unique molecular identified (UMI, for 10X) for gene *i* that are detected in cell *j*, and employed the log-normalization which is the default normalization method in Seurat [28]. Then, we scaled the expression values for each gene across all cells in each dataset so that each gene has unit variance. For each dataset, we implemented the ‘FindVariableFeatures’ function in Seurat to select top 4000 highly variable genes. For integrative analysis of two datasets across platforms, we selected genes that are highly variable in both datasets.

### Cell clustering for each dataset

We first performed PCA for each dataset, where the scaled data with the highly variable genes is used. The number of PCs is a hyperparameter (see ‘Hyperparameters in the algorithm’ for more details). Then for each dataset, we clustered cells based on their PC scores using the clustering approach in Seurat by the ‘FindClusters’ function. The resolution parameter which is used to control the number of clusters is tuned in FIRM for better integration (see ‘Resolution in clustering’ step).

### Cluster alignment via subsampling

Next, the cell clusters that represent the same cell types across the two datasets need to be aligned. The alignment was checked via subsampling to avoid overcorrection. First, we concatenated the scaled SS2 and 10X data and performed PCA on the combined data to obtain the low-dimensional representations for each cell. Next, we attempt to align each 10X cluster with an SS2 cluster in the following steps.

(1) For 10X cluster *a*, we considered SS2 cluster *b*, which is among the five nearest SS2 clusters to 10X cluster *a*. We calculated the distance between their centers: $\parallel{\boldsymbol{Z}}_{a\cdot }-{\boldsymbol{Z}}_{b\cdot }{\parallel}^2$, where ${\boldsymbol{Z}}_{a\cdot }=\frac{1}{n_a}\sum_{i=1}^{n_a}{\boldsymbol{Z}}_{a,i}$, ${\boldsymbol{Z}}_{b\cdot }=\frac{1}{n_b}\sum_{i^{\prime }=1}^{n_b}{\boldsymbol{Z}}_{b,{i}^{\prime }}$ are the centers of 10X cluster *a* and SS2 cluster *b*, ${\boldsymbol{Z}}_{a,i}$, ${\boldsymbol{Z}}_{b,{i}^{\prime }}$ are the low-dimensional representations for cell $i$ from 10X cluster *a* and cell ${i}^{\prime }$ from SS2 cluster *b*, and ${n}_a$, ${n}_b$ are the numbers of cells in 10X cluster *a* and SS2 cluster *b*, respectively.

(2) We then calculated the 75% quantile among all distances from the cells in SS2 cluster *b* to their cluster center: ${Q}_{0.75}\Big(\parallel{\boldsymbol{Z}}_{b,1}-{\boldsymbol{Z}}_{b\cdot }{\parallel}^2,\parallel{\boldsymbol{Z}}_{b,2}-{\boldsymbol{Z}}_{b\cdot }{\parallel}^2,\dots, \parallel{\boldsymbol{Z}}_{b,{n}_b}-{\boldsymbol{Z}}_{b\cdot }{\parallel}^2\Big)$. We check if the following criterion holds:

$$\begin{eqnarray*} &&\hskip-12pt\parallel{\boldsymbol{Z}}_{a\cdot }-{\boldsymbol{Z}}_{b\cdot }{\parallel}^2\nonumber\\ &&\hskip-9pt<{Q}_{0.75}\left(\parallel{\boldsymbol{Z}}_{b,1}-{\boldsymbol{Z}}_{b\cdot }{\parallel}^2,\parallel{\boldsymbol{Z}}_{b,2}-{\boldsymbol{Z}}_{b\cdot }{\parallel}^2,\dots, \parallel{\boldsymbol{Z}}_{b,{n}_b}-{\boldsymbol{Z}}_{b\cdot }{\parallel}^2\right). \end{eqnarray*}$$

(3) We considered the nearest SS2 cluster, among the five nearest SS2 clusters to 10X cluster *a*, that also satisfied the criterion in step (2) to be aligned with 10X cluster *a*.

However, because of the difference in its abundances in 10X and SS2 data, even the same cell type may not be aligned after steps (1–3) described above. To address this issue, for the 10X clusters which had not been aligned, we further performed subsampling to adjust the proportions of the 10X and SS2 clusters being considered and checked the alignment. For example, when we consider the 10X cluster *a* and the SS2 cluster *b*, if the proportion of 10X cluster *a* in 10X dataset is larger than the proportion of SS2 cluster *b* in SS2 dataset, i.e. $\frac{\#\mathrm{of}\ \mathrm{cells}\ \mathrm{in}\ 10\mathrm{X}\ \mathrm{cluster}\ a}{\#\mathrm{of}\ \mathrm{cells}\ \mathrm{in}\ 10\mathrm{X}\ \mathrm{dataset}}>\frac{\#\mathrm{of}\ \mathrm{cells}\ \mathrm{in}\ \mathrm{SS}2\ \mathrm{cluster}\ b}{\#\mathrm{of}\ \mathrm{cells}\ \mathrm{in}\ \mathrm{SS}2\ \mathrm{dataset}}$, we subsampled the cells in 10X cluster *a* to obtain a subset, so that the proportion of 10X cluster *a* in this subset was the same with the proportion of SS2 cluster *b* in SS2 dataset, i.e. $\frac{\#\mathrm{of}\ \mathrm{cells}\ \mathrm{in}\ 10\mathrm{X}\ \mathrm{cluster}\ a\ \mathrm{in}\ \mathrm{the}\ \mathrm{subset}}{\#\mathrm{of}\ \mathrm{cells}\ \mathrm{in}\ 10\mathrm{X}\ \mathrm{subset}}=\frac{\#\mathrm{of}\ \mathrm{cells}\ \mathrm{in}\ \mathrm{SS}2\ \mathrm{cluster}\ b}{\#\mathrm{of}\ \mathrm{cells}\ \mathrm{in}\ \mathrm{SS}2\ \mathrm{dataset}}$. We then calculated the standard deviation of each gene across cells in this subset based on the original scaled expression values, i.e. ${s}_j= sd({Y}_{ij}),i\in 10\mathrm{X}\ \mathrm{subset},$ where ${Y}_{ij}$ is the scaled data after the preprocessing procedure described in the ‘Data preprocessing’ step. We performed re-scaling for cells in the whole 10X dataset using this standard deviation, i.e. $\frac{Y_{ij}}{s_j},i\in 10\mathrm{X}\ \mathrm{dataset}$. Based on the re-scaled data, we checked the alignment again using steps (1–3) described above. If one SS2 cluster is aligned with more than one 10X clusters, we merged the 10X clusters which are aligned with the same SS2 cluster and then considered them as a whole.

### Re-scaling via subsampling and generation of integrated data

To calculate the scaling factor for effective re-scaling, we performed subsampling for cells in the aligned SS2 and 10X clusters to obtain the SS2 subset and the 10X subset which contain the same types of cells and have the same cell-type proportions as well. Based on each of the subsampled datasets, we computed the standard deviations for each gene across cells on the original scaled expression values, i.e. ${s}_{SS2\ j}= sd({Y}_{ij}),i\in \mathrm{SS}2\ \mathrm{subset},$ and ${s}_{10X\ j}= sd({Y}_{ij}),i\in 10\mathrm{X}\ \mathrm{subset}$, where ${Y}_{ij}$ is the scaled data after the preprocessing procedure described in the ‘Data preprocessing’ step. We used the calculated standard deviations to re-scale the gene expression values for cells in the whole SS2 and 10X datasets. i.e. $\frac{Y_{ij}}{s_{SS2\ j}},i\in \mathrm{SS}2\ \mathrm{dataset}$ and $\frac{Y_{ij}}{s_{10X\ j}},i\in 10\mathrm{X}\ \mathrm{dataset}$. We concatenated the re-scaled data directly to obtain the integrated data.

### Resolution in clustering

Cluster alignment is the key for effective integration. To obtain the best pair of resolution parameters for clustering, the default option is to search through pairs in the range of [0.1, 2] × [0.1, 2]. Users can also set other customized ranges. For each pair of resolution parameters, we aligned clusters between datasets and generated the integrated data following the (iii–v) steps described in the ‘Overview of FIRM method’ section. Based on the integrated datasets generated using every pair of resolution parameter, we calculated the corresponding mixing metric. As our method does not suffer from overcorrection, smaller mixing metric indicates better integration. Therefore, we chose the pair of resolution parameters that yields the smallest mixing metric and output the corresponding integrated data. This procedure is fully automatic and naturally allows parallelization.

### Hyperparameters in the algorithm

The number of PCs is the only hyperparameter that needs to be specified in the FIRM algorithm. The number of PCs is chosen according to its relationship with the variance explained, and needs to be the same for datasets in integrative analysis. For the scRNA-seq datasets analyzed in this paper, we chose the number of PCs as the larger number in the original analyses that were performed separately on SS2 and 10X datasets [12, 16, 26]. We found that the performance of FIRM is insensitive to the number of PCs (Supplementary Figures 4 and 5, see Supplementary Data available online at https://academic.oup.com/bib).

The resolution parameters for clustering are tuned automatically in the FIRM algorithm as described in the ‘Resolution in clustering’ step. Other parameters in the algorithm are all fixed. Other parameters in the algorithm are all fixed. For example, the number of nearest neighbors in the clustering method was set as 20, which is the default value in the ‘FindNeighbors’ function in Seurat. FIRM is also insensitive to this parameter (Supplementary Figures 6 and 7, see Supplementary Data available online at https://academic.oup.com/bib).

### Baseline model

We considered the special case without the re-scaling procedure to be the baseline model. We directly concatenated the scaled expression matrix for the overlapped highly variable genes after data processing to obtain the integrated dataset. If the mixing metric of the integrated dataset after re-scaling does not decrease, we chose the baseline model.

### Label transfer and match scores

The integration of datasets enables efficient label transfer between datasets. Suppose we want to use the annotations for cells in the 10X dataset to annotate cells in the SS2 dataset. For each SS2 cell, we found its 10 nearest 10X cells in the integrated dataset and summarized the cell types they belong to. We chose the cell type with the highest frequency to annotate the SS2 cell.

In case that some cell types do not exist in the 10X dataset, we defined the match score to measure whether the cell in SS2 is present in 10X data. For each SS2 cell, we divided its averaged distance to its 10 nearest neighbors in the SS2 dataset by that in the 10X dataset. Lower score means less likely to be present in 10X data.

### Integration of multiple datasets

When we have more than two datasets, e.g. three datasets to be integrated, our strategy is to first integrate two of them and then integrate the result with the third one. Since datasets are harmonized after integration, the integrated data can be considered as one dataset. Regarding the order of integration, we suggest integrating datasets with high similarity first. This is because it is likely that more clusters are aligned, which will lead to better calculation of the scaling factor and better utilization of the shared information across datasets. To measure the similarity between datasets, we compute the number of overlapped highly variable genes between two datasets and choose the pair with more overlapped highly variable genes to integrate first.

## Results

### FIRM provides accurate mixing of shared cell type identities and preserves local structure for each dataset

We examined the performance for the integration of SS2 and 10X scRNA-seq datasets generated from the same tissue type where most cell type identities are shared across platforms. We applied FIRM to numerous paired SS2 and 10X scRNA-seq datasets and compared with existing state-of-the-art methods, including Seurat [28], Harmony [29], BBKNN [30], BUSseq [31], LIGER [32], Scanorama [25], MNN [24], scVI [33] and ZINB-WaVE [34]. The datasets include 13 pairs of SS2 and 10X scRNA-seq datasets from Tabula Muris [12], 25 pairs from Tabula Microcebus [16] and one pair in Human Lung Cell Atlas [26]. The integration performance is evaluated by four metrics: mixing metric, local structure metric, average silhouette width (ASW) and adjusted rand index (ARI) (Supplementary Document).

**Figure 2:**
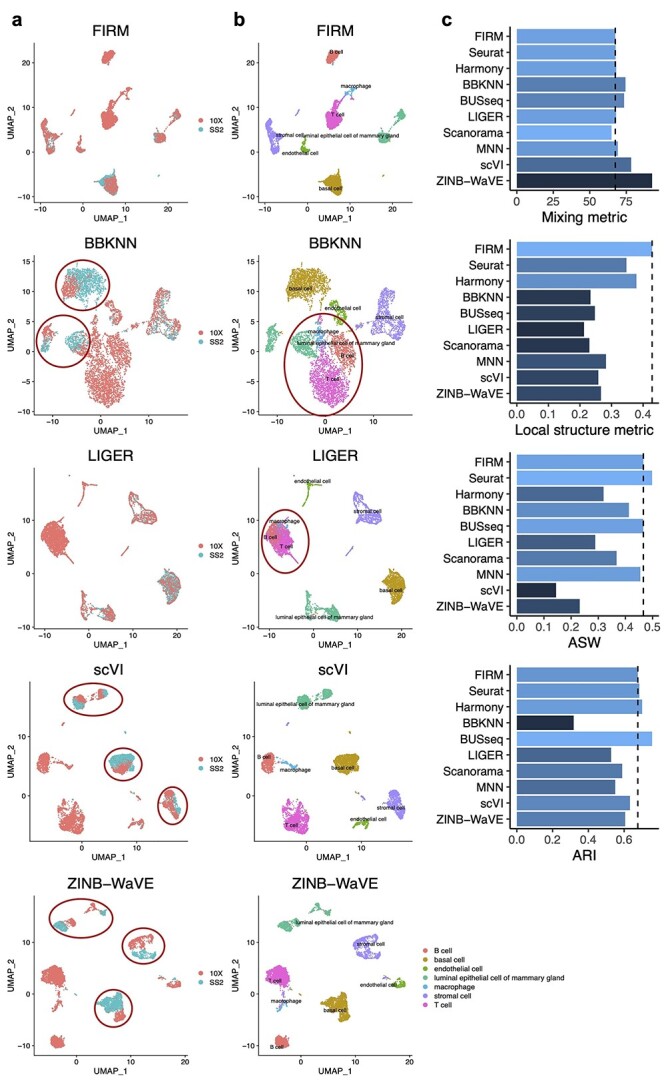
Comparison of integration methods based on the mammary gland scRNA-seq datasets generated by SS2 and 10X from Tabula Muris. (**A** and **B**) UMAP plots of the integrated scRNA-seq dataset colored by platform (**A**) and by cell type (**B**) using FIRM, Seurat, BBKNN, BUSseq, LIGER, scVI and ZINB-WaVE. The red circles highlight the problems of the integration results given by these methods. (**C**) Metrics for evaluating performance across the 10 methods on four properties: cell mixing across platforms (Mixing metric), the preservation of within-dataset local structure (Local structure metric), average silhouette width of annotated subpopulations (ASW) and adjusted rand index (ARI). The color (from light to dark) represents the performance (from the best to the worst). The dashed lines were set at the values for FIRM as reference lines.

For all datasets tested, FIRM outperforms or is comparable to all other bench-marked methods for integration of SS2 and 10X datasets with relatively low mixing metric, and high local structure metric, including ARI and ASW (Figure 2 and Supplementary Figures 8–38, see Supplementary Data available online at https://academic.oup.com/bib). FIRM not only provides accurate mixing of shared cell type identities but also achieves superior preservation of the local structure for each dataset, which is one of the greatest advantages of FIRM over other methods. This is because FIRM harmonizes datasets through a re-scaling procedure without smoothing the expression of similar cell types across datasets towards each other, so that the relative expression patterns across cells within each dataset can be largely preserved. For almost all (35 out of 39) the integrated datasets, FIRM achieved the highest local structure metric compared with all other methods (Supplementary Figure 38, see Supplementary Data available online at https://academic.oup.com/bib), indicating minimal distortion of the between-cell-type relationships within each dataset, thus providing more credible integrated data for downstream analysis. For other benchmarked methods, different situations arose indicating non-ideal integration, including poor mixing of shared cell types, inappropriate mixing of different cell types and weak preservation of the original dataset structure.

**Figure 3:**
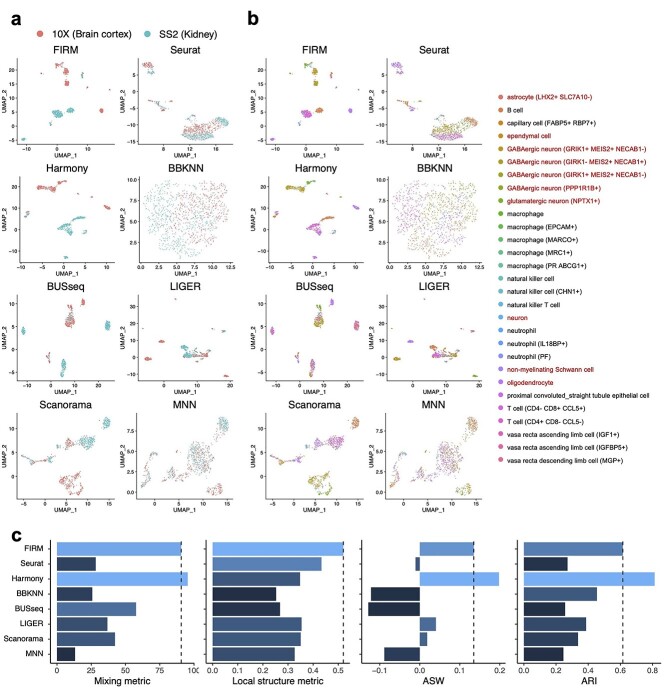
Comparison of integration methods for scRNA-seq datasets from two tissues in Tabula Microcebus (lemur 2) generated by different platforms (Kidney: SS2, Brain cortex: 10X). For clear illustration, we withheld several cell types in each of the dataset to make the cell types non-overlapped across datasets. (**A** and **B**) UMAP plots of scRNA-seq datasets colored by platform (**A**) and by cell type (**B**) after integration using FIRM, Seurat, Harmony, BBKNN, BUSseq, LIGER, Scanorama and MNN. The labels for cell types in Brain cortex (10X) are colored by red. (**C**) Metrics for evaluating performance across the eight methods on four properties: cell mixing across platforms (Mixing metric), the preservation of within-dataset local structure (Local structure metric), average silhouette width of annotated subpopulations (ASW) and adjusted rand index (ARI). The color (from light to dark) represents the performance (from the best to the worst). The dashed lines were set at the values for FIRM as reference lines.

**Figure 4:**
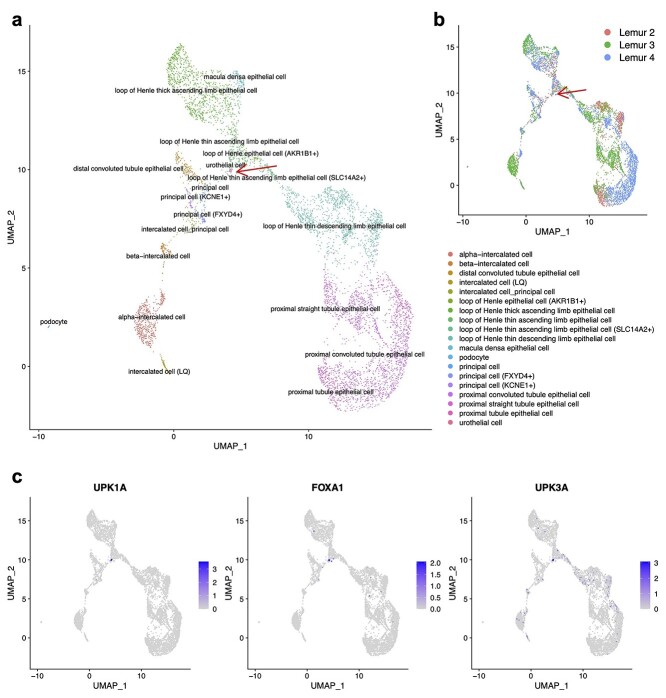
The FIRM integration for the kidney datasets across individuals and platforms in Tabula Microcebus. We subset the scRNA-seq datasets to keep the cells belonging to the epithelial compartment. (**A** and **B**) UMAP plots colored by cell type (**A**) and by individual (**B**) after integration using FIRM. (**C**) The expression levels of three marker genes (*UPK1A*, *FOXA1* and *UPK3A*) for urothelial cells.

**Figure 5:**
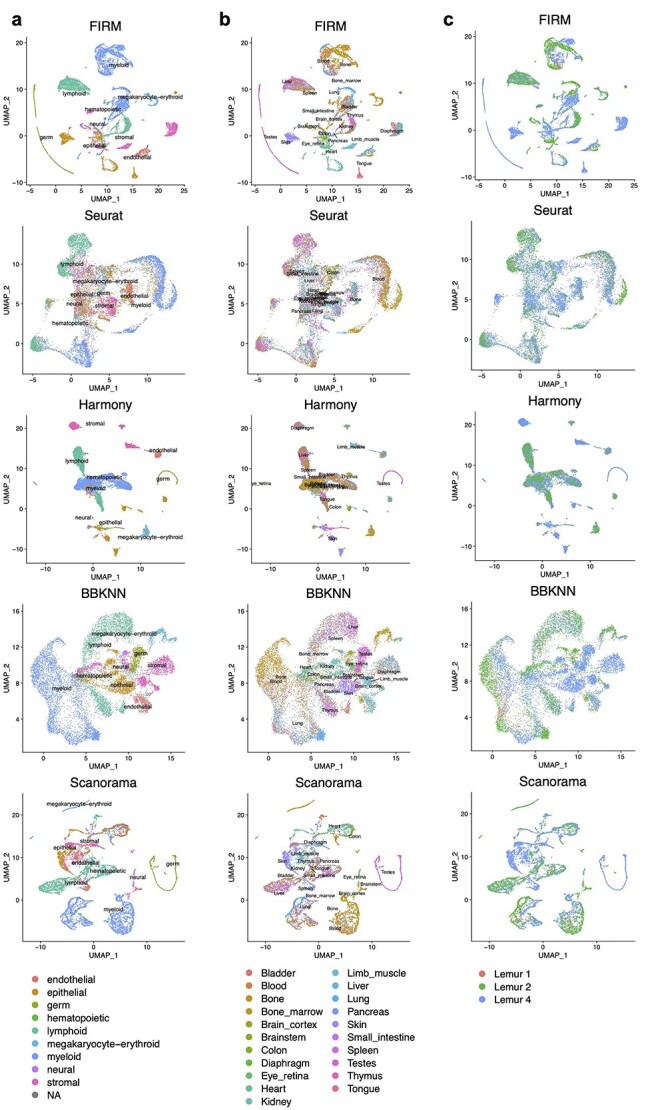
Comparison of FIRM, Seurat, Harmony, BBKNN and Scanorama for integration of all SS2 datasets across individuals and tissues in Tabula Microcebus. (**A**–**C**) UMAP plots of scRNA-seq datasets colored by compartment (**A**), by tissue (**B**) and by individual (**C**) after integration using FIRM, Seurat, Harmony, BBKNN and Scanorama.

**Figure 6:**
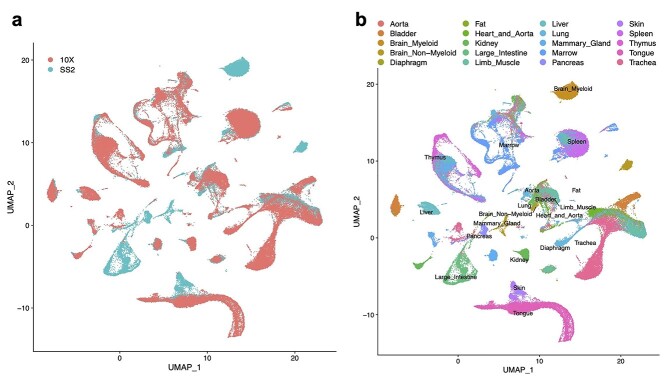
The performance of FIRM for integrating the whole SS2 dataset and 10X dataset of the entire organism in Tabula Muris. (**A** and **B**) UMAP plots of scRNA-seq datasets colored by platform (**A**) and by tissue (**B**) after integration using FIRM.

Specifically, scVI and ZINB-WaVE are the two methods with the highest mixing metric, and this inadequate mixing of cell types can be seen in UMAP plots even by visual inspection (Figure 2, Supplementary Figures 8–38 and Supplementary Table, see Supplementary Data available online at https://academic.oup.com/bib). BBKNN, BUSseq and Scanorama are also shown to have poor mixing performance in some cases (Supplementary Table; BBKNN: Figure 2, Supplementary Figures 9, 10, 20, 24 and 37; BUSseq: Supplementary Figures 9, 12, 23, 24, 28 and 37; Scanorama: Supplementary Figures 14, 24 and 34, see Supplementary Data available online at https://academic.oup.com/bib).

LIGER overcorrected the datasets for some cases resulting in inappropriate mixing of different cell types, which is reflected by low ARIs. For example, LIGER incorrectly merged the B cells, macrophages and T cells in the Tabula Muris mammary gland dataset (Figure 2; other examples are shown in Supplementary Table and Supplementary Figures 30, 33, 36 and 37, see Supplementary Data available online at https://academic.oup.com/bib). Harmony also has the phenomenon of overcorrection for some cases (Supplementary Table and Supplementary Figures 8, 20 and 34, see Supplementary Data available online at https://academic.oup.com/bib).

For the preservation of original structure for each dataset, BUSseq was shown to have low local structure metrics (Supplementary Figures 8–37, see Supplementary Data available online at https://academic.oup.com/bib), and is prone to separate the same type of cells, or different types of cells with a gradual transition, into discrete clusters. A few examples include the separation of the mesenchymal cells in the Tabula Muris trachea dataset (Supplementary Figure 15, see Supplementary Data available online at https://academic.oup.com/bib); the pachytene spermatocytes, round spermatids, elongating spermatids and elongated spermatids in the Tabula Microcebus testes dataset from lemur 4 (Supplementary Figure 35, see Supplementary Data available online at https://academic.oup.com/bib); other examples are shown in Supplementary Table and Supplementary Figures 13, 16, 20 and 27 (see Supplementary Data available online at https://academic.oup.com/bib). MNN is prone to separate cells into small clusters and showed low ARIs (Supplementary Table and Supplementary Figures 16–19 and 32, see Supplementary Data available online at https://academic.oup.com/bib). Harmony also suffers from inappropriate separation (Supplementary Table and Supplementary Figures 15, 16, 18, 21, 29 and 31, see Supplementary Data available online at https://academic.oup.com/bib). BBKNN is weak in separation of different cell types resulting in the lowest ARI in most cases (32 out of 39), including the mammary gland data in Tabula Muris (Figure 2).

Harmony and Seurat are two popular methods with relatively better integration performance over other benchmarked methods. Compared with Harmony, FIRM shows its superiority in integration by achieving the lower mixing metric and higher local structure metric (Supplementary Figure 39, see Supplementary Data available online at https://academic.oup.com/bib). Seurat is the method with the closest performance to FIRM (Supplementary Figure 39, see Supplementary Data available online at https://academic.oup.com/bib). Seurat and FIRM have comparable performance in terms of ASW, but FIRM is superior in terms of ARI. Although Seurat usually has lower mixing metrics, FIRM does not show any obvious deficiency for mixing based on the UMAP plots of the integrated dataset. Considering the trade-off between the mixing metric and local structure metric, FIRM’s higher local structure metric suggests that it is more robust than Seurat in avoiding overcorrection.

### FIRM is robust against overcorrection

Cell atlas projects usually consist of scRNA-seq datasets for a comprehensive set of tissues, often spanning all the organs of an organism. The composition of cell types is largely different across tissues, while some cell types such as immune cells, fat cells and cells of the vasculature are shared between multiple tissues or organs; cross comparison of different cell types and joint analysis of shared cell types across tissues are both valuable and informative. As such, it is essential that integration approaches not only accurately integrate datasets from multiple experiments or technology platforms, but also across different tissues.

Other integration methods, such as Seurat, LIGER and MNN, directly adjust the data matrices so that neighboring cells across different datasets have similar adjusted expression profiles, but this process of adjustment is vulnerable to overcorrection because the cells that are close in distance across datasets may not always be biologically similar. Different from other methods that project reference dataset onto query dataset based on neighboring cells across datasets, FIRM harmonizes datasets by incorporating scaling factors that account for differences in cell type compositions across datasets. As a result, FIRM can avoid overcorrection even if there are no shared cell types across the datasets being integrated, which is particularly important when integrating across multiple tissue types.

To evaluate whether the data integration methods are prone to overcorrection, we use the benchmark methods to integrate two datasets that had shared cell types manually removed, such that they have no cell types in common: SS2 dataset of kidney, and 10X dataset of brain cortex of lemur 2 in Tabula Microcebus [16]. We applied FIRM, Seurat, Harmony, BBKNN, BUSseq, LIGER, Scanorama and MNN to integrate these two datasets (Figure 3); we excluded scVI and ZINB-WaVE from this assessment as these two methods did not work well even when there were shared cell types across datasets. Of all the methods assessed, FIRM and Harmony perfectly separated the cell types from each dataset and achieved high mixing metric. Other methods all suffered from overcorrection to varying degrees. Severe overcorrection was observed in Seurat, BUSseq, LIGER and MNN, where neurons and T cells were incorrectly mixed. These other methods also inappropriately clustered different cell types together, resulting in low ARIs. The advantage of local structure preservation, one of the key strengths of the FIRM approach, is especially beneficial for integration across different tissues.

### FIRM can transfer cell type identity labels across datasets and provide better clustering

By integrating SS2 and 10X datasets, we can take advantage of the strengths of each technology and improve data robustness. 10X datasets have higher throughput and usually more cell types are captured; in the SS2 dataset, some cell types may contain very few cells and fail to be identified if analyzed alone. Based on the SS2-10X integrated dataset, we can transfer information between datasets, such as cell type annotations and identity labels. One way to effectively label cell populations in SS2 data is by transferring the manually annotated 10X cell type identity labels to SS2 cells by detecting nearest neighbors for each SS2 cell in 10X dataset (Materials and methods). For example, in the Tabula Microcebus [16] testes SS2 dataset, we are not able to distinctly identify spermatogonia as there are only a few of them. By incorporating information from the 10X dataset, we identified three spermatogonia in the SS2 dataset that have marker expression patterns (*KIT*, *SOHLH1*, *PHOXF1*, *ZBTB16*) consistent with spermatogonia in the 10X dataset (Supplementary Figure 40, see Supplementary Data available online at https://academic.oup.com/bib). We also noted that some marker genes (*OVOL1*, *SPO11*, *TEX101*) show clearer patterns in the SS2 dataset compared with the 10X dataset, indicating the benefit of detecting low abundance transcripts using SS2. For cases where the SS2 dataset contains more cell types than 10X dataset, we designed match scores such that cells with low scores can be labeled as ‘unknown’ (Materials and methods).

FIRM can be applied to align more than two datasets, such as when harmonizing datasets generated from multiple individuals and platforms for one specific tissue (Materials and methods). After accurate harmonization of multiple datasets, performing clustering on the integrated dataset can provide more reliable and consistent cluster labels for each dataset; taking advantage of the enhanced statistical power of the larger integrated dataset enables identification of rare cell types that may be missed in separated datasets. For example, kidney urothelial cells in the Tabula Microcebus [16] are extremely rare in all the individual datasets: none from lemur 1, 4 cells from lemur 2, 4 cells from lemur 3 and 7 cells from lemur 4. They were not readily identifiable when the kidney datasets were individually annotated. After using FIRM to integrate all the kidney datasets across individuals and platforms, a small cluster of urothelial cells could then be detected with the specific markers (*UPK1A*, *FOXA1* and *UPK3A*) expressed (Figure 4).

### FIRM accurately constructs cell atlases for entire organisms

FIRM’s greatest strength is its accuracy, which is essential when creating reference datasets such as cell atlases. Since different tissue types can naturally vary greatly in their cell-type composition, unbalanced cell type compositions are a common feature of cell atlas datasets. To address this pain point, FIRM’s algorithm prioritizes the accuracy of integration for such unbalanced datasets by the way it calculates alignments between cell clusters from different datasets. As a demonstration of this, we applied FIRM to integrate all the SS2 datasets from three individuals and 20 tissues in the Tabula Microcebus [16] (Supplementary document). We compared the results of FIRM with that of four other popular methods for multiple datasets integration: Seurat, Harmony, BBKNN and Scanorama (Figure 5). In this study, 29 SS2 datasets across individuals or tissues, which contain a total of 12,329 cells, were integrated. The integrated visualizations revealed that FIRM can provide accurate mixing of the shared cell types across both tissues and individuals, while preserving clear separation of various tissue compartments. For example, the germ cells which only exist in the testes dataset in this study can be viewed as a ‘sanity check’. FIRM separated the germ cells from other types of cells while retaining its gradient structure from the original dataset. In contrast, Seurat suffered from severe overcorrection in merging cells from different compartments. Overcorrection also occurred when applying Harmony: a few stromal cells were mixed into the germ cells; some epithelial cells were mixed into megakaryocyte-erythroid cells; some hematopoietic cells were mixed into endothelial cells; and some myeloid cells were mixed into the lymphoid cells. For BBKNN, the tissue compartments were close to each other, including the germ cells. Although Scanorama did not merge germ cells with other cells, the endothelial cells, epithelial cells and stromal cells could not be distinguished from one another in the integrated result. Finally, Seurat faces difficulties integrating multiple large datasets with very small datasets, such as those with fewer than 100 cells, because the numbers of neighbors selected for finding anchors are the same across datasets; therefore, for small datasets, only a small number of neighbors can be chosen, which then greatly affects the effectiveness of integrating other large datasets. The FIRM-integrated data can contain the harmonized expression for all genes when taking the expression values for all genes as the input. However, other methods only make use of a subset of genes, for example the overlapped genes or highly variable genes between datasets. Using a subset of genes for integration means that the integrated result either only contains this subset of genes or is a low-dimensional representation, which limits the applicability of downstream analyses that require full gene expression profiles—including the analysis of differential gene expression between different clusters. This demonstration illustrates FIRM’s capability in performing whole organism atlas integration with superior accuracy.

Alternatively, to further improve efficiency for model organisms such as mouse, where the individual- and organ-specific effects are often negligible, we can directly integrate across-technology data while treating within-technology data as harmonized. As an example, we integrated the Tabula Muris data [12], a multi-tissue dataset for *Mus musculus*, to construct a comprehensive atlas. For this case, we considered all the 44 779 cells profiled using SS2 for all tissues as one dataset, and all the 54 865 cells profiled using 10X for all tissues as the other dataset. We used FIRM to directly integrate these two large datasets (Figure 6 and Supplementary Figure 41, see Supplementary Data available online at https://academic.oup.com/bib). For the shared cell populations across platforms, FIRM shows extensive mixing performance. The tissue-specific cells in that were found only in SS2 data remained correctly unmixed after integration: for example, microglial cells in brain myeloid; oligodendrocytes in brain non-myeloid; cells in large intestine; keratinocyte stem cells in skin.

The FIRM algorithm naturally allows parallelization, because it gradually changes the number of clusters in SS2 and 10X datasets and searches for the combination that gives the best cluster alignment (MATERIALS AND METHODS). Therefore, the computational time of FIRM can be greatly shortened by using more CPU cores. We evaluated the computational time for the SS2-10X integration of Tabula Muris [12] using FIRM and other benchmarked methods (Supplementary Figure 42, see Supplementary Data available online at https://academic.oup.com/bib). The time of FIRM varies from 20 s to half an hour for different tissues with the number of cells ranging from 934 to 12 598 using 30 cores. The time taken to integrate 44 779 cells profiled using SS2 with 54 865 cells profiled using 10X for all tissues in Tabula Muris [12] using FIRM took about 20 min to an hour, varying based on the number of clusters in the 10X dataset.

## Discussion

FIRM is an accurate and effective method for integrating scRNA-seq datasets across multiple tissue types, experiments and platforms. For downstream analysis to be biologically meaningful, it is important to minimize technical variations between datasets such as batch effects while preserving biological variations of interest. Generally, it is very difficult to distinguish technical from biological variation, and overcorrection can occur when attempting to remove technical variation, resulting in loss of critical biological variations. The best way to avoid overcorrection is to design methods that target minimization of specific types of confounding variation. FIRM successfully does so by specifically accounting for the heterogeneity in cell type composition between datasets which is a hurdle in efficient data integration. FIRM not only adjusts for the effect of cell type composition but also preserve the biological differences; whereas other existing integration methods that use a general approach to account for variation between datasets do so by aligning cells with high similarity, and as such they are prone to inadvertently removing the biological differences across individuals as well. In contrast with existing methods, FIRM requires no assumption about shared cell populations between datasets and is therefore applicable even without prior knowledge about the dataset composition.

In the FIRM algorithm, we used PCA for dimension reduction. PCA tries to preserve the global structure instead of the local structure presented in the data. Thus, the differences in cell type composition would influence the PC with the highest variance. We compared the performance of PCA and kernel PCA, finding that kernel PCA is quite sensitive to the parameters in the kernel function and can be time-consuming (see details in Supplementary Document and Supplementary Figures 43–50, see Supplementary Data available online at https://academic.oup.com/bib). In the FIRM algorithm, the reason why we perform dimension reduction is to cluster cells and then align clusters across datasets. PCA is shown to be valid for clustering, as indicated by relatively high ASW and ARI, and computational efficiency. The key problem preventing accurate integration of scRNA-seq datasets is the difference in cell type composition which cannot be easily solved by using other dimension reduction methods such as kernel PCA. Furthermore, the FIRM-integrated data contains the harmonized expression for all genes. For downstream analysis, different dimension reduction methods can be applied on the integrated data, which broadens the applications of FIRM.

Through analysis of a diverse collection of human, mouse and mouse lemur datasets, we show that FIRM outperforms or performs comparably to existing methods in terms of accuracy of integration and superior preservation of original structure for each dataset. FIRM has been adopted as the integration tool in the Tabula Microcebus atlas data integration [16]. Ultimately, our data integration tool enables new biological insights and provides efficiency and utility for large-scale projects.

**Key Points**

- Differences in cell type composition are a major factor preventing accurate integration of scRNA-seq data generated by different technology platforms.
- By accounting for the effects of cell type compositions, FIRM achieves superior integration accuracy of scRNA-seq datasets.
- FIRM is widely applicable to scRNA-seq datasets across multiple tissue types, platforms and experimental batches.
- FIRM provides accurate mixing of shared cell type identities and superior preservation of original structure without overcorrection, generating robust integrated datasets for downstream exploration and analysis.
- FIRM is also a facile way to transfer cell type labels and annotations from one dataset to another.

## Supplementary Material

20220330_Supplementary-document_bbac167

20220330_Supplementary-Table_bbac167

## Data Availability

The datasets in Tabula Muris contains 44 949 cells profiled using SS2 from 20 organs and 55 656 cells profiled using 10X from 12 mouse organs, which are available at http://tabula-muris.ds.czbiohub.org/. We removed cells without cell type annotations. The datasets in Tabula Microcebus are available at https://tabula-microcebus.ds.czbiohub.org/. The Human Lung Atlas data are available on Synapse (https://www.synapse.org/#!Synapse:syn21041850). FIRM is available on GitHub at https://github.com/mingjingsi/FIRM.

## Author Biographies

**Jingsi Ming** is an assistant professor at the Academy of Statistics and Interdisciplinary Sciences, KLATASDS-MOE, East China Normal University. Before she joined ECNU, she was a postdoctoral fellow at the Department of Mathematics, The Hong Kong University of Science and Technology.

**Zhixiang Lin** is an assistant professor at the Department of Statistics, The Chinese University of Hong Kong, Hong Kong.

**Jia Zhao** is a graduate student at the Department of Mathematics, The Hong Kong University of Science and Technology, Hong Kong.

**Xiang Wan** is a senior research scientist at the Shenzhen Research Institute of Big Data, Shenzhen, China. His research interests include meta-analysis and statistical learning.

The Tabula Microcebus Consortium full list of members and their affiliations can be found in the Supplemental Notes.

**Can Yang** is an associate professor at the Department of Mathematics, The Hong Kong University of Science and Technology, Hong Kong.

**Angela Ruohao Wu** is an assistant professor at the Division of Life Science and Department of Chemical and Biological Engineering, The Hong Kong University of Science and Technology, Hong Kong.
