# Supplementary material for: FIRM: Flexible integration of single-cell RNA-sequencing data for large-scale multi-tissue cell atlas datasets
Source: Brief Bioinform. 2022 May 14;23(5):bbac167. doi: 10.1093/bib/bbac167 (PMC9487597; doi:10.1093/bib/bbac167)
Supplement: 20220330_Supplementary-document_bbac167 [file 20220330_supplementary-document_bbac167.pdf]

Supplementary document for

FIRM: Flexible Integration of single-cell RNA-sequencing data for large-scale Multi-tissue cell atlas datasets

Variability of UMAP

To checked the variability of UMAP plots on the same dataset, we used the data in the left top panel of Figure 1d in the main text. Supplementary Fig. 1 shows four UMAP plots on this data using different random seeds. Although the shapes of UMAP plots were slightly different, the mixing performance was similar, i.e., the basal cells were not mixed well across datasets.

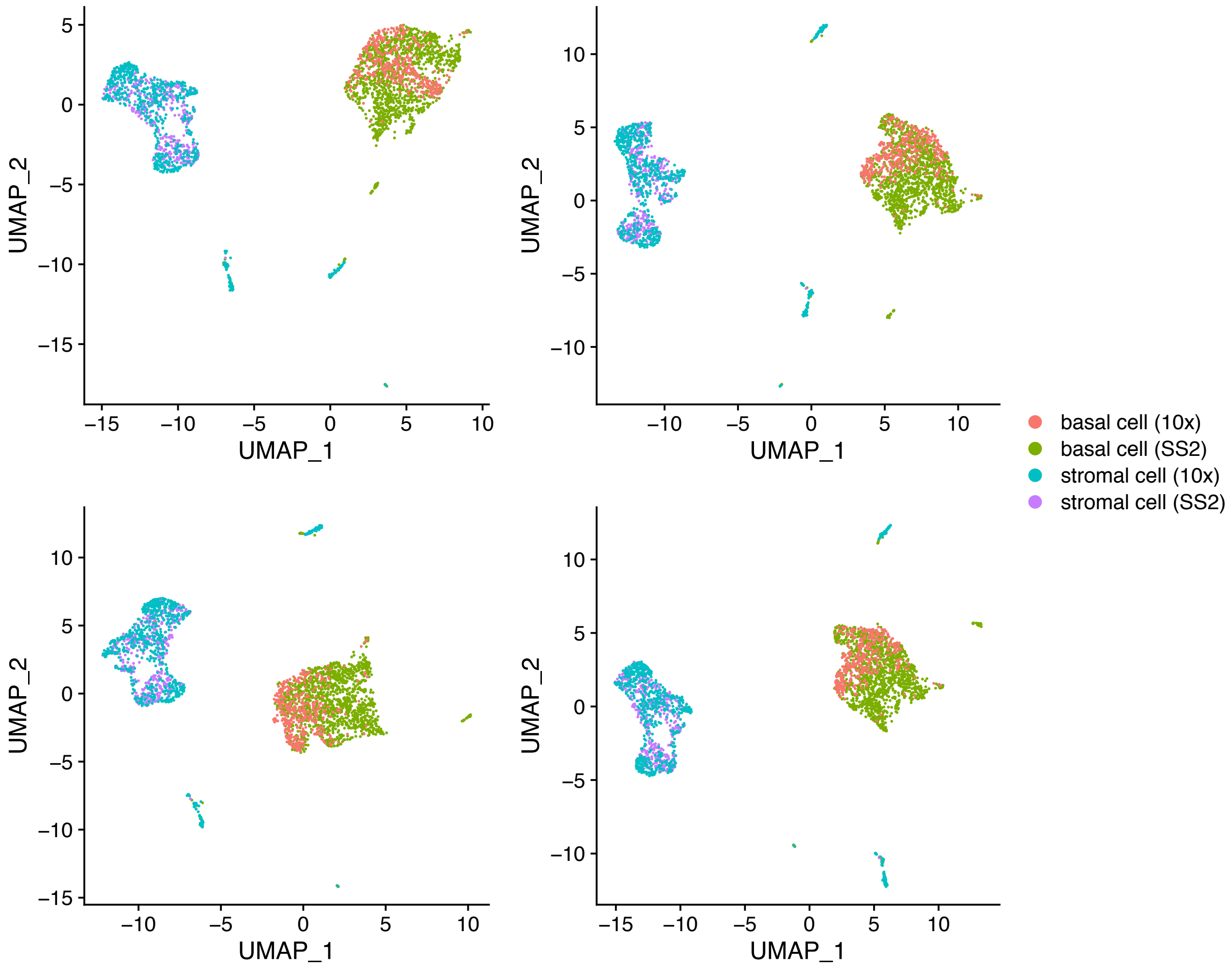

Supplementary Fig. 1 UMAP plots for the mammary gland data from Tabula Muris [1], withholding only the basal cells and stromal cells, using different random seeds.

In Figure 1d, we conducted subsampling to gradually reduce the proportion of basal cells in SS2 dataset, which introduced the randomness in generating subsets of SS2 dataset. To check the variability of UMAP plots due to this kind of randomness, we considered the case in the right bottom panel of Figure 1d, where the proportion of basal cells in SS2 dataset was reduced to 35%. Supplementary Fig. 2 shows four UMAP plots based on the subsets of SS2 dataset generated using different random seeds, showing that the mixing performance is consistent.

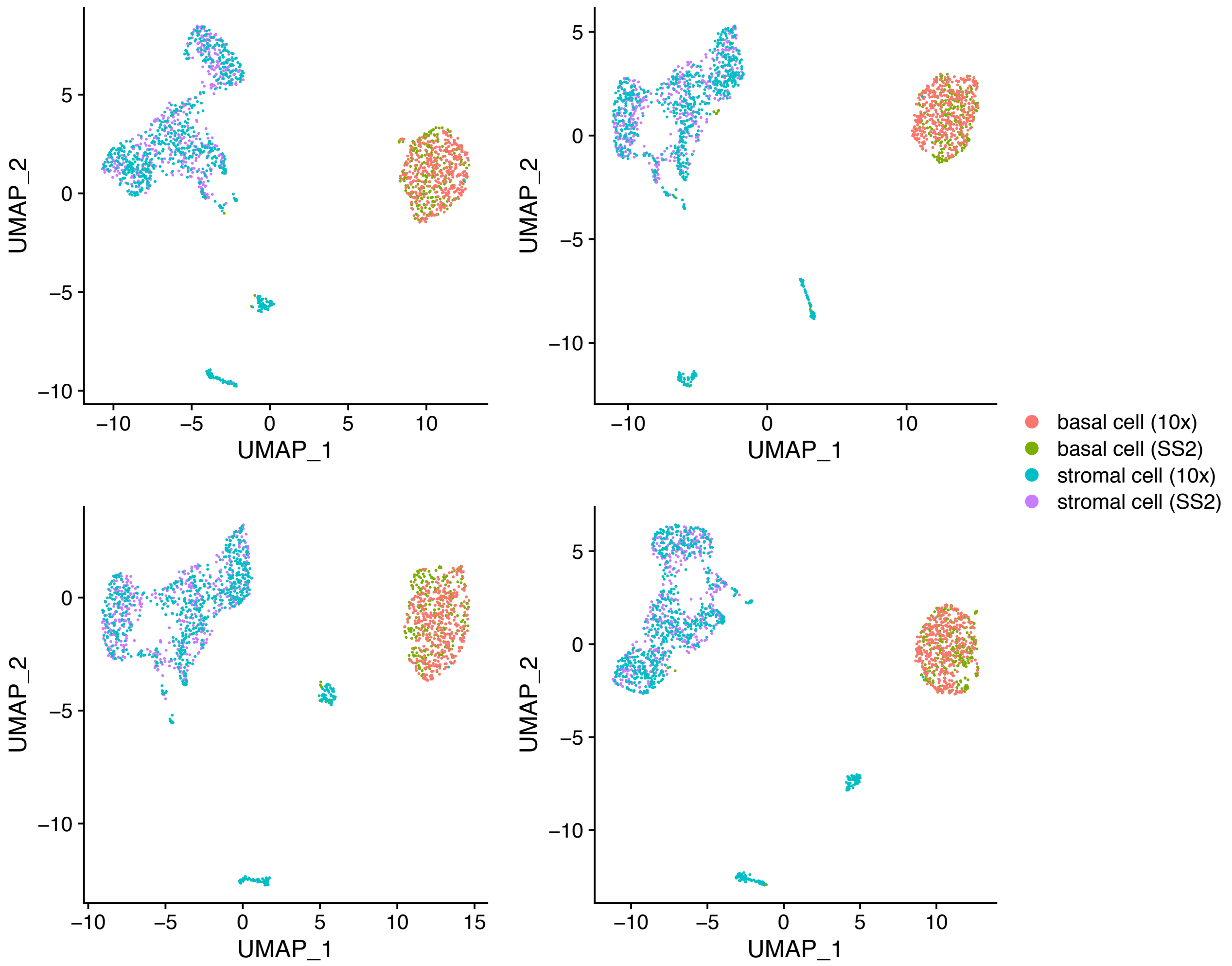

Supplementary Fig. 2 UMAP plots for the case where the proportion of basal cells in SS2 dataset was reduced to 35% in the mammary gland data from Tabula Muris, with only the basal cells and stromal cells. Four UMAP plots were based on the subsets of SS2 dataset generated using different random seeds.

Evaluation metrics

The integration performance is evaluated by four metrics: mixing metric, local structure metric,

average silhouette width (ASW) and adjusted rand index (ARI).

Mixing metric designed in Seurat [2] is used to evaluate how well the datasets mixed after integration. If the local neighborhood for a cell is well mixed across datasets, at least a small number ($k = 5$) of cells from each dataset is assumed to be its neighbors. For each cell, we obtain its ($k.max = 300$) ranked nearest neighbors in the integrated dataset and record the rank of the 5th nearest neighbor in each dataset (with a max of 300). The average of the ranks across all cells is defined as the mixing metric. As a result, smaller mixing metric typically indicates better mixing.

Local structure metric designed in Seurat [2] is used to determine how well the original structure of each dataset was reserved after integration. For each cell, we compare its $k = 20$ nearest neighbors in the original dataset and the integrated dataset. The average value of the fraction of overlapped neighbor across all cells is defined as the local structure metric. A large local structure metric indicates good preservation.

ASW is the mean of silhouette widths across all cells. The silhouette width for cell *i* from cell type *c* is $s_i = \frac{b_i - a_i}{\max\{a_i, b_i\}}$, where a_i is the average distance from cell *i* to all cells in cell type *c*, b_i is lowest value of average distances from cell *i* to all cells for each cell type other than *c*. A higher score of ASW indicates cells are closer to cells of the same cell type and are further from cells of different cell types. We calculated ASW based on the predefined cell identities and low-dimensional embedding space of the integrated dataset using PCA.

ARI measures the similarity between two clustering results, which is defined as $\mathrm{ARI} = \frac{\sum_{ij}\binom{n_{ij}}{2} - \left[\sum_i \binom{a_i}{2} \sum_j \binom{b_j}{2}\right] / \binom{n}{2}}{\frac{1}{2}\left[\sum_i \binom{a_i}{2} + \sum_j \binom{b_j}{2}\right] - \left[\sum_i \binom{a_i}{2} \sum_j \binom{b_j}{2}\right] / \binom{n}{2}}$, where n_{ij}, a_i, b_j are values from the contingency table. For the integrated dataset, we clustered cells based on their PCA scores using the clustering approach in Seurat with default settings. Then we calculated ARI to compare the clustering of integrated data with the predefined cell types, where higher values indicate higher similarities.

As these metrics represent different aspects of performance, joint consideration is required for effective comparison. For example, there is a trade-off between the mixing metric and the local structure metric – a low mixing metric does not always mean accurate integration, since overcorrection is characterized by a low mixing metric and a low local structure metric.

Graphical illustration of FIRM algorithm

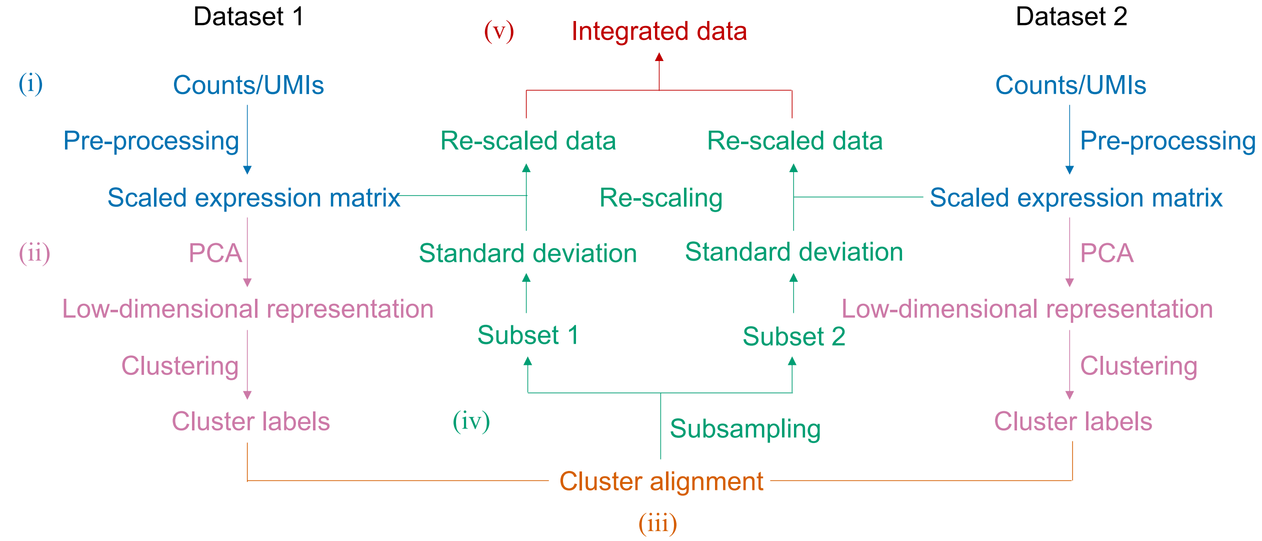

Supplementary Fig. 3 Graphical illustration of the FIRM algorithm.

Sensitivity of FIRM to the number of PCs

In order to check the stability of FIRM with respect to this hyperparameter, we applied FIRM to integrate the mammary gland data generated using SS2 and 10X from Tabula Muris with varied numbers of PCs in {10, 15, 20, 25}. The FIRM-integrated data contains the harmonized expression for all genes. To make the performance comparable, we further conducted PCA with 15 PCs, which is used in our paper, on the integrated data and evaluated their integration metrics. As shown in Supplementary Fig. 4, the performance of FIRM is insensitive to the number of PCs. Similar results are shown in Supplementary Fig. 5 for the limb muscle data from Tabula Muris.

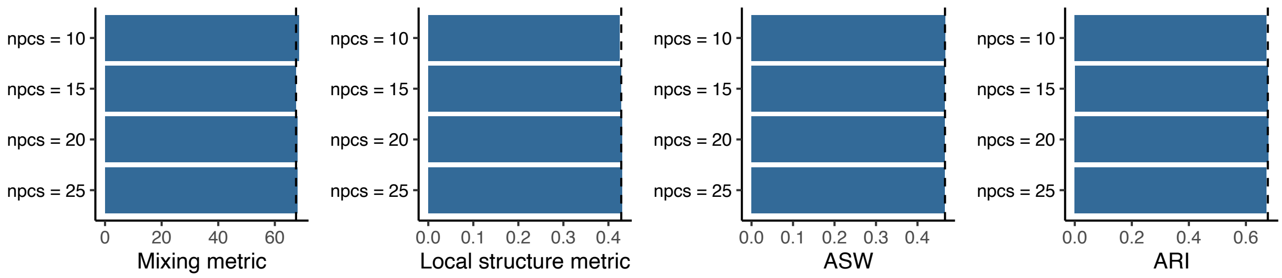

Supplementary Fig. 4 The metrics (mixing metric, local structure metric, ASW and ARI) for evaluating the integration performance of the mammary gland data from Tabula Muris using different numbers of PCs. The dashed lines were set at the values using 15 PCs, which is used in our paper, as reference lines.

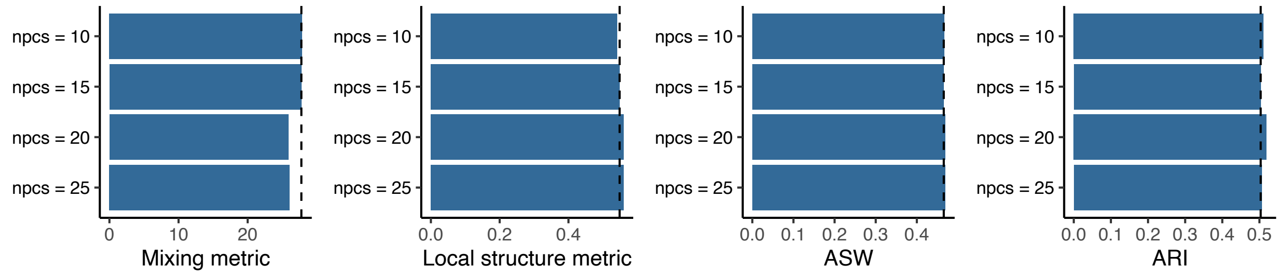

Supplementary Fig. 5 The metrics (mixing metric, local structure metric, ASW and ARI) for evaluating the integration performance of the limb muscle data from Tabula Muris using different numbers of PCs. The dashed lines were set at the values using 15 PCs, which is used in our paper, as reference lines.

Sensitivity of FIRM to the number of nearest neighbors

We checked the sensitivity of FIRM for different numbers of nearest neighbors which varied in {10, 20, 30, 40} on the mammary gland (Supplementary Fig. 6) and limb muscle (Supplementary Fig. 7) datasets, showing that FIRM is stable with respect to this parameter.

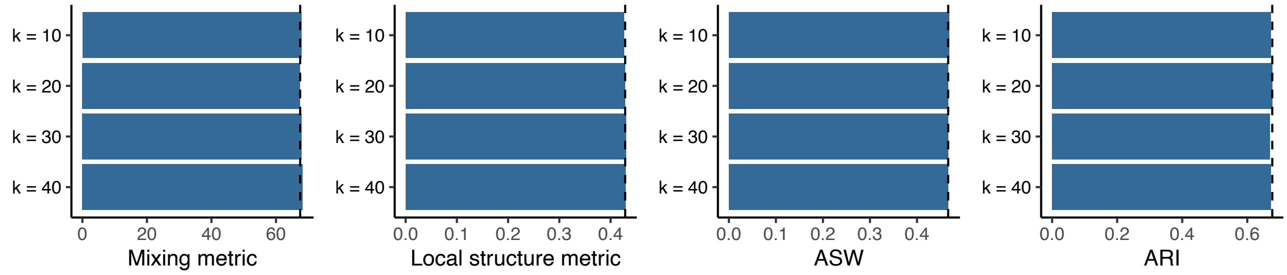

Supplementary Fig. 6 The metrics (mixing metric, local structure metric, ASW and ARI) for evaluating the integration performance of the mammary gland data from Tabula Muris using different numbers of nearest neighbors. The dashed lines were set at the values using 20 nearest neighbors, which is the default value, as reference lines.

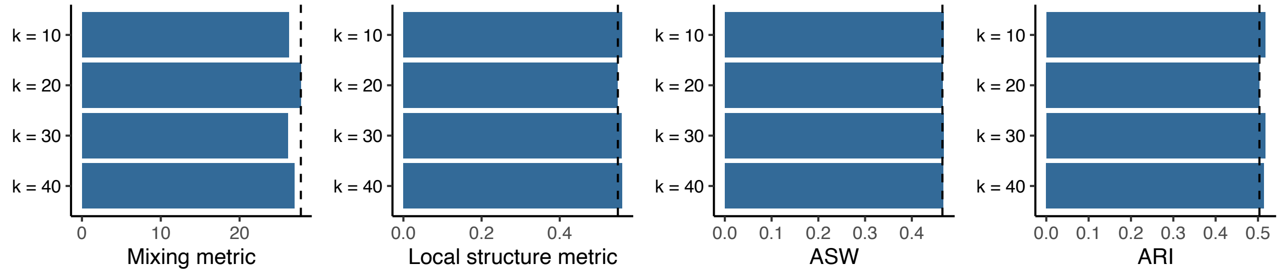

Supplementary Fig. 7 The metrics (mixing metric, local structure metric, ASW and ARI) for evaluating the integration performance of the limb muscle data from Tabula Muris using different numbers of nearest neighbors. The dashed lines were set at the values using 20 nearest neighbors, which is the default value, as reference lines.

Integration of paired SS2 and 10X scRNA-seq datasets

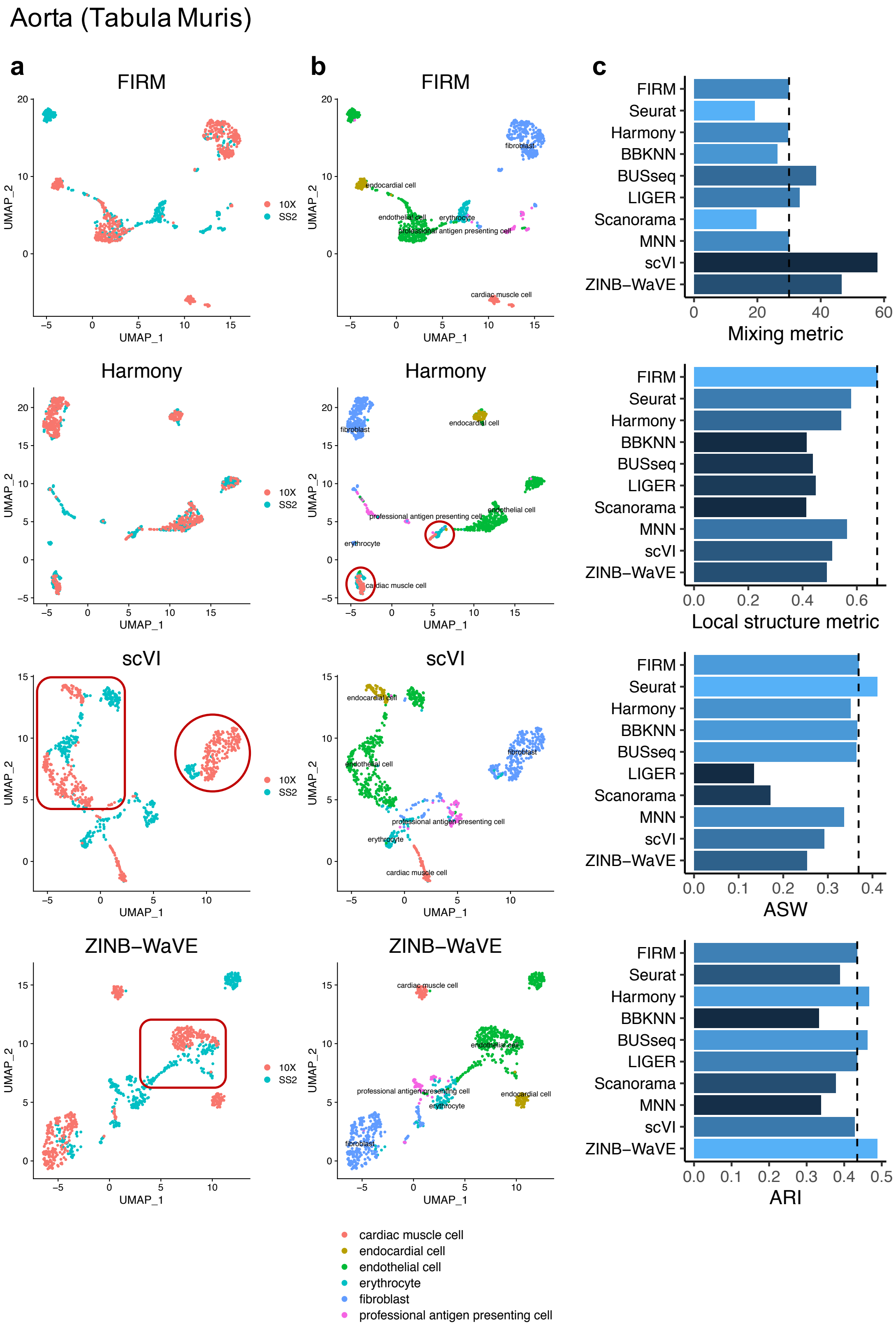

Supplementary Fig. 8 Comparison of integration methods based on the aorta scRNA-seq datasets generated by SS2 and 10X from Tabula Muris. a, **b**, UMAP plots of the integrated scRNA-seq dataset colored by platform (**a**) and by cell type (**b**) using FIRM, Harmony, scVI and ZINB-WaVE. The red circles highlight

the problems of the integration results given by these methods. **c**, Metrics (mixing metric, local structure metric, ASW and ARI) for evaluating performance across the ten methods. The color (from light to dark) represents the performance (from the best to the worst). The dashed lines were set at the values for FIRM as reference lines.

Bladder (Tabula Muris)

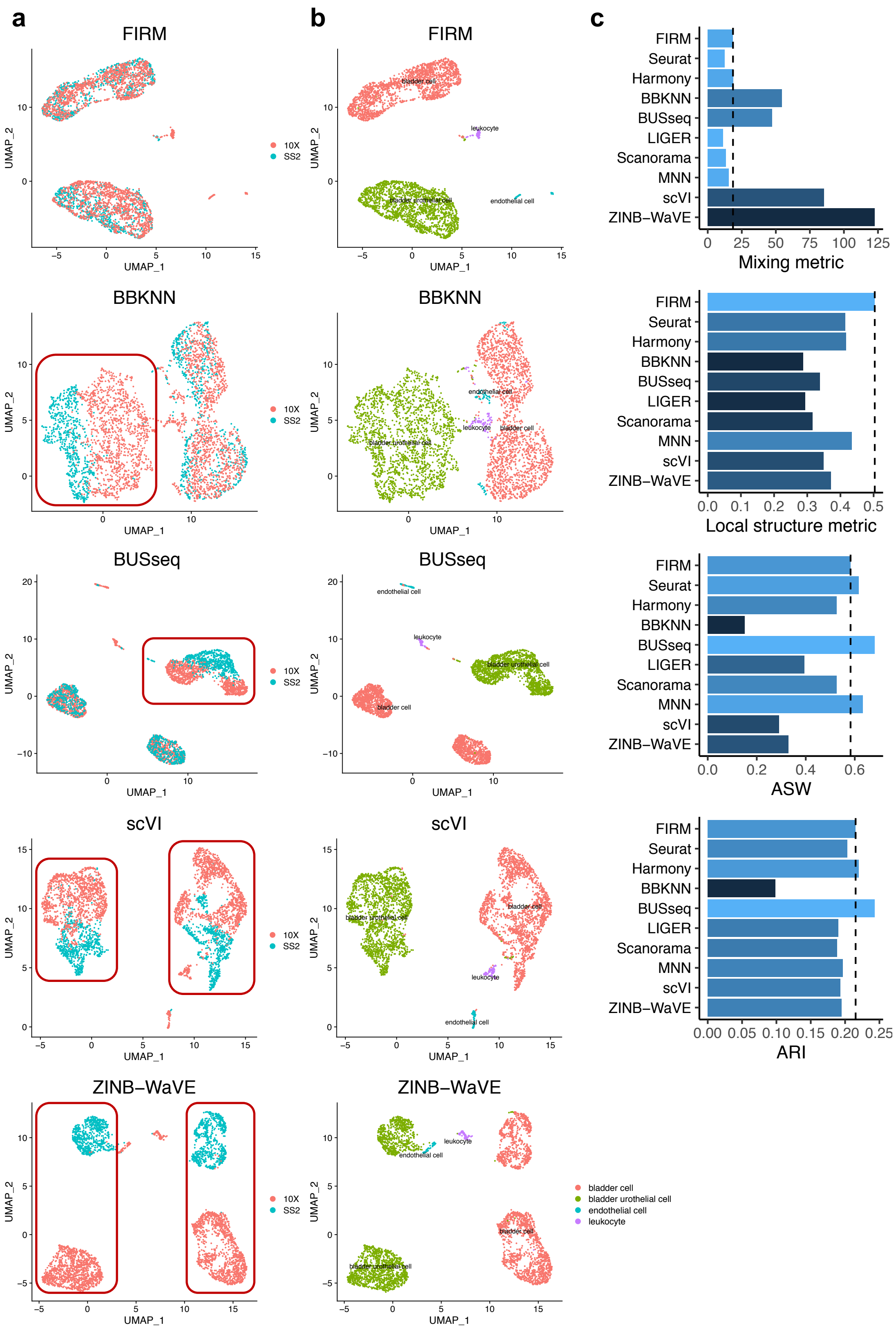

Supplementary Fig. 9 Comparison of integration methods based on the bladder scRNA-seq datasets generated by SS2 and 10X from Tabula Muris. a, **b**, UMAP plots of the integrated scRNA-seq dataset colored by platform

(**a**) and by cell type (**b**) using FIRM, BBKNN, BUSseq, scVI and ZINB-WaVE. The red circles highlight the problems of the integration results given by these methods. **c**, Metrics (mixing metric, local structure metric, ASW and ARI) for evaluating performance across the ten methods. The color (from light to dark) represents the performance (from the best to the worst). The dashed lines were set at the values for FIRM as reference lines.

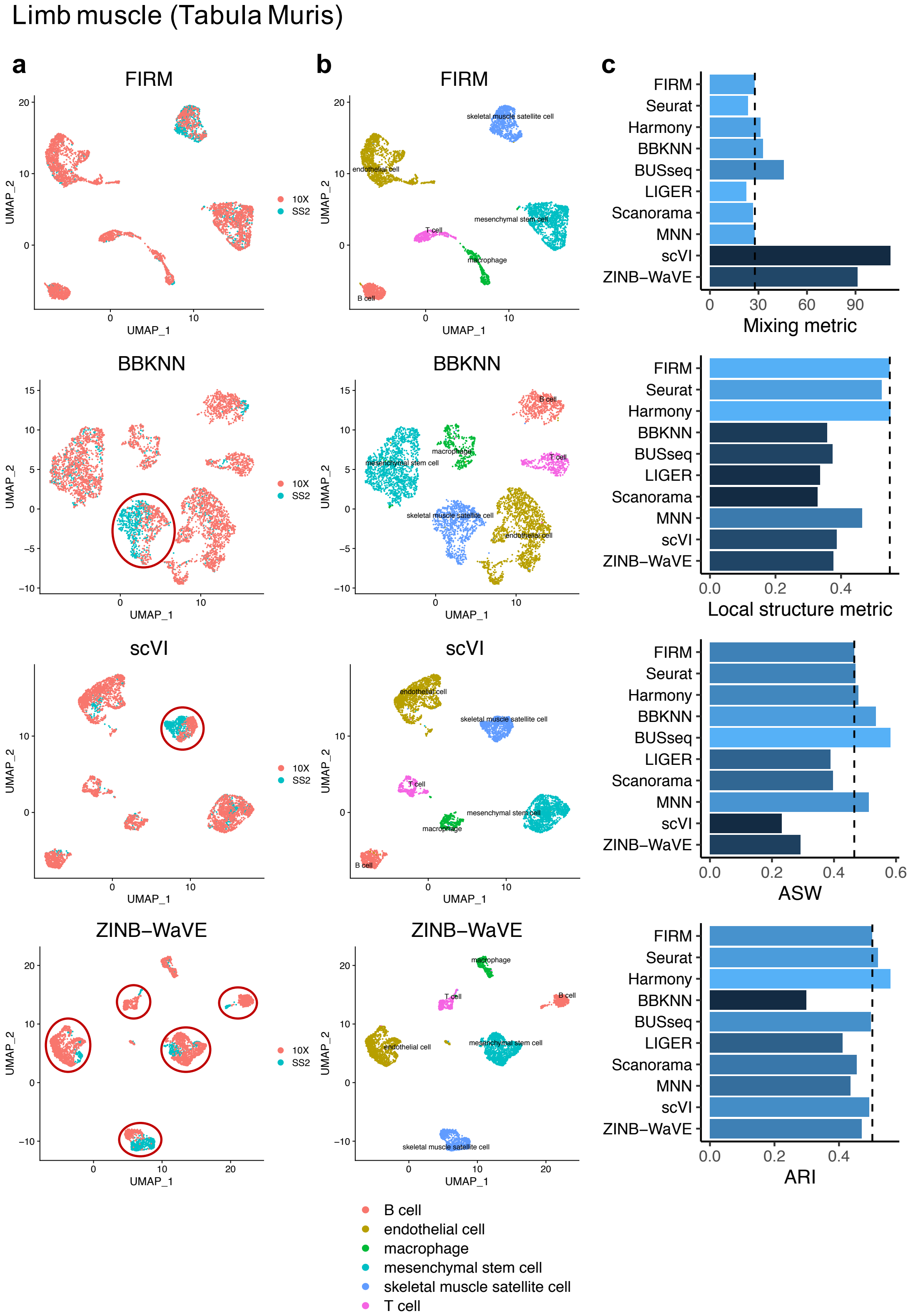

Supplementary Fig. 10 Comparison of integration methods based on the limb muscle scRNA-seq datasets generated by SS2 and 10X from Tabula Muris. a, **b**, UMAP plots of the integrated scRNA-seq dataset colored by platform (**a**) and by cell type (**b**) using FIRM, BBKNN, scVI and ZINB-WaVE. The red circles highlight the problems of the integration results given by these methods. **c**, Metrics (mixing metric, local structure metric, ASW and ARI) for evaluating performance across the ten methods. The color (from light to dark) represents the

performance (from the best to the worst). The dashed lines were set at the values for FIRM as reference lines.

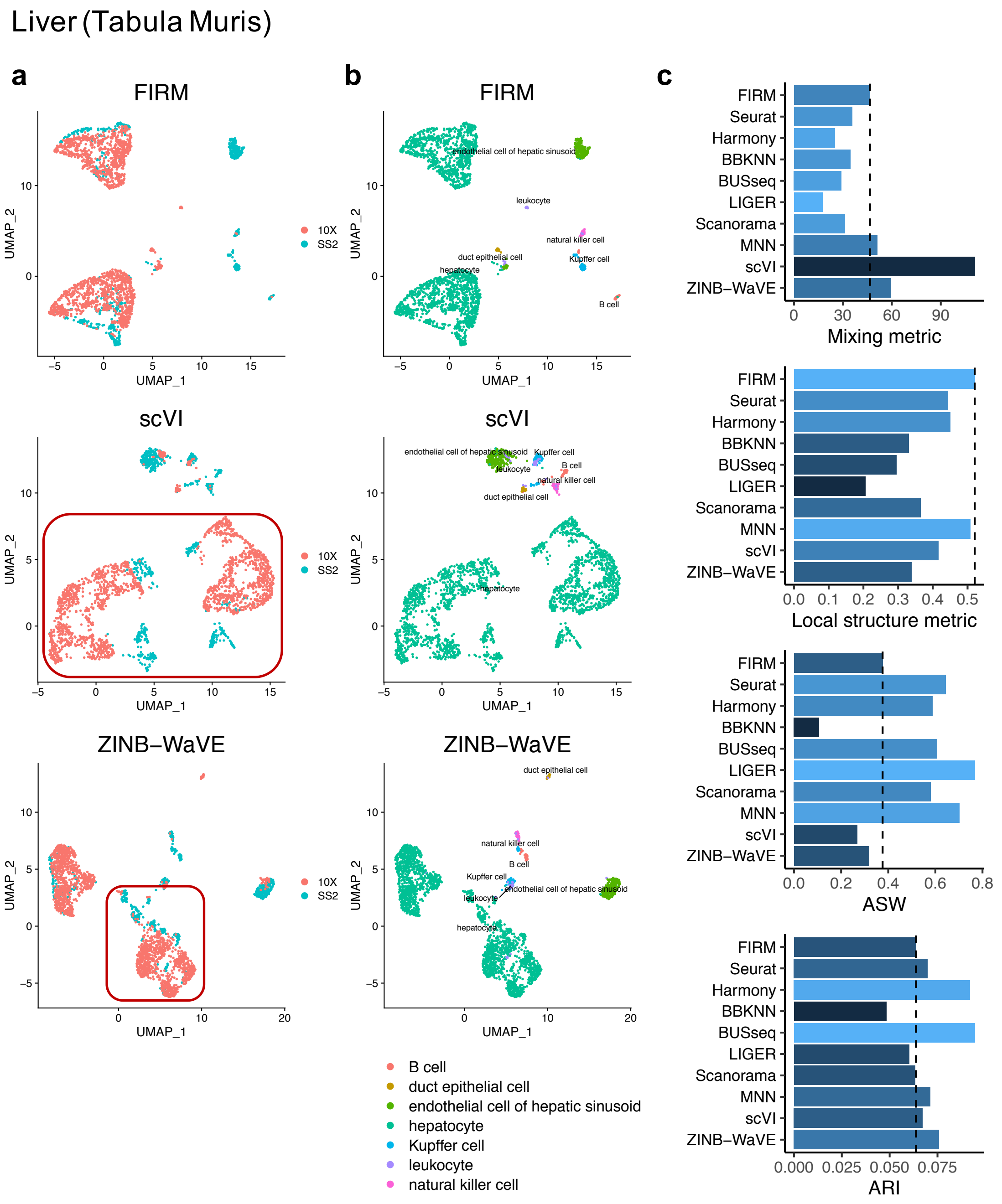

Supplementary Fig. 11 Comparison of integration methods based on the liver scRNA-seq datasets generated by SS2 and 10X from Tabula Muris. a, **b**, UMAP plots of the integrated scRNA-seq dataset colored by platform (**a**) and by cell type (**b**) using FIRM, scVI and ZINB-WaVE. The red circles highlight the problems of the integration results given by these methods. **c**, Metrics (mixing metric, local structure metric, ASW and ARI) for evaluating performance across the ten methods. The color (from light to dark) represents the performance (from the best to the worst). The dashed lines were set at the values for FIRM as reference lines.

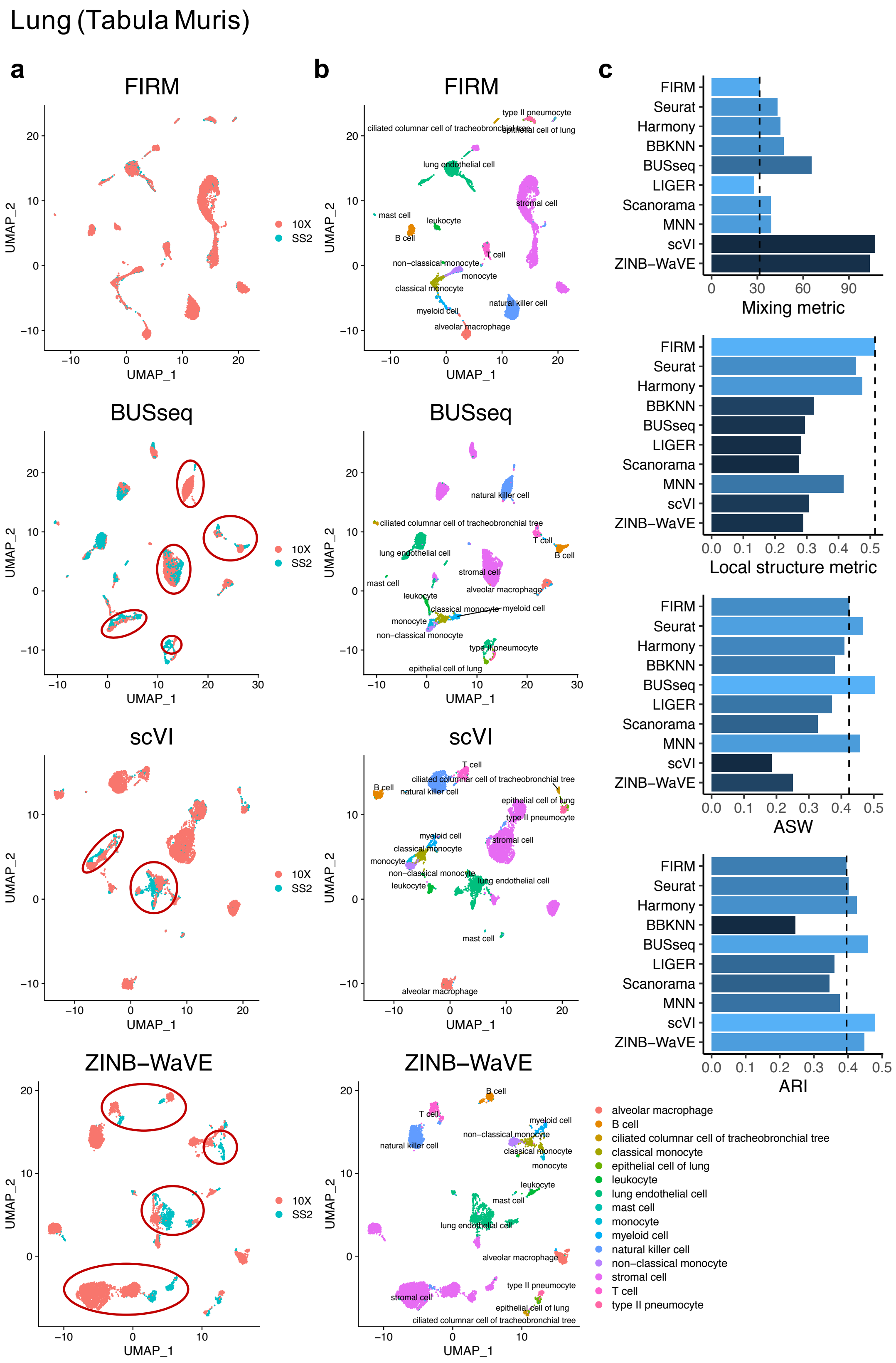

Supplementary Fig. 12 Comparison of integration methods based on the lung scRNA-seq datasets generated by SS2 and 10X from Tabula Muris. a, **b**, UMAP plots of the integrated scRNA-seq dataset colored by platform (**a**) and by cell type (**b**) using FIRM, BUSseq, scVI and ZINB-WaVE. The red circles highlight the problems of

the integration results given by these methods. **c**, Metrics (mixing metric, local structure metric, ASW and ARI) for evaluating performance across the ten methods. The color (from light to dark) represents the performance (from the best to the worst). The dashed lines were set at the values for FIRM as reference lines.

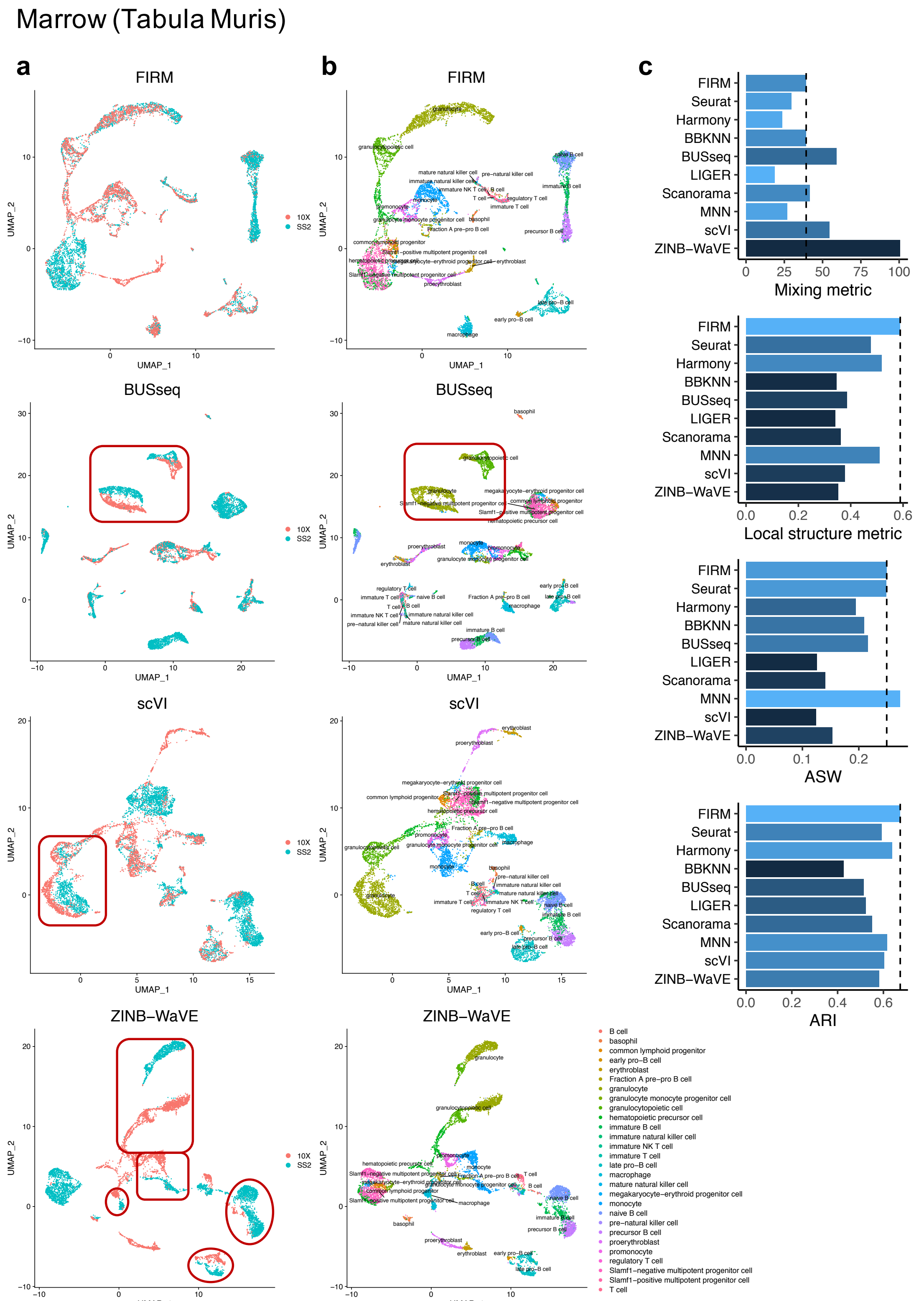

Supplementary Fig. 13 Comparison of integration methods based on the marrow scRNA-seq datasets generated by SS2 and 10X from Tabula Muris. a, **b**, UMAP plots of the integrated scRNA-seq dataset colored by platform (**a**) and by cell type (**b**) using FIRM, BUSseq, scVI and ZINB-WaVE. The red circles highlight the problems of the integration results given by these methods. **c**, Metrics (mixing metric, local structure metric, ASW and ARI) for evaluating performance across the ten methods. The color (from light to dark) represents the

performance (from the best to the worst). The dashed lines were set at the values for FIRM as reference lines.

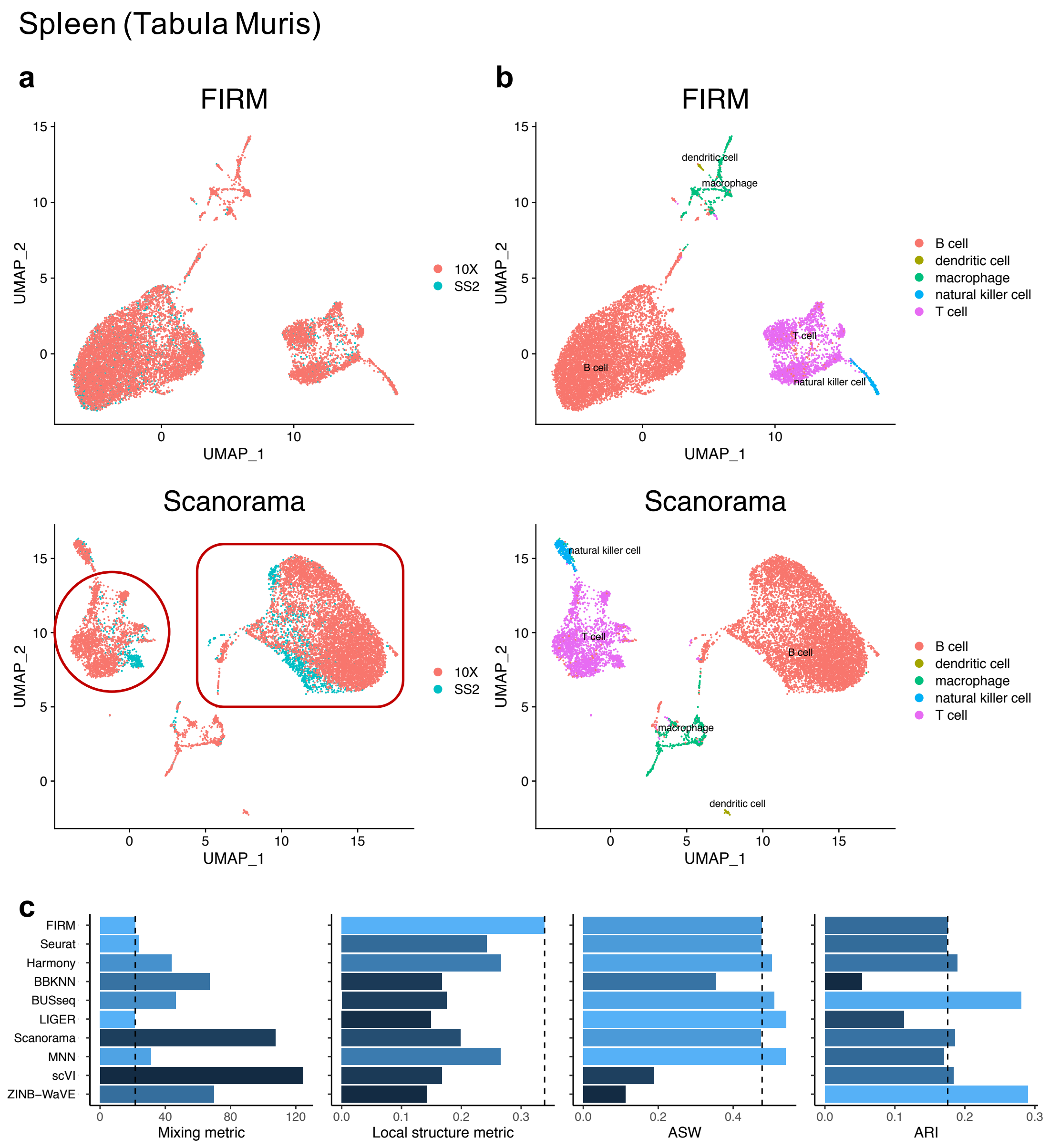

Supplementary Fig. 14 Comparison of integration methods based on the spleen scRNA-seq datasets generated by SS2 and 10X from Tabula Muris. a, **b**, UMAP plots of the integrated scRNA-seq dataset colored by platform (**a**) and by cell type (**b**) using FIRM and Scanorama. The red circles highlight the problems of the integration results given by these methods. **c**, Metrics (mixing metric, local structure metric, ASW and ARI) for evaluating performance across the ten methods. The color (from light to dark) represents the performance (from the best to the worst). The dashed lines were set at the values for FIRM as reference lines.

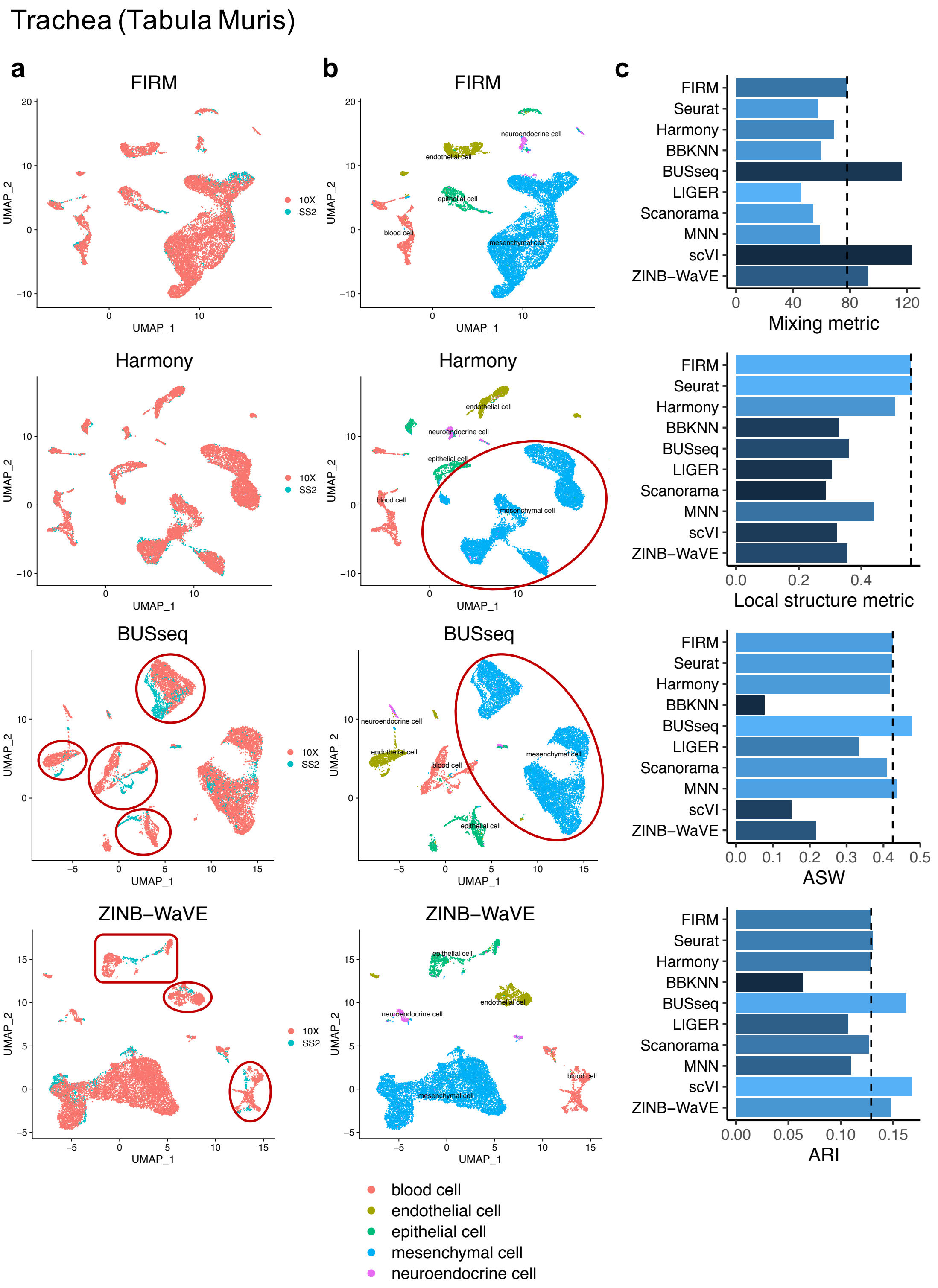

Supplementary Fig. 15 Comparison of integration methods based on the trachea scRNA-seq datasets generated by SS2 and 10X from Tabula Muris. a, **b**, UMAP plots of the integrated scRNA-seq dataset colored by platform (**a**) and by cell type (**b**) using FIRM, Harmony, BUSseq and ZINB-WaVE. The red circles highlight the problems of the integration results given by these methods. **c**, Metrics (mixing metric, local structure metric, ASW and ARI) for evaluating performance across the ten methods. The color (from light to dark) represents the performance (from the best to the worst). The dashed lines were set at the values for FIRM as reference lines.

Blood (lemur 1, Tabula Microcebus)

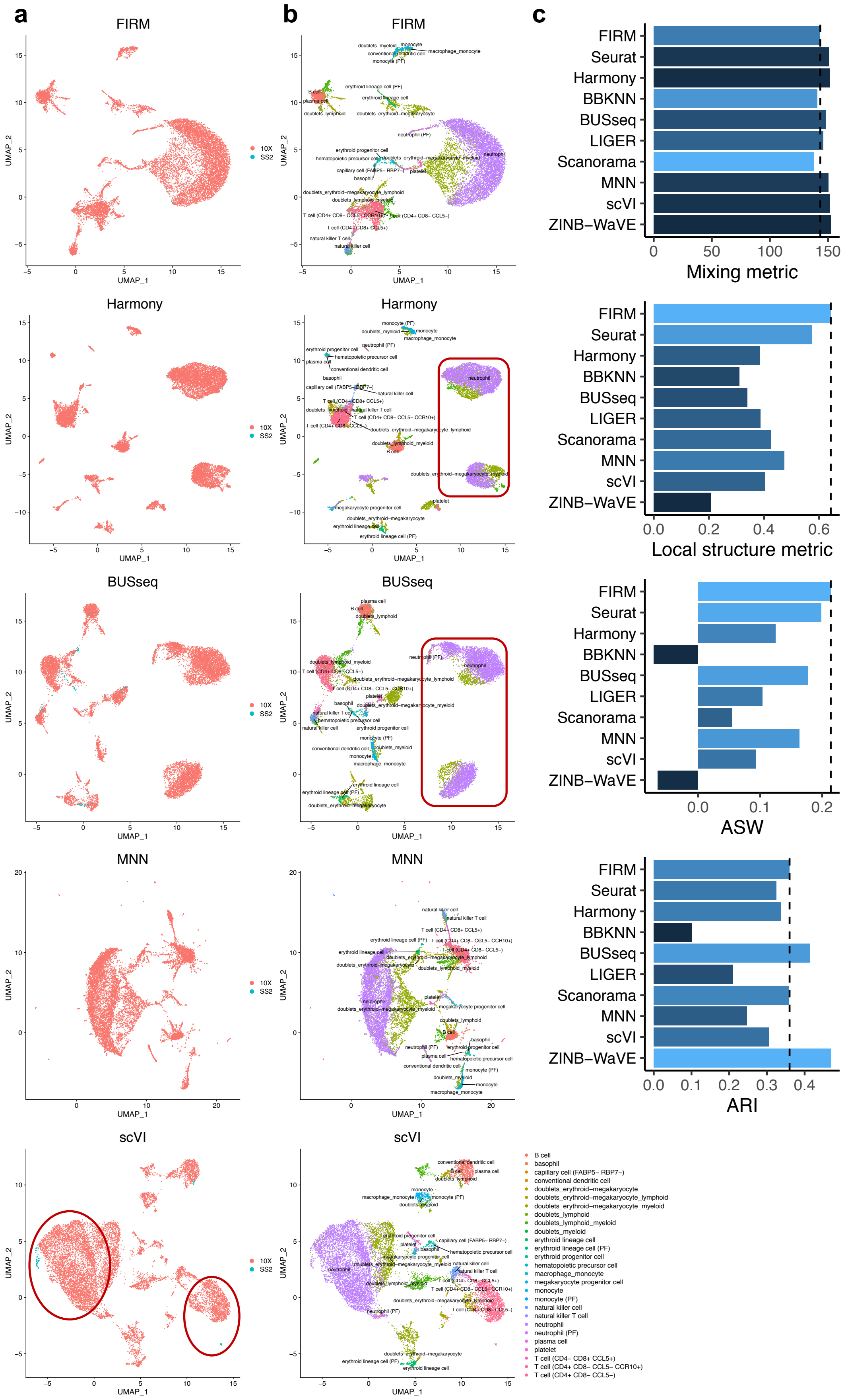

Supplementary Fig. 16 Comparison of integration methods based on the blood scRNA-seq datasets generated by SS2 and 10X from lemur 1 in Tabula Microcebus [3]. **a**, **b**, UMAP plots of the integrated scRNA-seq dataset colored by platform (**a**) and by cell type (**b**) using FIRM, Harmony, BUSseq, MNN and scVI. The red circles highlight the problems of the integration results given by these methods. **c**, Metrics (mixing metric, local structure metric, ASW and ARI) for evaluating performance across the ten methods. The color (from light to dark) represents the performance (from the best to the worst). The dashed lines were set at the values for FIRM as reference lines.

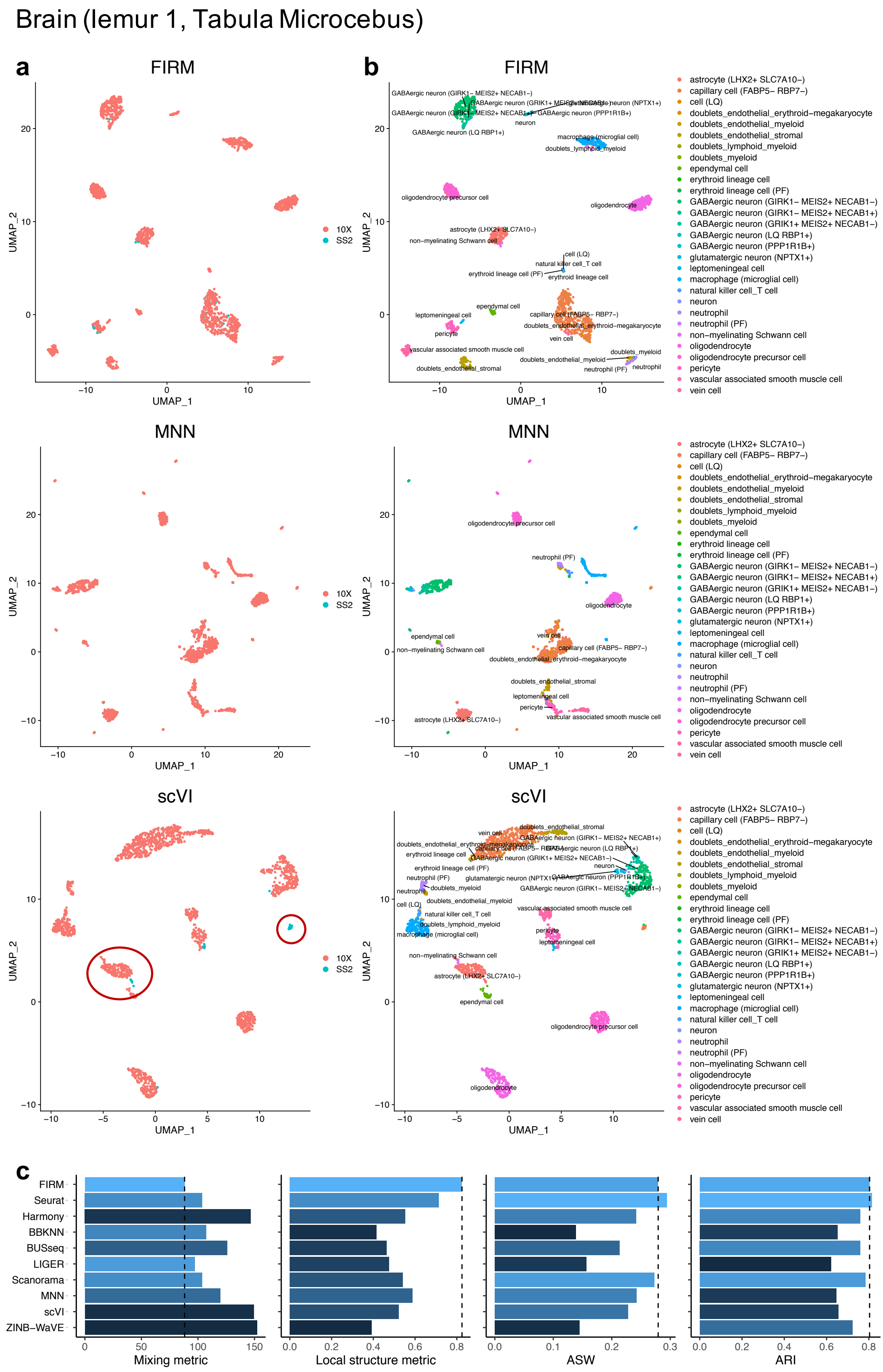

Supplementary Fig. 17 Comparison of integration methods based on the brain scRNA-seq datasets generated by SS2 and 10X from lemur 1 in Tabula Microcebus. a, **b**, UMAP plots of the integrated scRNA-seq dataset

colored by platform (**a**) and by cell type (**b**) using FIRM, MNN and scVI. The red circles highlight the problems of the integration results given by these methods. **c**, Metrics (mixing metric, local structure metric, ASW and ARI) for evaluating performance across the ten methods. The color (from light to dark) represents the performance (from the best to the worst). The dashed lines were set at the values for FIRM as reference lines.

Lung (lemur 1, Tabula Microcebus)

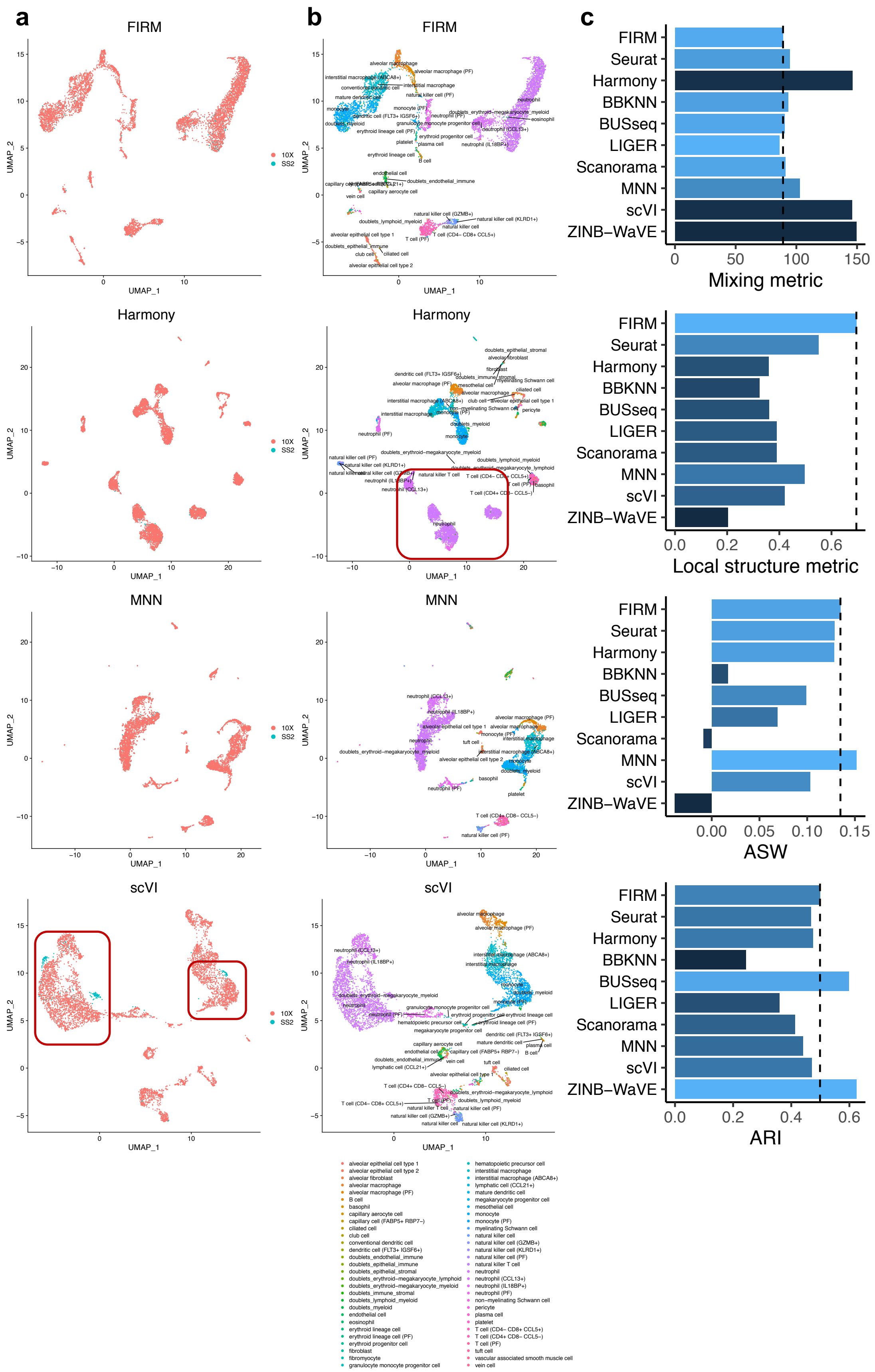

Supplementary Fig. 18 Comparison of integration methods based on the lung scRNA-seq datasets generated by SS2 and 10X from lemur 1 in Tabula Microcebus. **a**, **b**, UMAP plots of the integrated scRNA-seq dataset colored by platform (**a**) and by cell type (**b**) using FIRM, Harmony, MNN and scVI. The red circles highlight the problems of the integration results given by these methods. **c**, Metrics (mixing metric, local structure metric, ASW and ARI) for evaluating performance across the ten methods. The color (from light to dark) represents the performance (from the best to the worst). The dashed lines were set at the values for FIRM as reference lines.

Bladder (lemur 2, Tabula Microcebus)

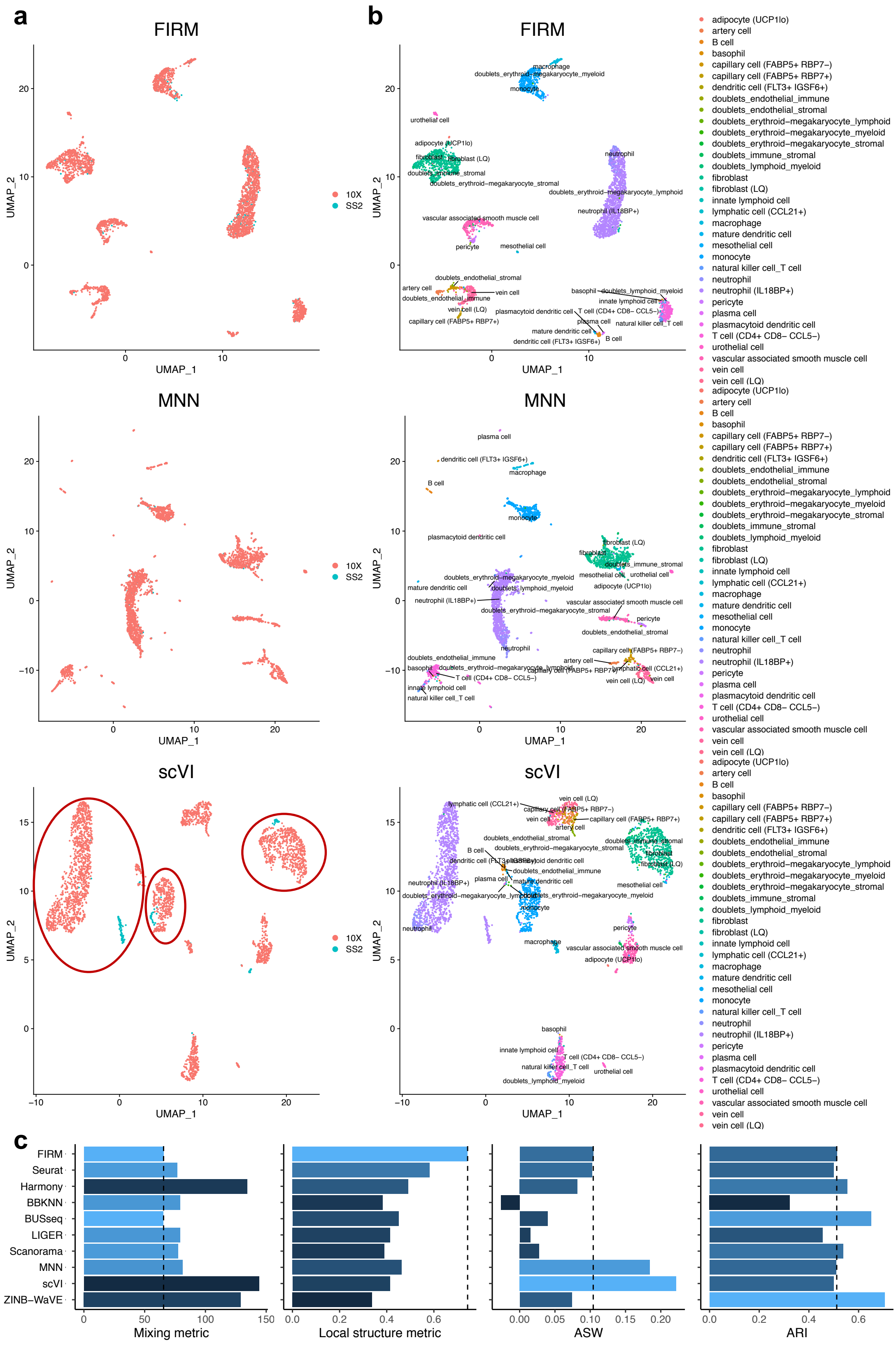

Supplementary Fig. 19 Comparison of integration methods based on the bladder scRNA-seq datasets generated by SS2 and 10X from lemur 2 in Tabula Microcebus. a, b, UMAP plots of the integrated scRNA-seq

dataset colored by platform (**a**) and by cell type (**b**) using FIRM, MNN and scVI. The red circles highlight the problems of the integration results given by these methods. **c**, Metrics (mixing metric, local structure metric, ASW and ARI) for evaluating performance across the ten methods. The color (from light to dark) represents the performance (from the best to the worst). The dashed lines were set at the values for FIRM as reference lines.

Blood (lemur 2, Tabula Microcebus)

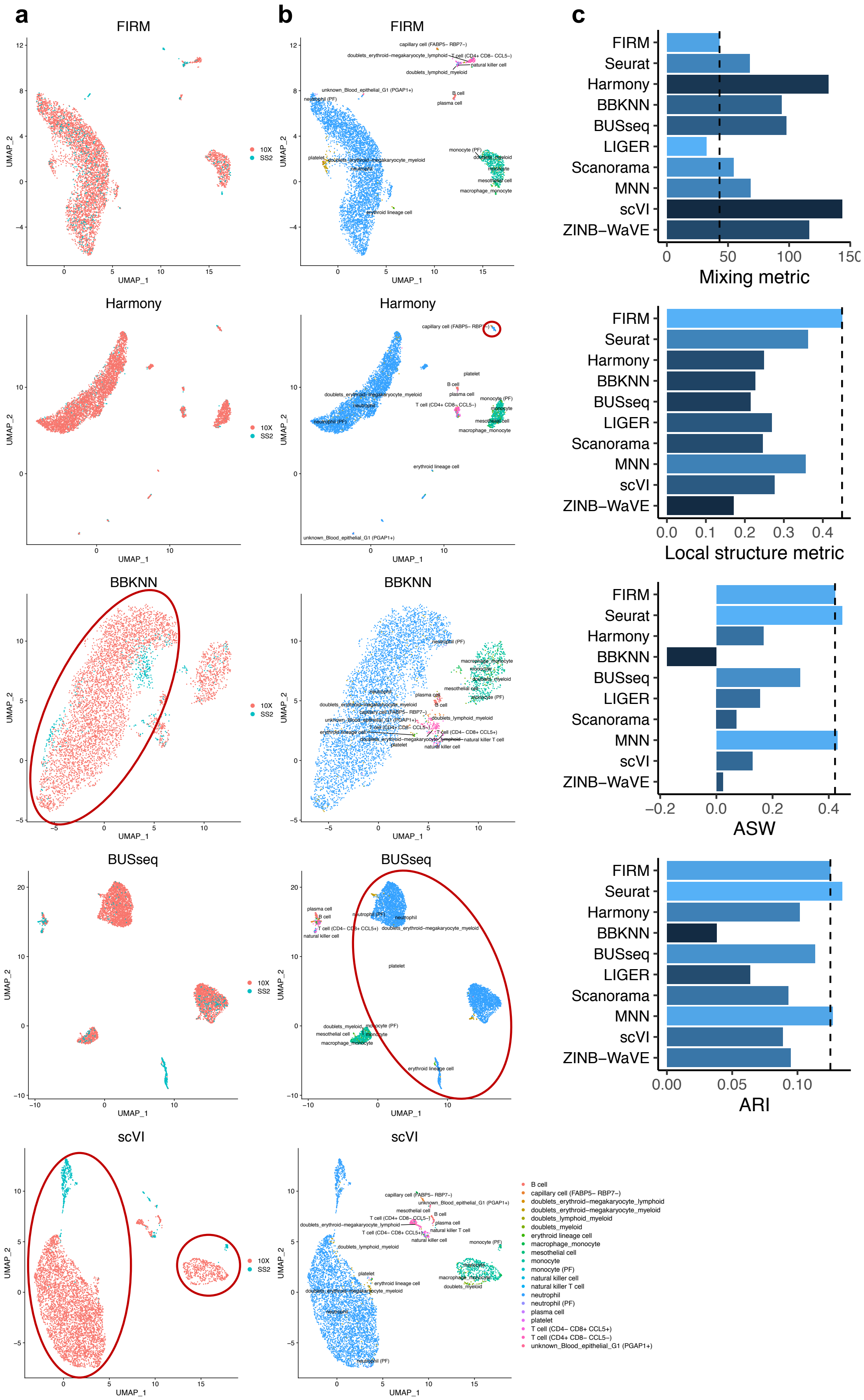

Supplementary Fig. 20 Comparison of integration methods based on the blood scRNA-seq datasets generated by SS2 and 10X from lemur 2 in Tabula Microcebus. a, **b**, UMAP plots of the integrated scRNA-seq dataset colored by platform (**a**) and by cell type (**b**) using FIRM, Harmony, BBKNN, BUSseq and scVI. The red circles highlight the problems of the integration results given by these methods. **c**, Metrics (mixing metric, local structure metric, ASW and ARI) for evaluating performance across the ten methods. The color (from light to dark) represents the performance (from the best to the worst). The dashed lines were set at the values for FIRM as reference lines.

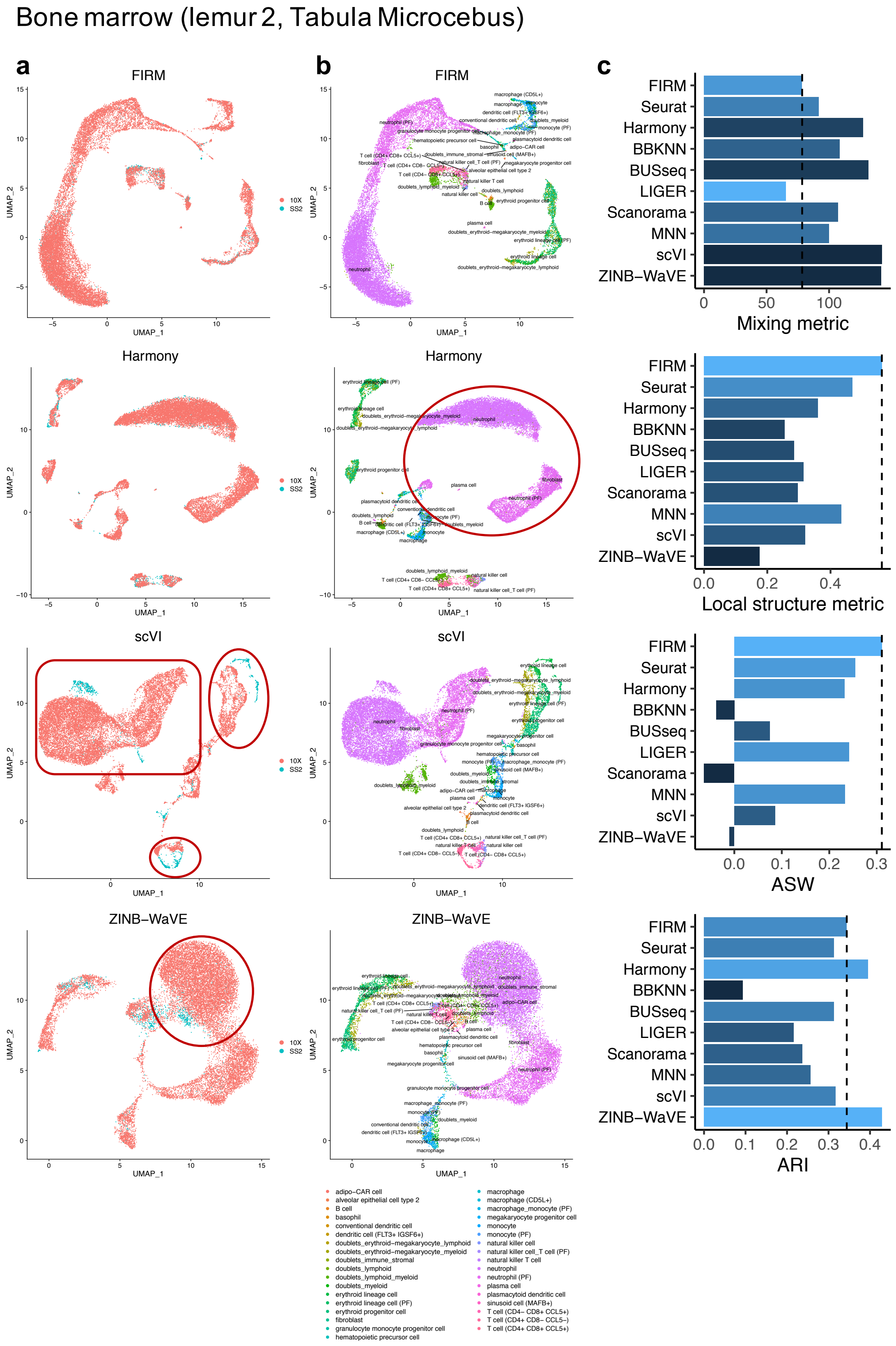

Supplementary Fig. 21 Comparison of integration methods based on the bone marrow scRNA-seq datasets generated by SS2 and 10X from lemur 2 in Tabula Microcebus. a, **b**, UMAP plots of the integrated scRNA-seq dataset colored by platform (**a**) and by cell type (**b**) using FIRM, Harmony, scVI and ZINB-WaVE. The red circles highlight the problems of the integration results given by these methods. **c**, Metrics (mixing metric, local

structure metric, ASW and ARI) for evaluating performance across the ten methods. The color (from light to dark) represents the performance (from the best to the worst). The dashed lines were set at the values for FIRM as reference lines.

Kidney (lemur 2, Tabula Microcebus)

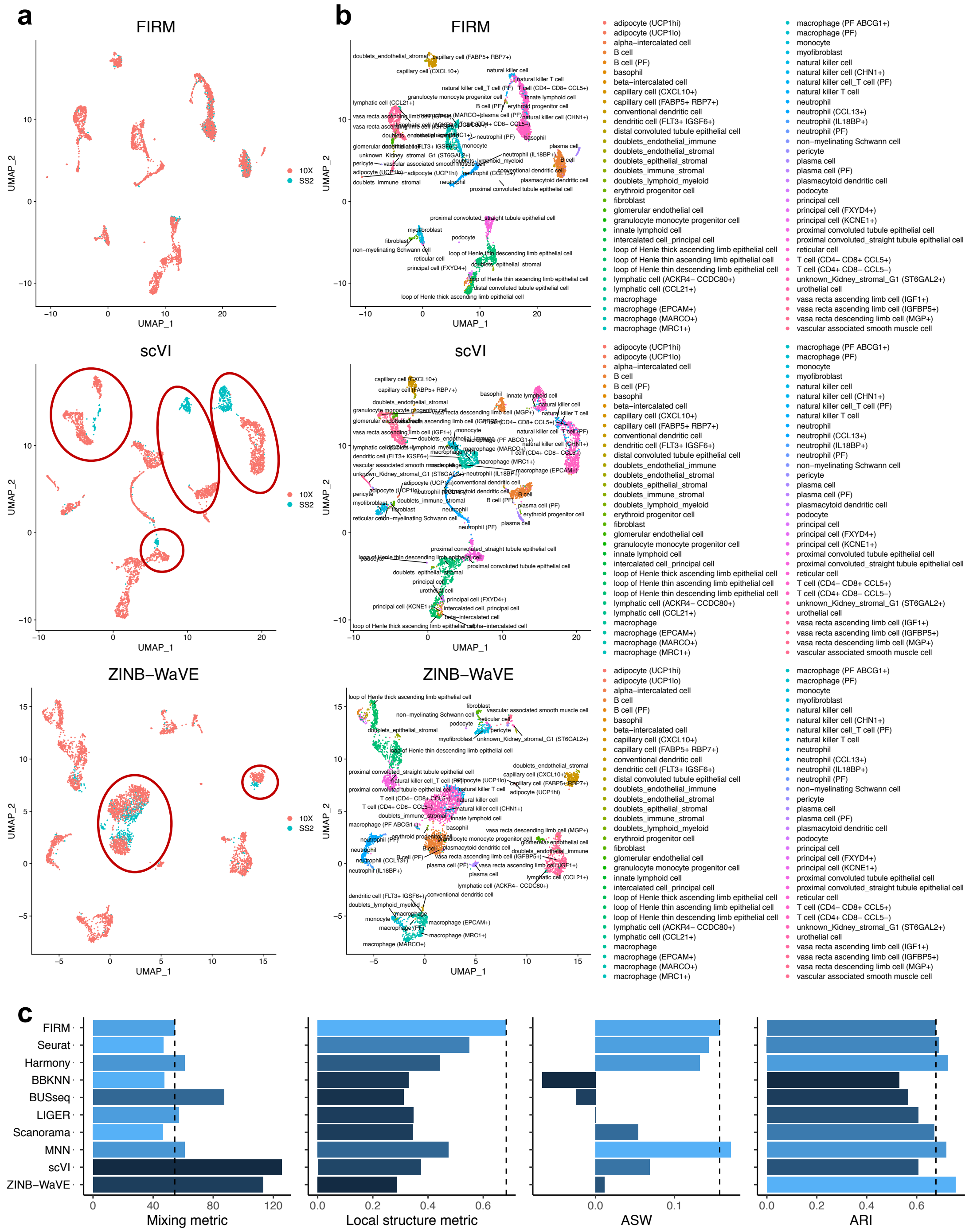

Supplementary Fig. 22 Comparison of integration methods based on the kidney scRNA-seq datasets generated by SS2 and 10X from lemur 2 in Tabula Microcebus. a, **b**, UMAP plots of the integrated scRNA-seq dataset colored by platform (**a**) and by cell type (**b**) using FIRM, scVI and ZINB-WaVE. The red circles highlight the problems of the integration results given by these methods. **c**, Metrics (mixing metric, local structure metric, ASW and ARI) for evaluating performance across the ten methods. The color (from light to dark) represents the performance (from the best to the worst). The dashed lines were set at the values for FIRM as reference lines.

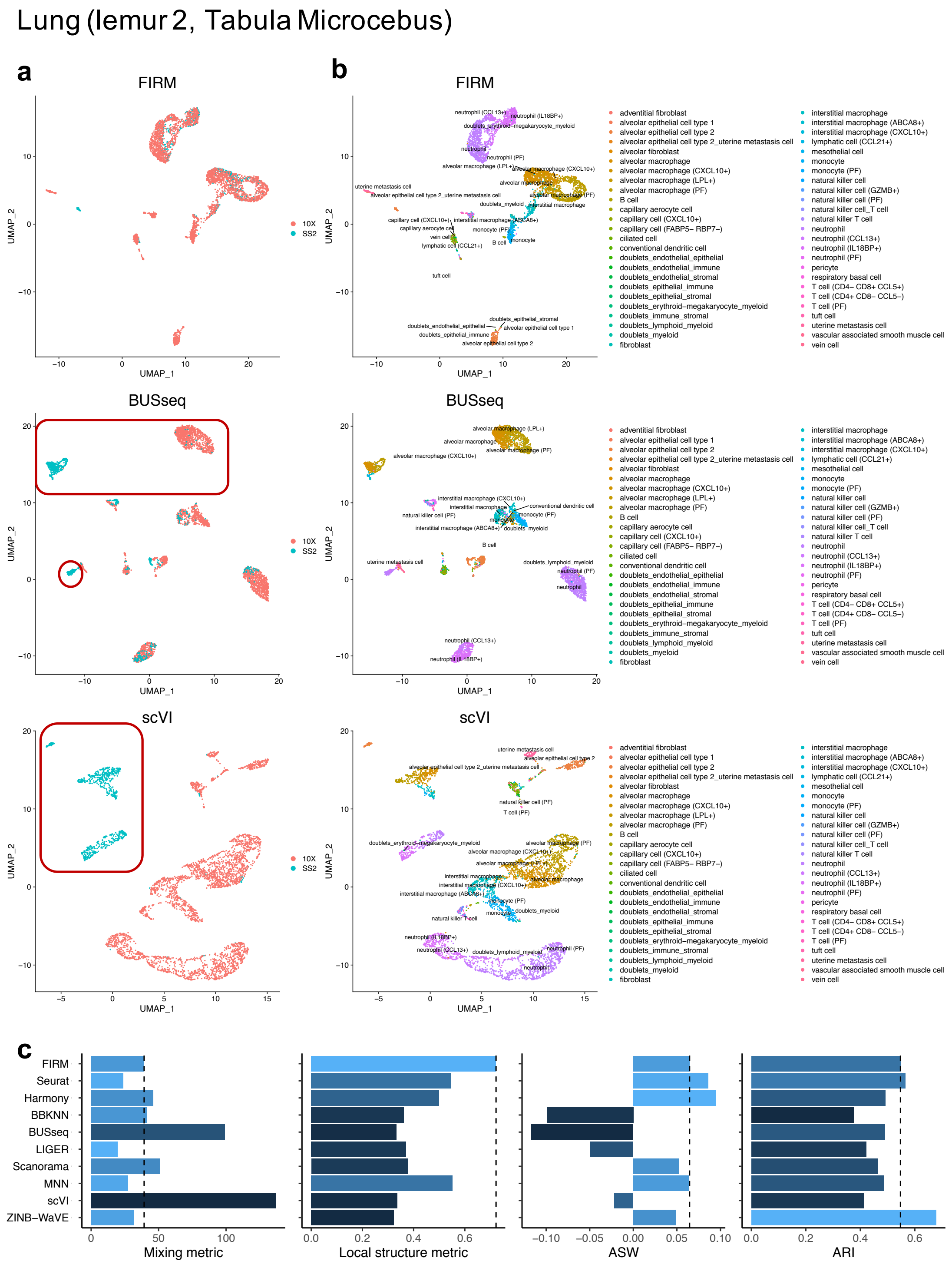

Supplementary Fig. 23 Comparison of integration methods based on the lung scRNA-seq datasets generated by SS2 and 10X from lemur 2 in Tabula Microcebus. a, **b**, UMAP plots of the integrated scRNA-seq dataset colored by platform (**a**) and by cell type (**b**) using FIRM, BUSseq and scVI. The red circles highlight the problems of the integration results given by these methods. **c**, Metrics (mixing metric, local structure metric, ASW and ARI) for evaluating performance across the ten methods. The color (from light to dark) represents the performance (from the best to the worst). The dashed lines were set at the values for FIRM as reference lines.

Spleen (lemur 2, Tabula Microcebus)

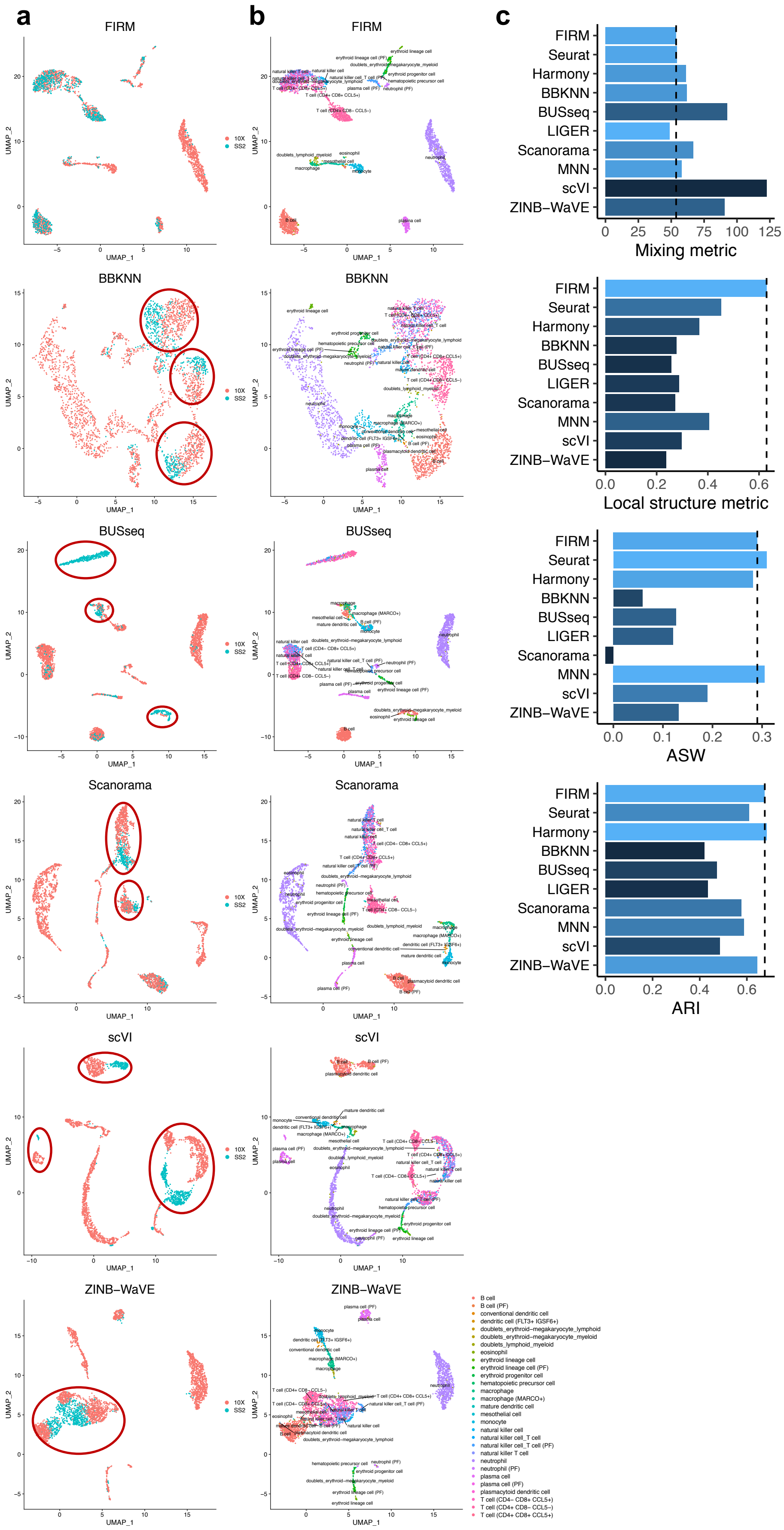

Supplementary Fig. 24 Comparison of integration methods based on the spleen scRNA-seq datasets generated by SS2 and 10X from lemur 2 in Tabula Microcebus. a, **b**, UMAP plots of the integrated scRNA-seq dataset colored by platform (**a**) and by cell type (**b**) using FIRM, BBKNN, BUSseq, Scanorama, scVI and ZINB-WaVE. The red circles highlight the problems of the integration results given by these methods. **c**, Metrics (mixing metric, local structure metric, ASW and ARI) for evaluating performance across the ten methods. The color (from light to dark) represents the performance (from the best to the worst). The dashed lines were set at the values for FIRM as reference lines.

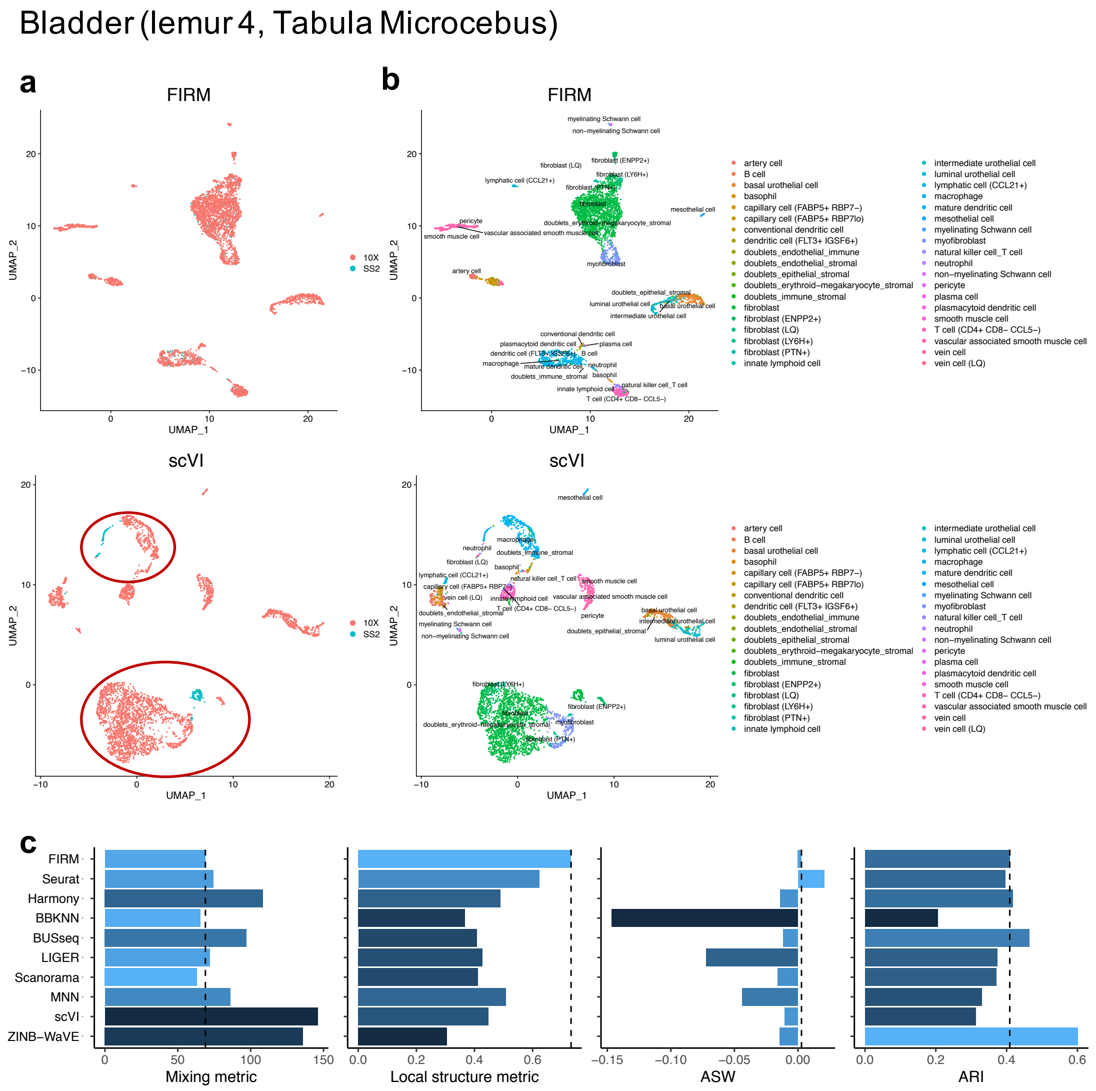

Supplementary Fig. 25 Comparison of integration methods based on the bladder scRNA-seq datasets generated by SS2 and 10X from lemur 4 in Tabula Microcebus. a, **b**, UMAP plots of the integrated scRNA-seq dataset colored by platform (**a**) and by cell type (**b**) using FIRM and scVI. The red circles highlight the problems of the integration results given by these methods. **c**, Metrics (mixing metric, local structure metric, ASW and ARI) for evaluating performance across the ten methods. The color (from light to dark) represents the performance (from the best to the worst). The dashed lines were set at the values for FIRM as reference lines.

Bone (lemur 4, Tabula Microcebus)

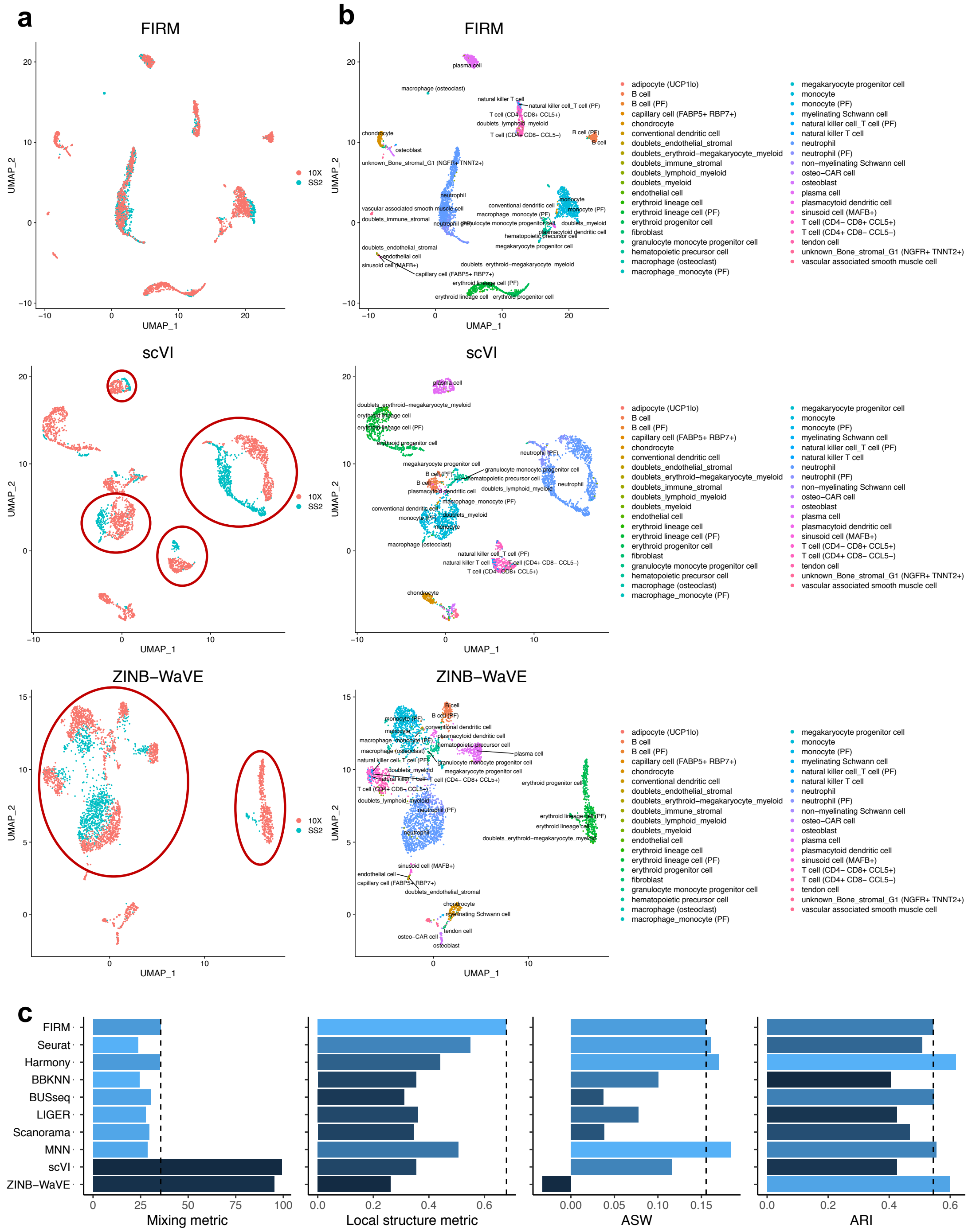

Supplementary Fig. 26 Comparison of integration methods based on the bone scRNA-seq datasets generated by SS2 and 10X from lemur 4 in Tabula Microcebus. a, **b**, UMAP plots of the integrated scRNA-seq dataset colored by platform (**a**) and by cell type (**b**) using FIRM, scVI and ZINB-WaVE. The red circles highlight the problems of the integration results given by these methods. **c**, Metrics (mixing metric, local structure metric, ASW and ARI) for evaluating performance across the ten methods. The color (from light to dark) represents the performance (from the best to the worst). The dashed lines were set at the values for FIRM as reference lines.

Bone marrow (lemur 4, Tabula Microcebus)

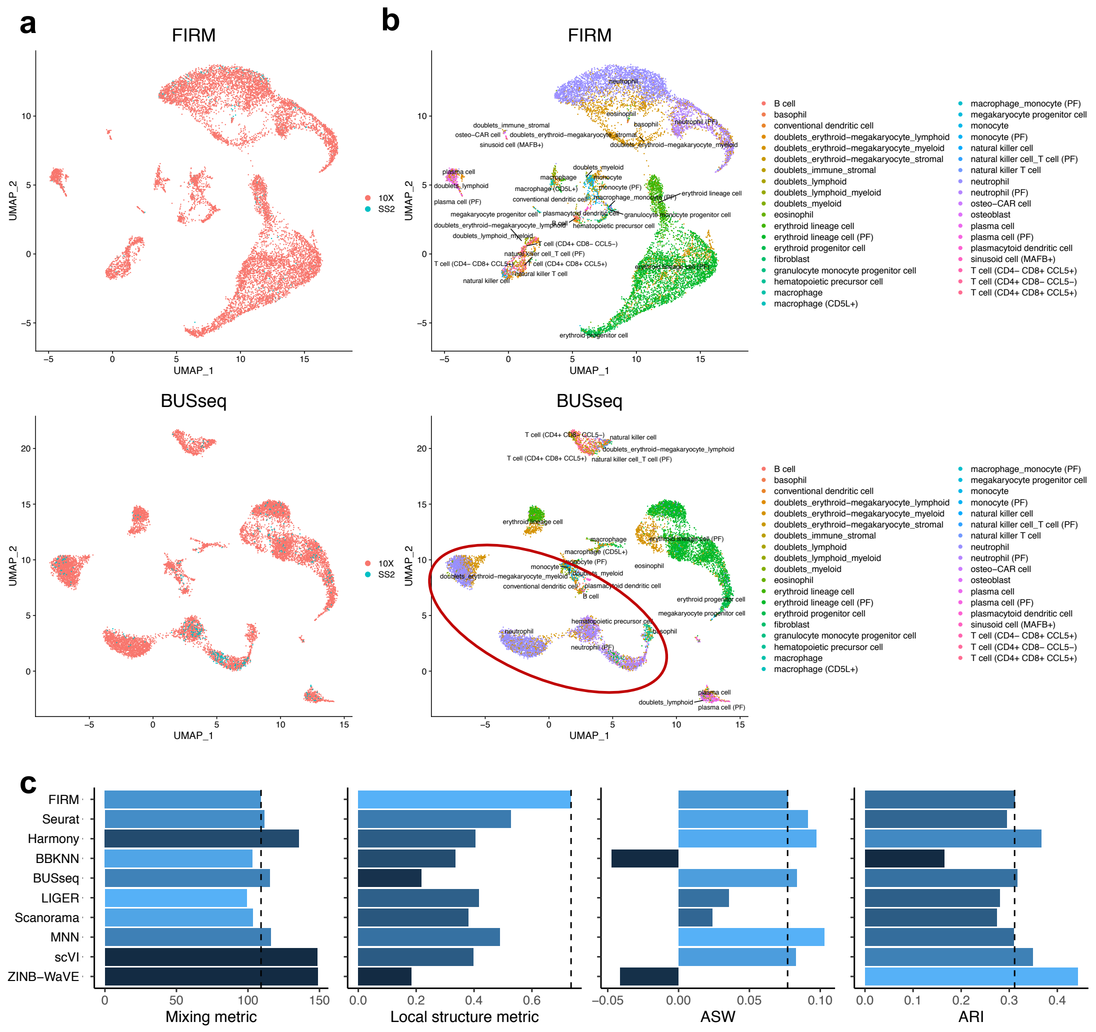

Supplementary Fig. 27 Comparison of integration methods based on the bone marrow scRNA-seq datasets generated by SS2 and 10X from lemur 4 in Tabula Microcebus. a, **b**, UMAP plots of the integrated scRNA-seq dataset colored by platform (**a**) and by cell type (**b**) using FIRM and BUSseq. The red circles highlight the problems of the integration results given by these methods. **c**, Metrics (mixing metric, local structure metric, ASW and ARI) for evaluating performance across the ten methods. The color (from light to dark) represents the performance (from the best to the worst). The dashed lines were set at the values for FIRM as reference lines.

Eye retina (lemur 4, Tabula Microcebus)

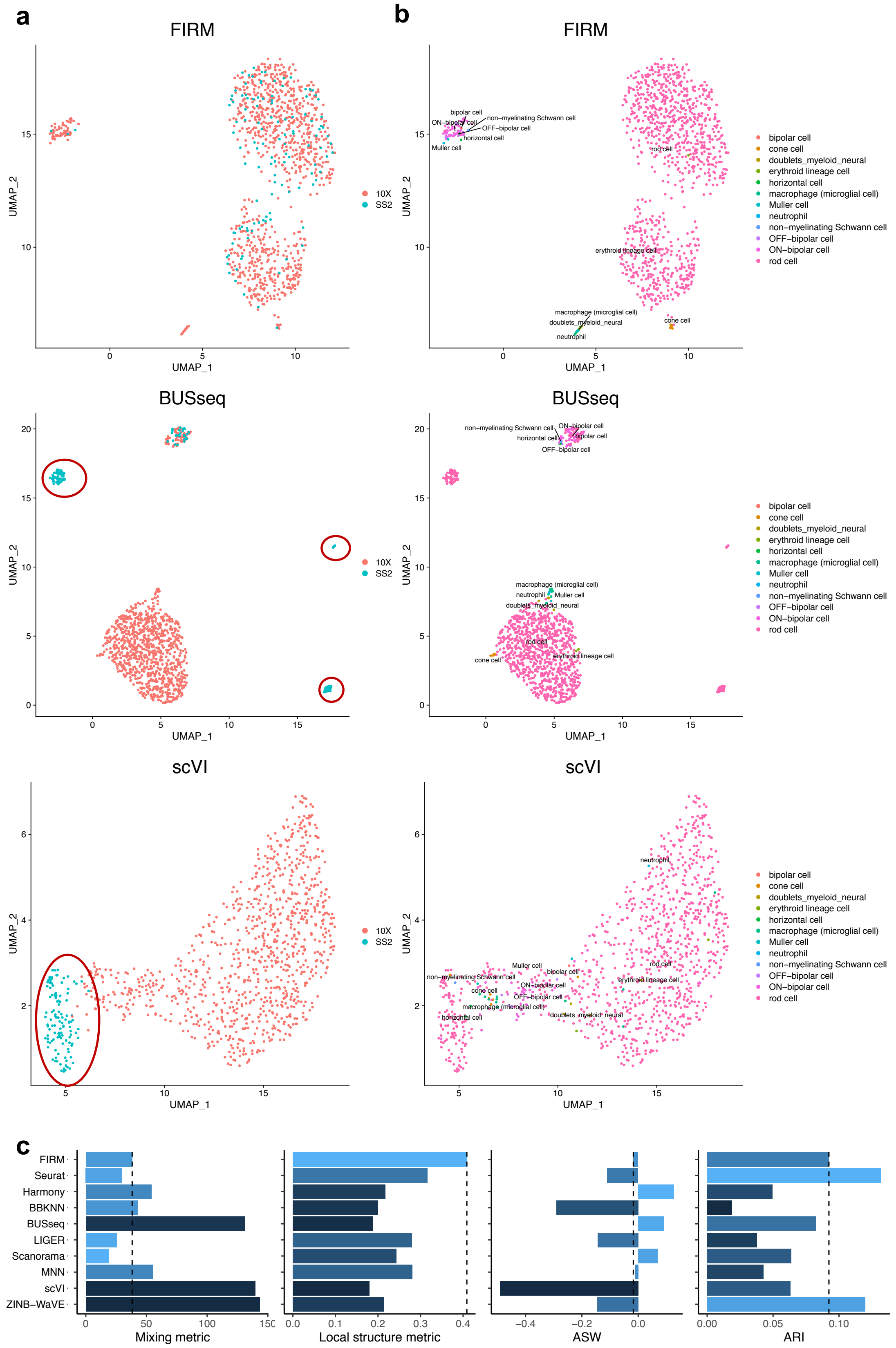

Supplementary Fig. 28 Comparison of integration methods based on the eye retina scRNA-seq datasets generated by SS2 and 10X from lemur 4 in Tabula Microcebus. a, b, UMAP plots of the integrated scRNA-seq

dataset colored by platform (**a**) and by cell type (**b**) using FIRM, BUSseq and scVI. The red circles highlight the problems of the integration results given by these methods. **c**, Metrics (mixing metric, local structure metric, ASW and ARI) for evaluating performance across the ten methods. The color (from light to dark) represents the performance (from the best to the worst). The dashed lines were set at the values for FIRM as reference lines.

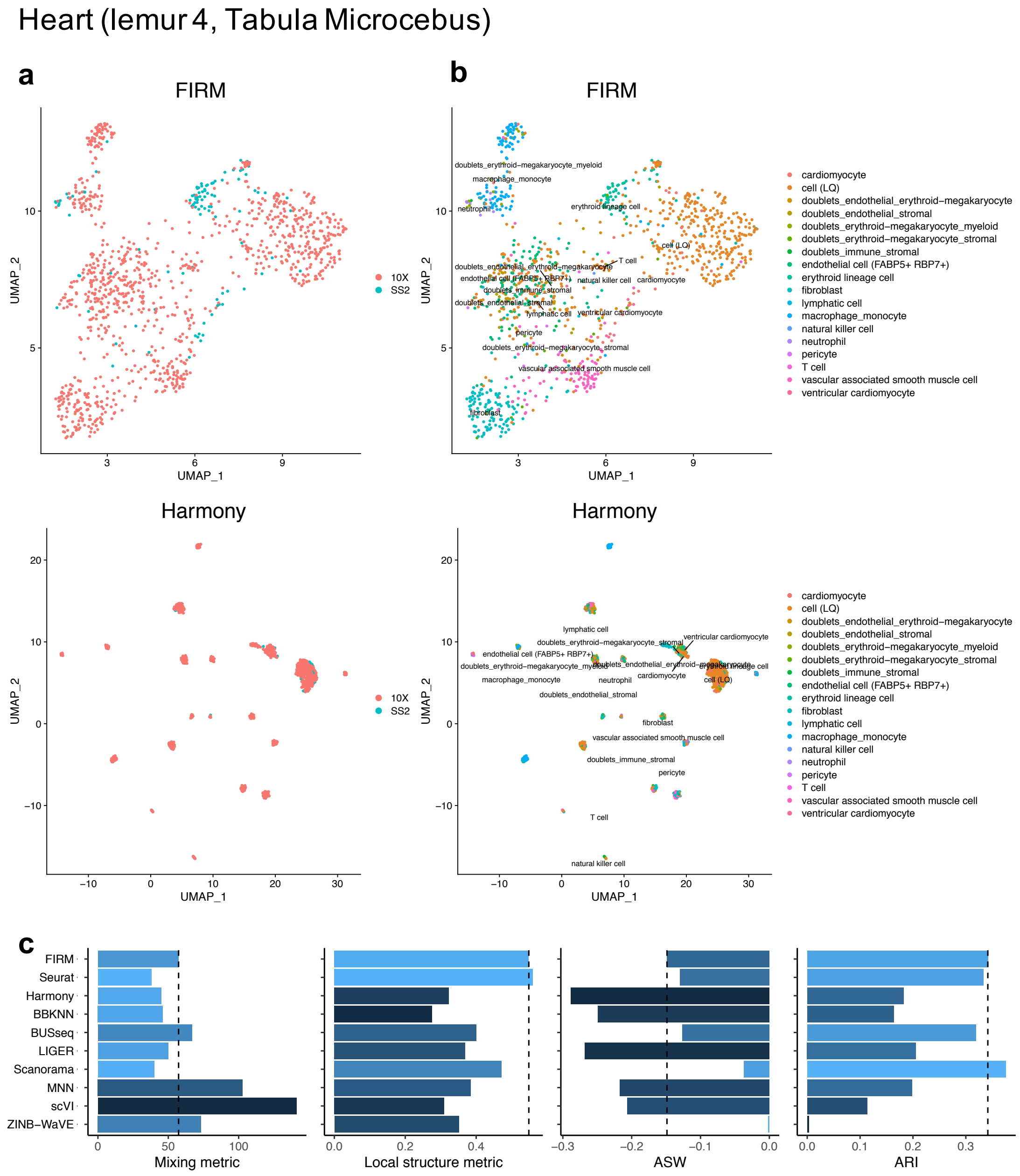

Supplementary Fig. 29 Comparison of integration methods based on the heart scRNA-seq datasets generated by SS2 and 10X from lemur 4 in Tabula Microcebus. a, **b**, UMAP plots of the integrated scRNA-seq dataset colored by platform (**a**) and by cell type (**b**) using FIRM and Harmony. **c**, Metrics (mixing metric, local structure metric, ASW and ARI) for evaluating performance across the ten methods. The color (from light to dark) represents the performance (from the best to the worst). The dashed lines were set at the values for FIRM as reference lines.

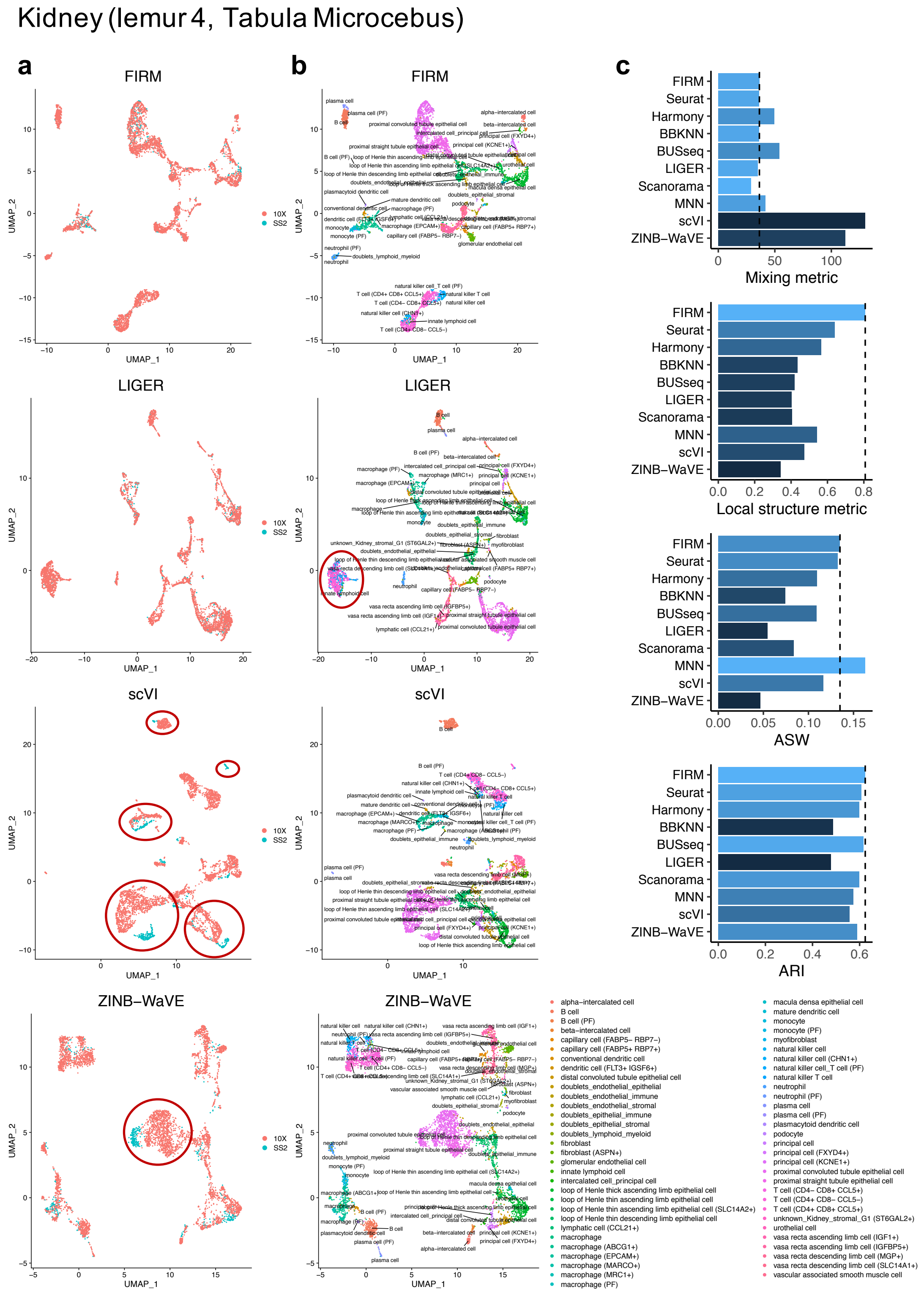

Supplementary Fig. 30 Comparison of integration methods based on the kidney scRNA-seq datasets generated by SS2 and 10X from lemur 4 in Tabula Microcebus. a, **b**, UMAP plots of the integrated scRNA-seq dataset colored by platform (**a**) and by cell type (**b**) using FIRM, LIGER, scVI and ZINB-WaVE. The red circles highlight the problems of the integration results given by these methods. **c**, Metrics (mixing metric, local structure metric, ASW and ARI) for evaluating performance across the ten methods. The color (from light to dark) represents the performance (from the best to the worst). The dashed lines were set at the values for FIRM as reference lines.

Liver (lemur 4, Tabula Microcebus)

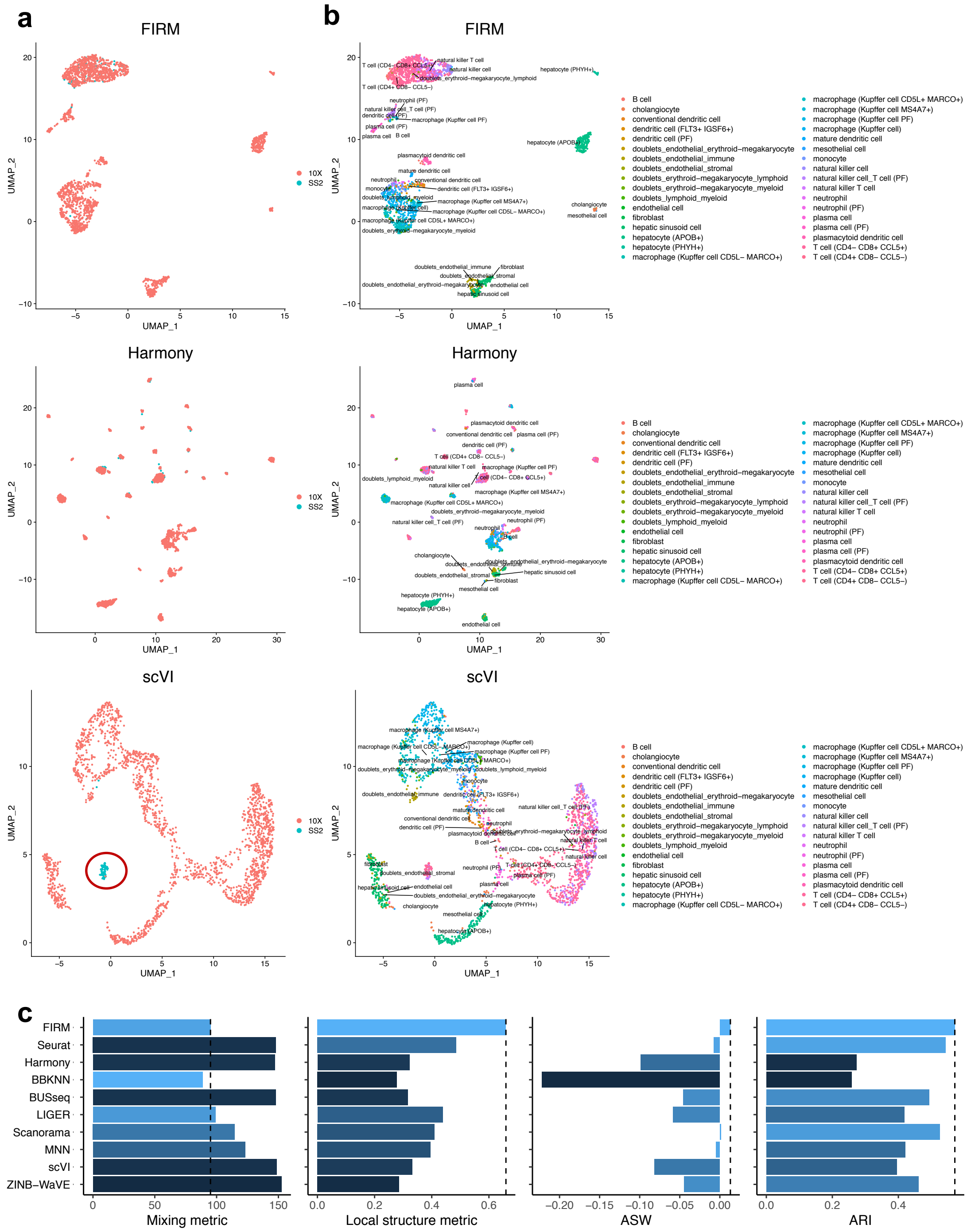

Supplementary Fig. 31 Comparison of integration methods based on the liver scRNA-seq datasets generated by SS2 and 10X from lemur 4 in Tabula Microcebus. a, **b**, UMAP plots of the integrated scRNA-seq dataset colored by platform (**a**) and by cell type (**b**) using FIRM, Harmony and scVI. The red circles highlight the problems of the integration results given by these methods. **c**, Metrics (mixing metric, local structure metric, ASW and ARI) for evaluating performance across the ten methods. The color (from light to dark) represents the performance (from the best to the worst). The dashed lines were set at the values for FIRM as reference lines.

Pancreas (lemur 4, Tabula Microcebus)

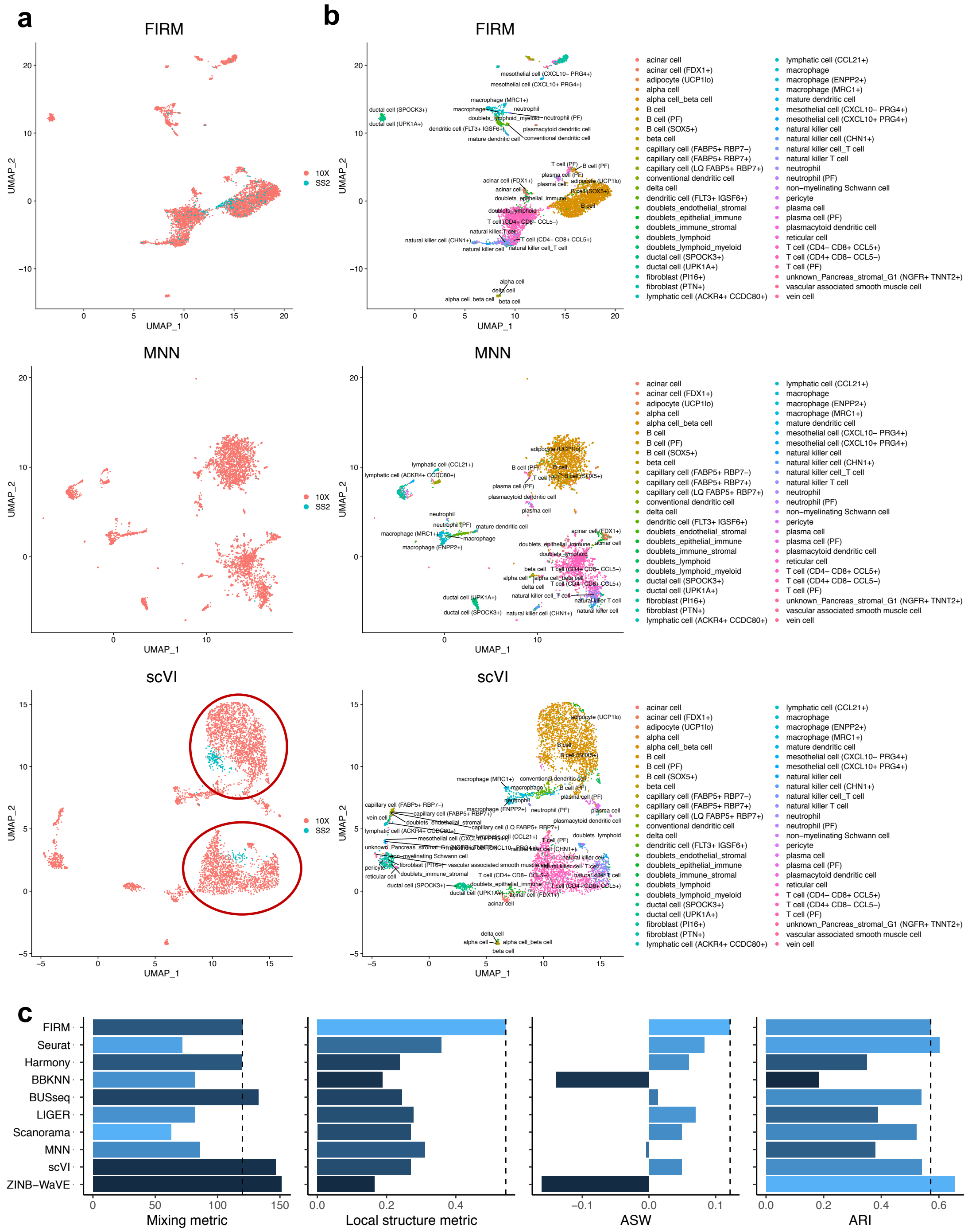

Supplementary Fig. 32 Comparison of integration methods based on the pancreas scRNA-seq datasets generated by SS2 and 10X from lemur 4 in Tabula Microcebus. a, **b**, UMAP plots of the integrated scRNA-seq dataset colored by platform (**a**) and by cell type (**b**) using FIRM, MNN and scVI. The red circles highlight the problems of the integration results given by these methods. **c**, Metrics (mixing metric, local structure metric, ASW and ARI) for evaluating performance across the ten methods. The color (from light to dark) represents the performance (from the best to the worst). The dashed lines were set at the values for FIRM as reference lines.

Small intestine (lemur 4, Tabula Microcebus)

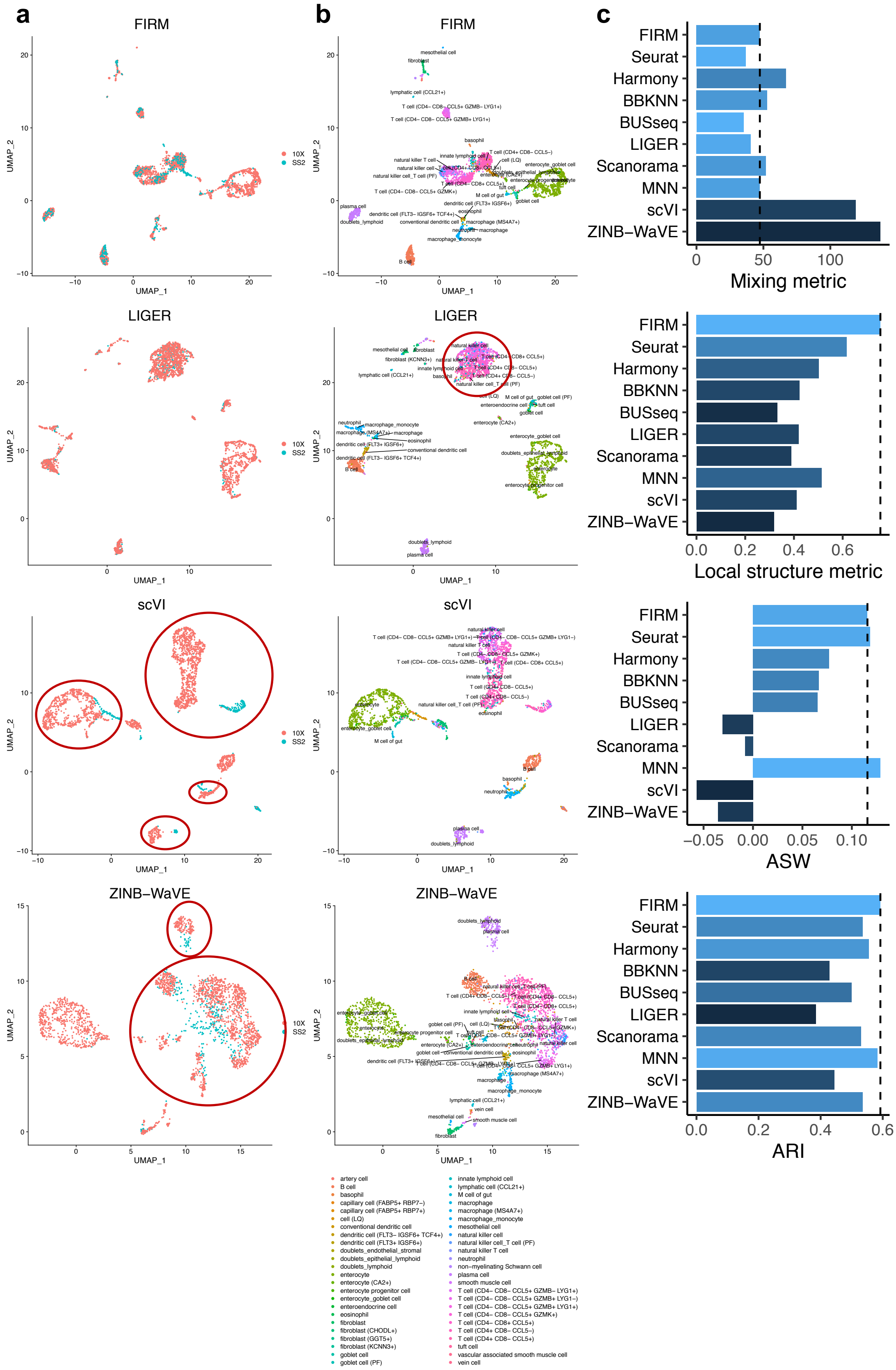

Supplementary Fig. 33 Comparison of integration methods based on the small intestine scRNA-seq datasets

generated by SS2 and 10X from lemur 4 in Tabula Microcebus. a, **b**, UMAP plots of the integrated scRNA-seq dataset colored by platform (**a**) and by cell type (**b**) using FIRM, LIGER, scVI and ZINB-WaVE. The red circles highlight the problems of the integration results given by these methods. **c**, Metrics (mixing metric, local structure metric, ASW and ARI) for evaluating performance across the ten methods. The color (from light to dark) represents the performance (from the best to the worst). The dashed lines were set at the values for FIRM as reference lines.

Spleen (lemur 4, Tabula Microcebus)

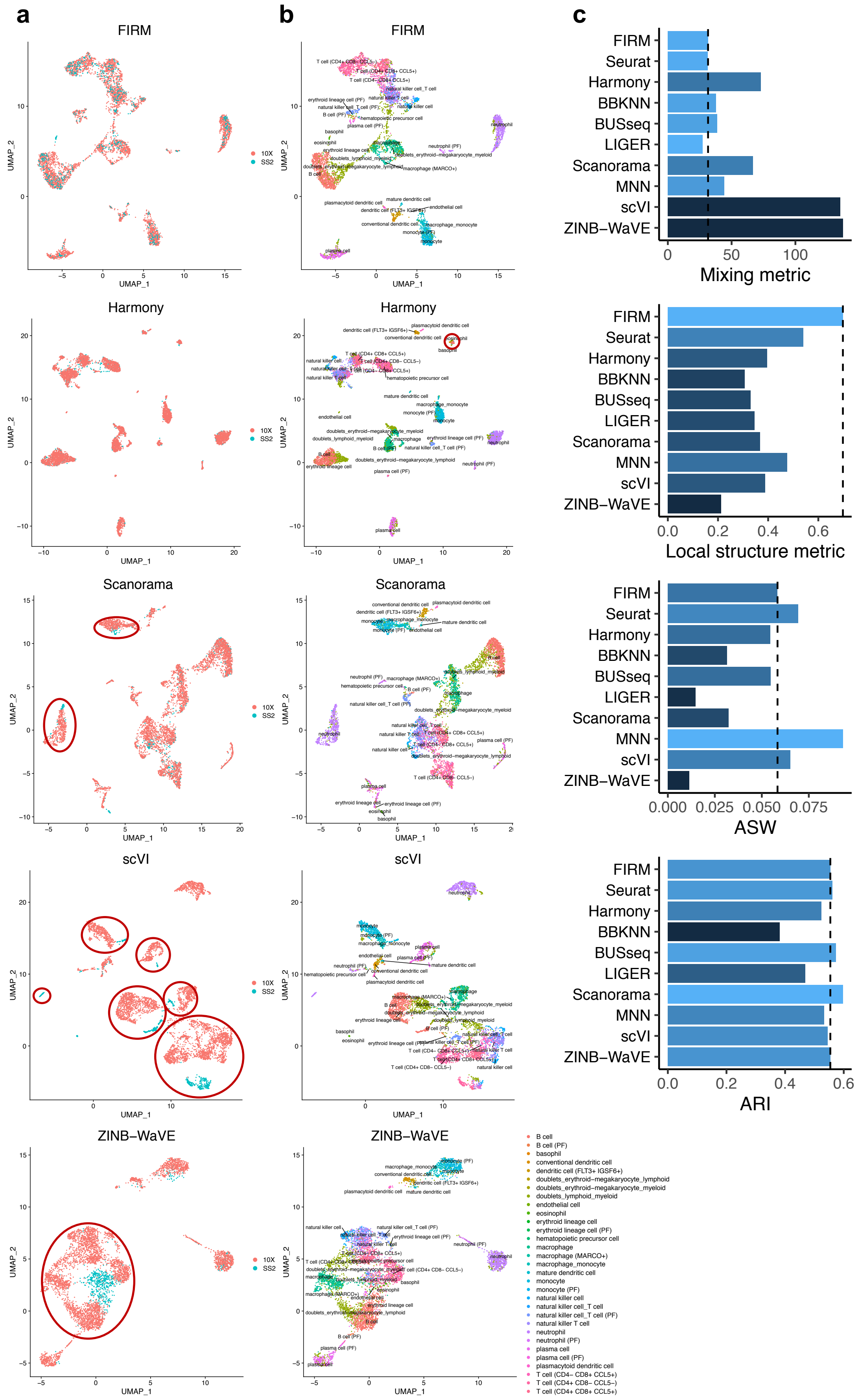

Supplementary Fig. 34 Comparison of integration methods based on the spleen scRNA-seq datasets generated by SS2 and 10X from lemur 4 in Tabula Microcebus. a, **b**, UMAP plots of the integrated scRNA-seq dataset colored by platform (**a**) and by cell type (**b**) using FIRM, Harmony, Scanorama, scVI and ZINB-WaVE. The red circles highlight the problems of the integration results given by these methods. **c**, Metrics (mixing metric, local structure metric, ASW and ARI) for evaluating performance across the ten methods. The color (from light to dark) represents the performance (from the best to the worst). The dashed lines were set at the values for FIRM as reference lines.

Testes (lemur 4, Tabula Microcebus)

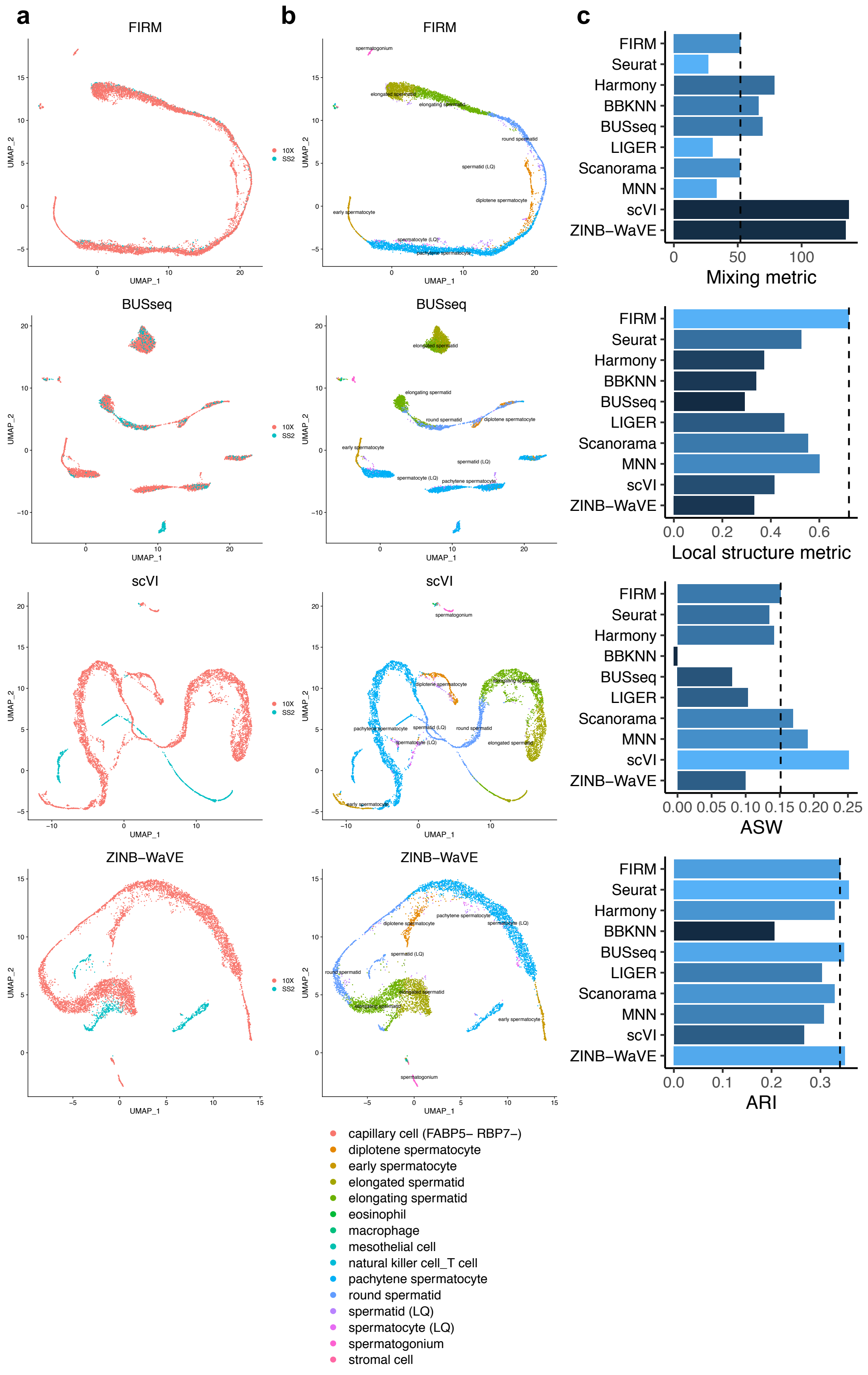

Supplementary Fig. 35 Comparison of integration methods based on the testes scRNA-seq datasets generated by SS2 and 10X from lemur 4 in Tabula Microcebus. a, **b**, UMAP plots of the integrated scRNA-seq dataset colored by platform (**a**) and by cell type (**b**) using FIRM, BUSseq, scVI and ZINB-WaVE. **c**, Metrics (mixing metric, local structure metric, ASW and ARI) for evaluating performance across the ten methods. The color (from light to dark) represents the performance (from the best to the worst). The dashed lines were set at the values for FIRM as reference lines.

Tongue (lemur 4, Tabula Microcebus)

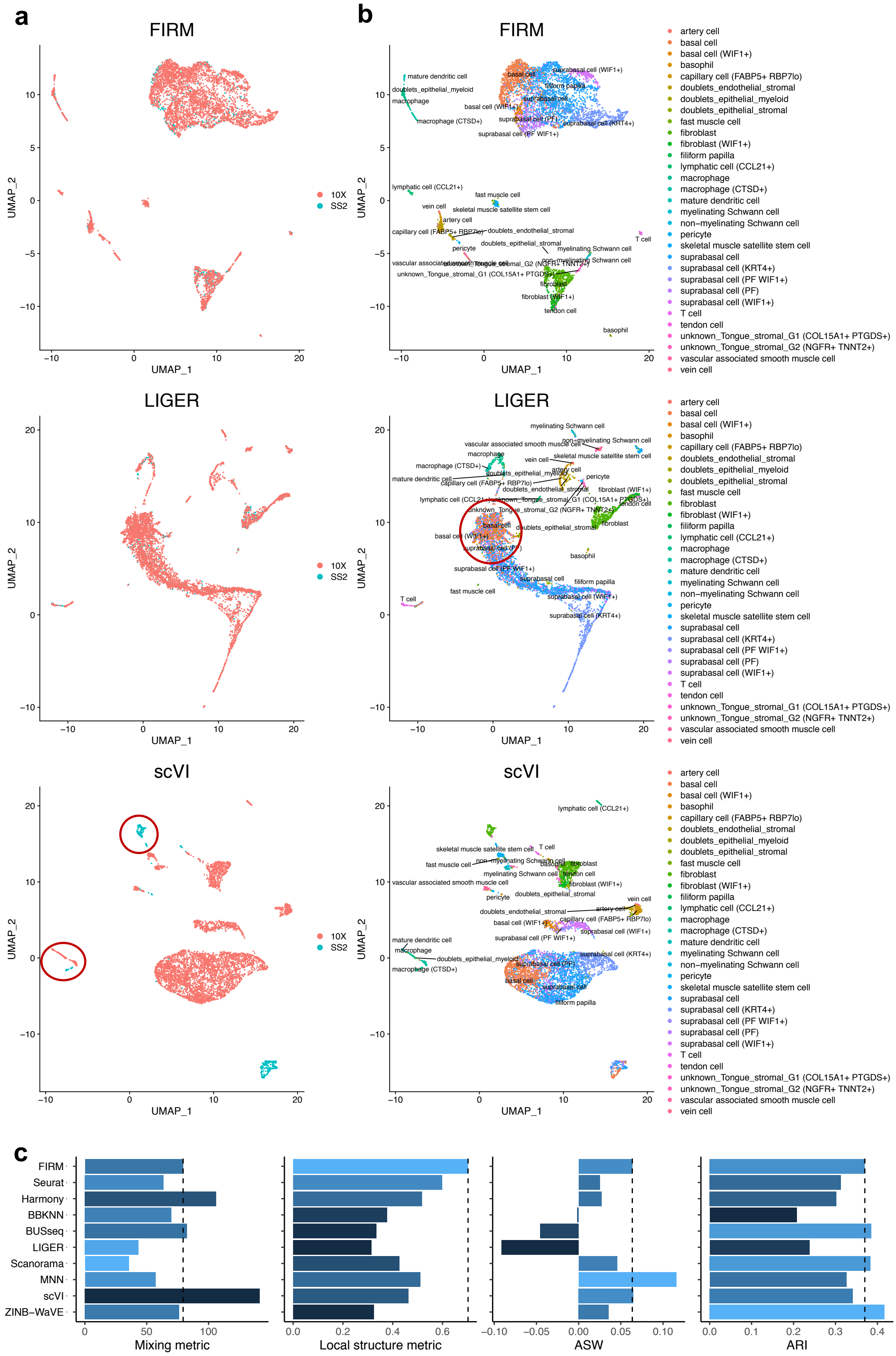

Supplementary Fig. 36 Comparison of integration methods based on the tongue scRNA-seq datasets generated by SS2 and 10X from lemur 4 in Tabula Microcebus. a, **b**, UMAP plots of the integrated scRNA-seq dataset

colored by platform (**a**) and by cell type (**b**) using FIRM, LIGER and scVI. The red circles highlight the problems of the integration results given by these methods. **c**, Metrics (mixing metric, local structure metric, ASW and ARI) for evaluating performance across the ten methods. The color (from light to dark) represents the performance (from the best to the worst). The dashed lines were set at the values for FIRM as reference lines.

Human Lung Cell Atlas

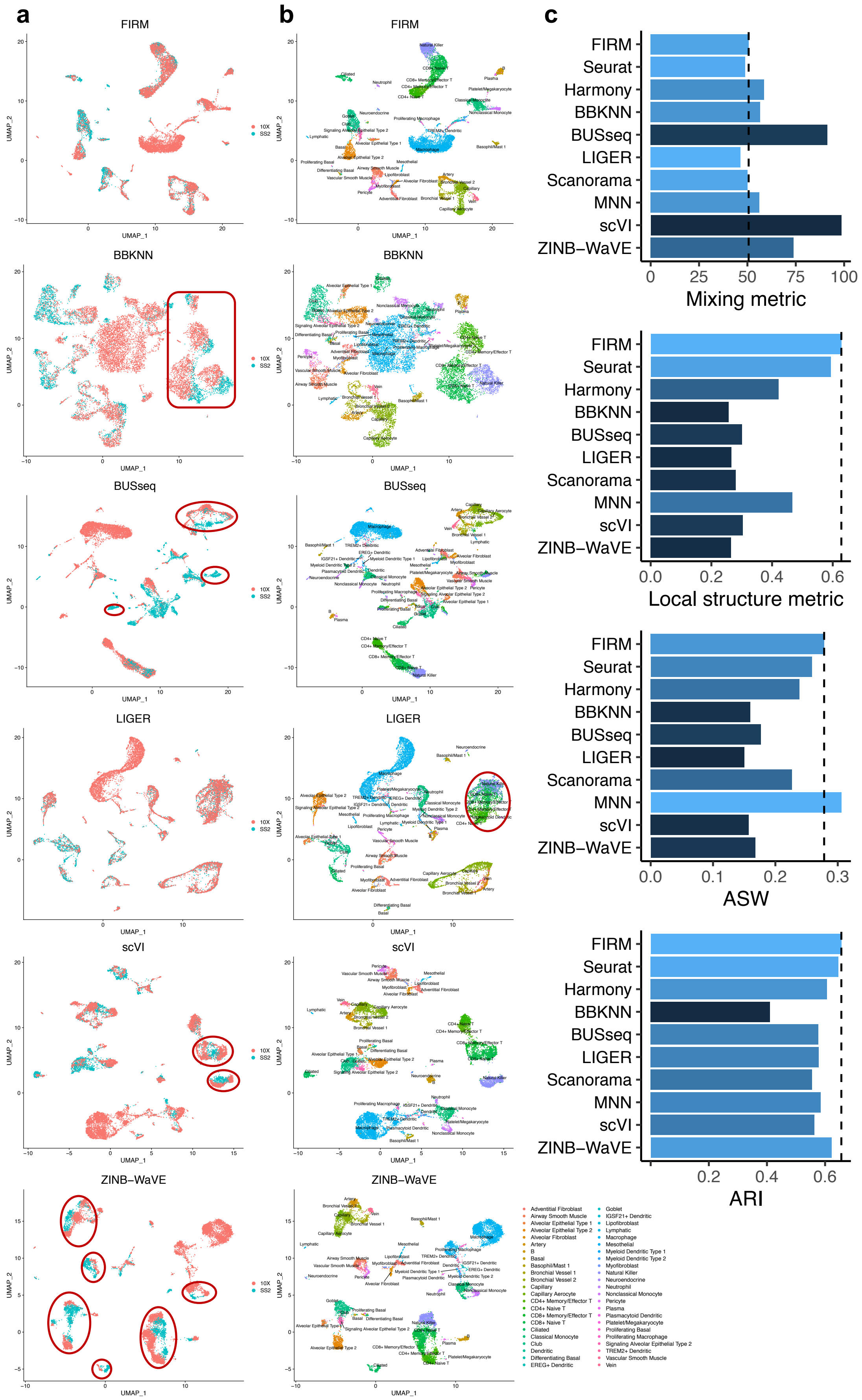

Supplementary Fig. 37 Comparison of integration methods based on the scRNA-seq datasets generated by SS2 and 10X from Human Lung Cell Atlas [4]. a, **b**, UMAP plots of the integrated scRNA-seq dataset colored by platform (**a**) and by cell type (**b**) using FIRM, BBKNN, BUSseq, LIGER, scVI and ZINB-WaVE. The red circles highlight the problems of the integration results given by these methods. **c**, Metrics (mixing metric, local structure metric, ASW and ARI) for evaluating performance across the ten methods. The color (from light to dark) represents the performance (from the best to the worst). The dashed lines were set at the values for FIRM as reference lines.

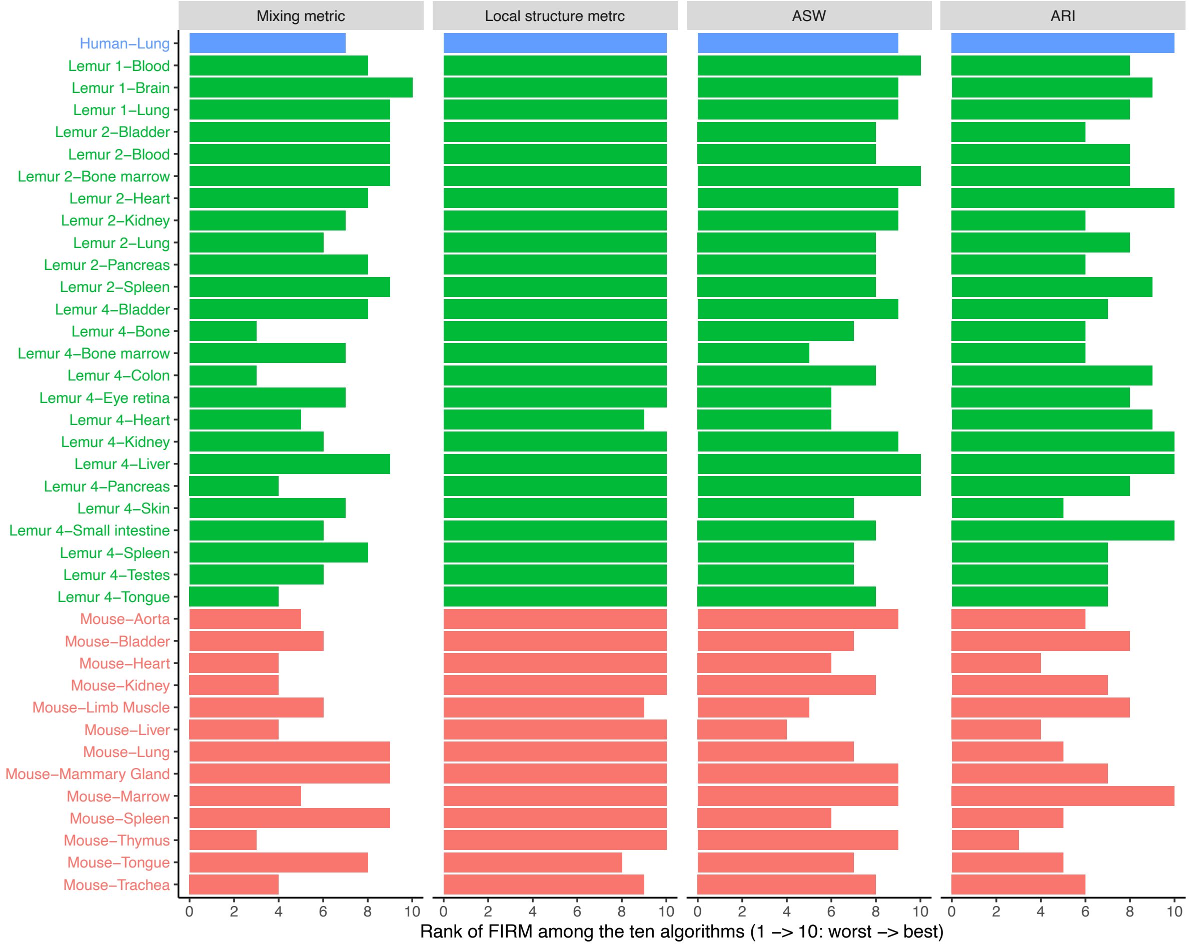

Supplementary Fig. 38 The ranks of FIRM among ten algorithms in four metrics for integration of scRNA-seq datasets. The datasets include 13 pairs of SS2 and 10X scRNA-seq datasets from Tabula Muris, 25 pairs from Tabula Microcebus, and one pair in Human Lung Cell Atlas. Four metrics include mixing metric, local structure metric, ASW and ARI.

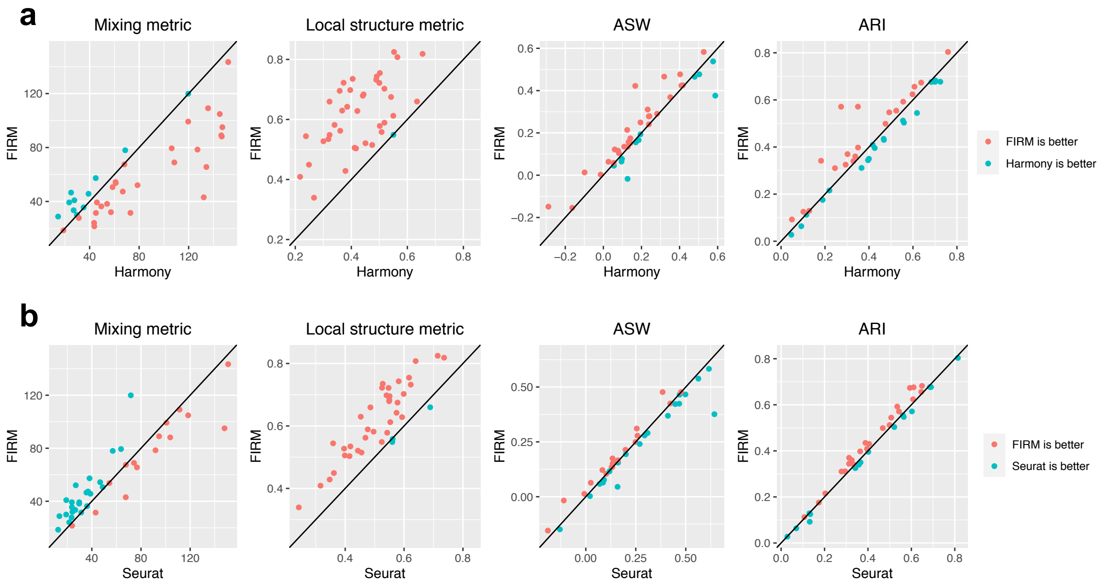

Supplementary Fig. 39 Comparisons between FIRM and Harmony (a) and Seurat (b) in four metrics for integration of scRNA-seq datasets. The datasets include 13 pairs of SS2 and 10X scRNA-seq datasets from Tabula Muris, 25 pairs from Tabula Microcebus, and one pair in Human Lung Cell Atlas. Four metrics include mixing metric, local structure metric, ASW and ARI.

Marker expression patterns for the Tabula Microcebus lemur 4 testes data

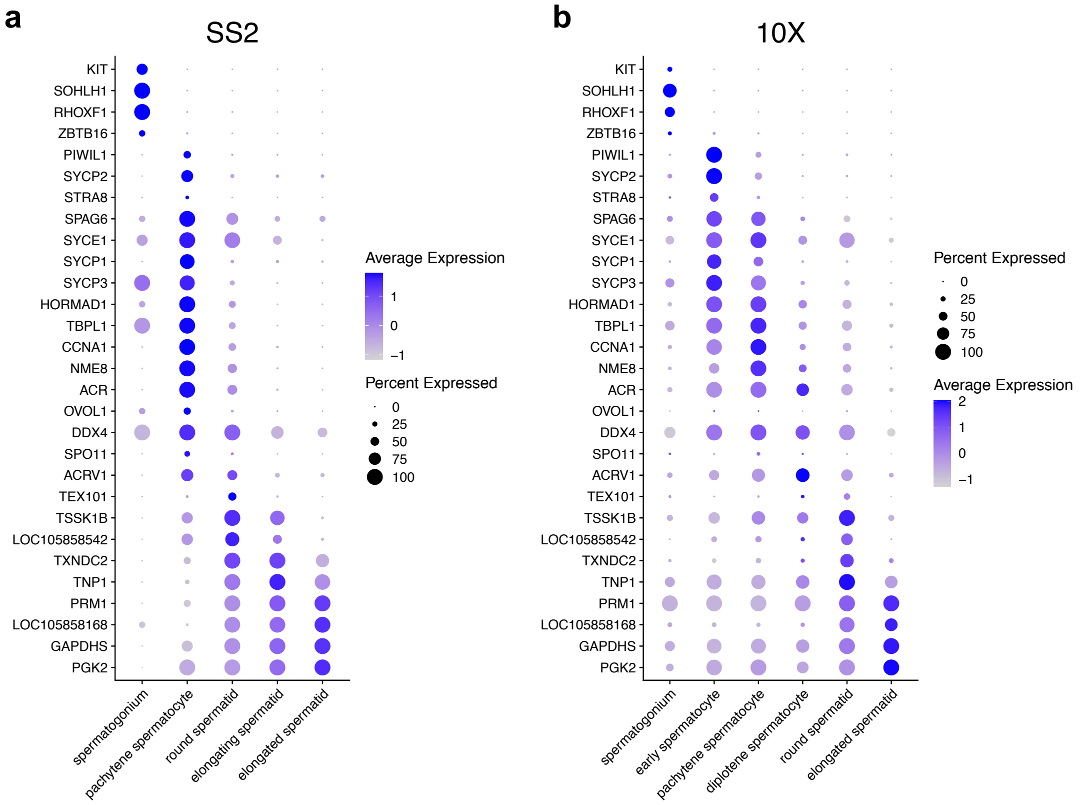

Supplementary Fig. 40 Dot plot showing relative expression of marker genes in SS2 dataset (**a**) and 10X dataset (**b**), using the cluster based on integrated dataset, in the Tabula Microcebus lemur 4 testes scRNA-seq datasets.

Integration of all the SS2 datasets in Tabula Microcebus

We used FIRM to integrate 29 SS2 datasets across three individuals and 20 tissues, which contain a total of 12,329 cells. We first integrated the datasets from the same tissue but different individuals and obtained the integrated data for each tissue. Then we further integrated the integrated datasets for different tissues, two datasets at a time. After each integration, the resulting integrated data is considered as a new dataset and is then further integrated with another dataset.

Integration for Tabula Muris

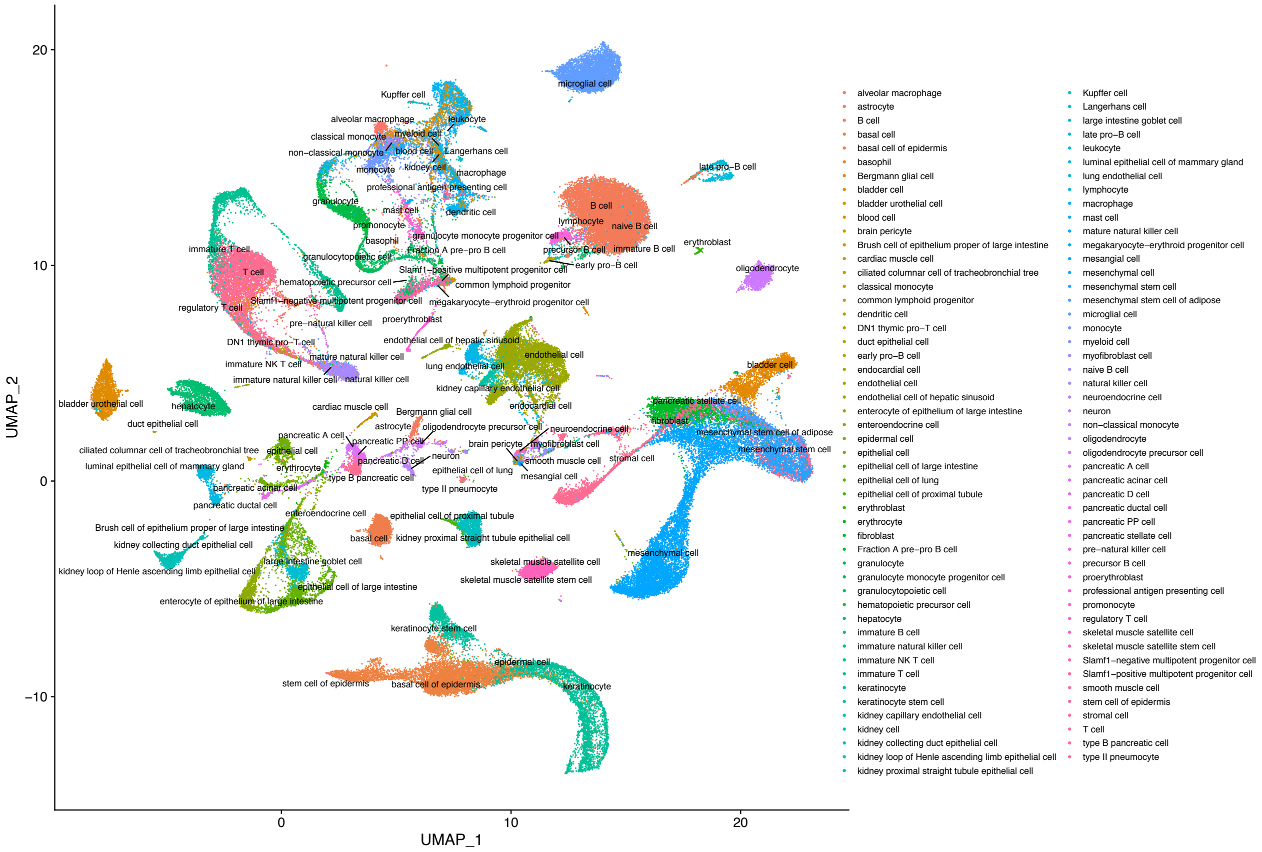

Supplementary Fig. 41 UMAP plot of the integrated scRNA-seq dataset for the whole SS2 dataset and 10X dataset of the entire organism in Tabula Muris colored by cell type using FIRM.

Computational time

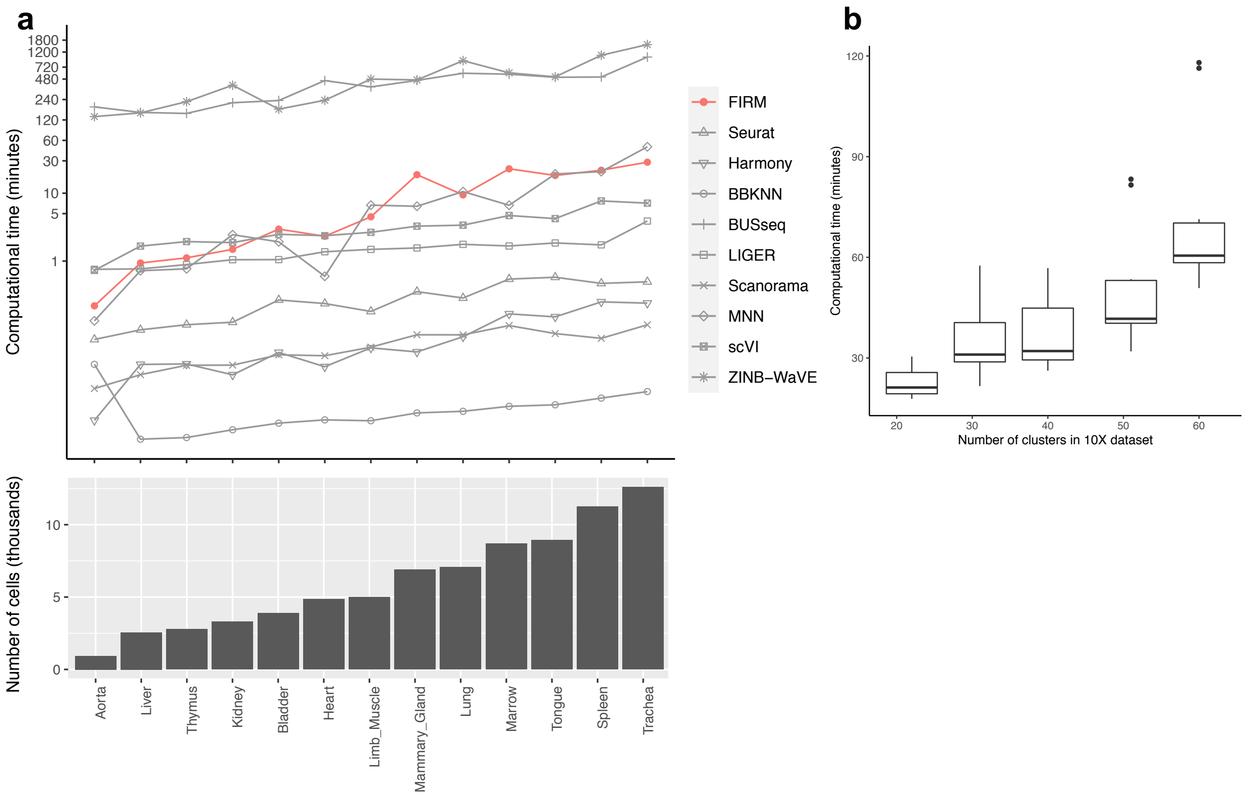

Supplementary Fig. 42 The computational time of FIRM and benchmarked methods. a, The computational time of FIRM and benchmarked methods for the integration of SS2 and 10X datasets for each tissue in Tabula Muris with different numbers of cells. The results of FIRM and BUSseq were evaluated with 30 CPU cores. **b**, The computational time of FIRM under one resolution pair with various numbers of clusters for integrating atlas-level datasets across platforms in Tabula Muris.

Comparison of PCA and kernel PCA for dimension reduction

We compared the performance of PCA and kernel PCA using the mammary gland dataset consisting of both SS2 and 10X data from Tabula Muris. For each dataset, we followed the data preprocessing procedure described in the “Materials and methods” section in the main text, which included normalization, scaling and feature selection. Then we concatenated the scaled data with the overlapped highly variable genes in SS2 and 10X dataset to obtain the integrated data. We conducted dimension reduction with 15 PCs based on the integrated data using PCA and kernel PCA, respectively. For kernel PCA, we tried the polynomial kernel function with different degrees, and the radial basis kernel function (RBF) with different scaling parameters (sigma). The UMAP visualizations are shown in Supplementary Fig. 43-45, and the metrics for evaluating the integration performance and

computational time are shown in Supplementary Fig. 46.

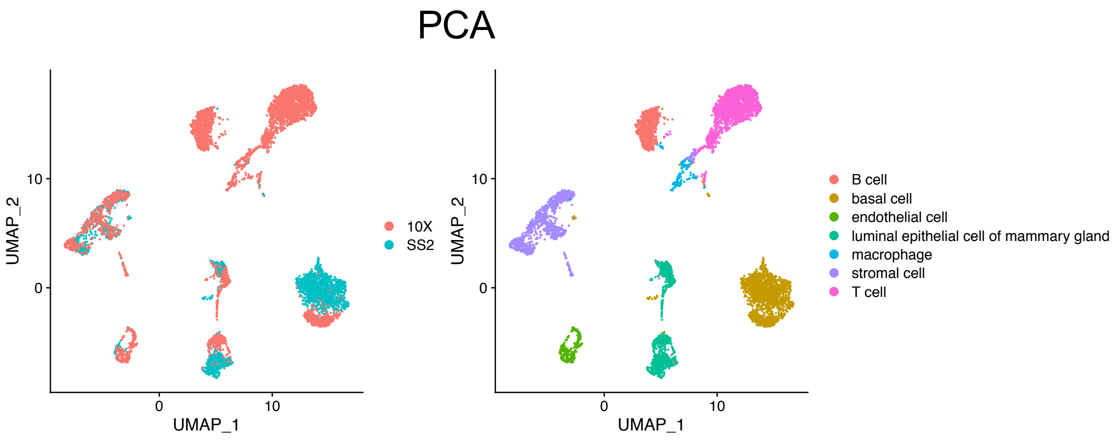

Supplementary Fig. 43 UMAP plots of the integrated scRNA-seq dataset for mammary gland from Tabula Muris using PCA as the dimension reduction method. The plots were colored by platform (left) and by cell type (right).

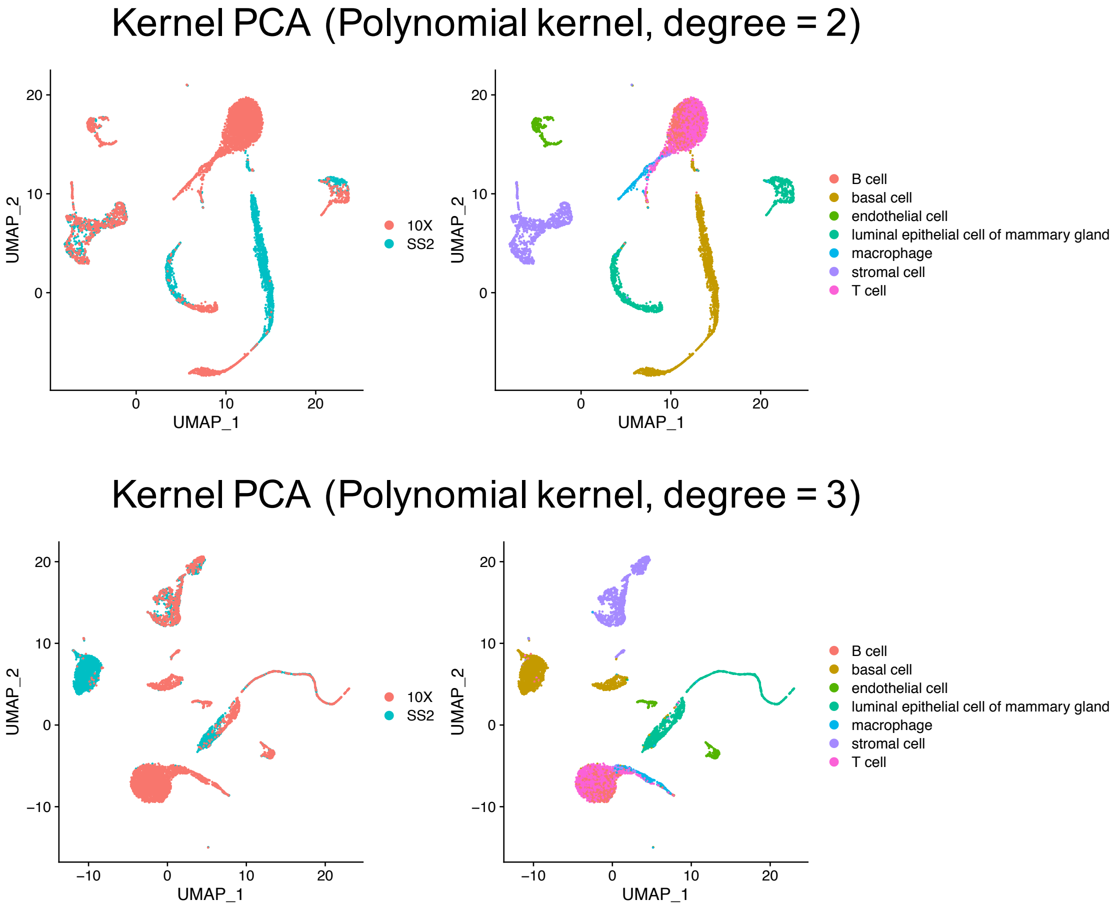

Supplementary Fig. 44 UMAP plots of the integrated scRNA-seq dataset for mammary gland from Tabula Muris using kernel PCA (the polynomial kernel function with different degrees) as the dimension reduction method. The plots were colored by platform (left) and by cell type (right).

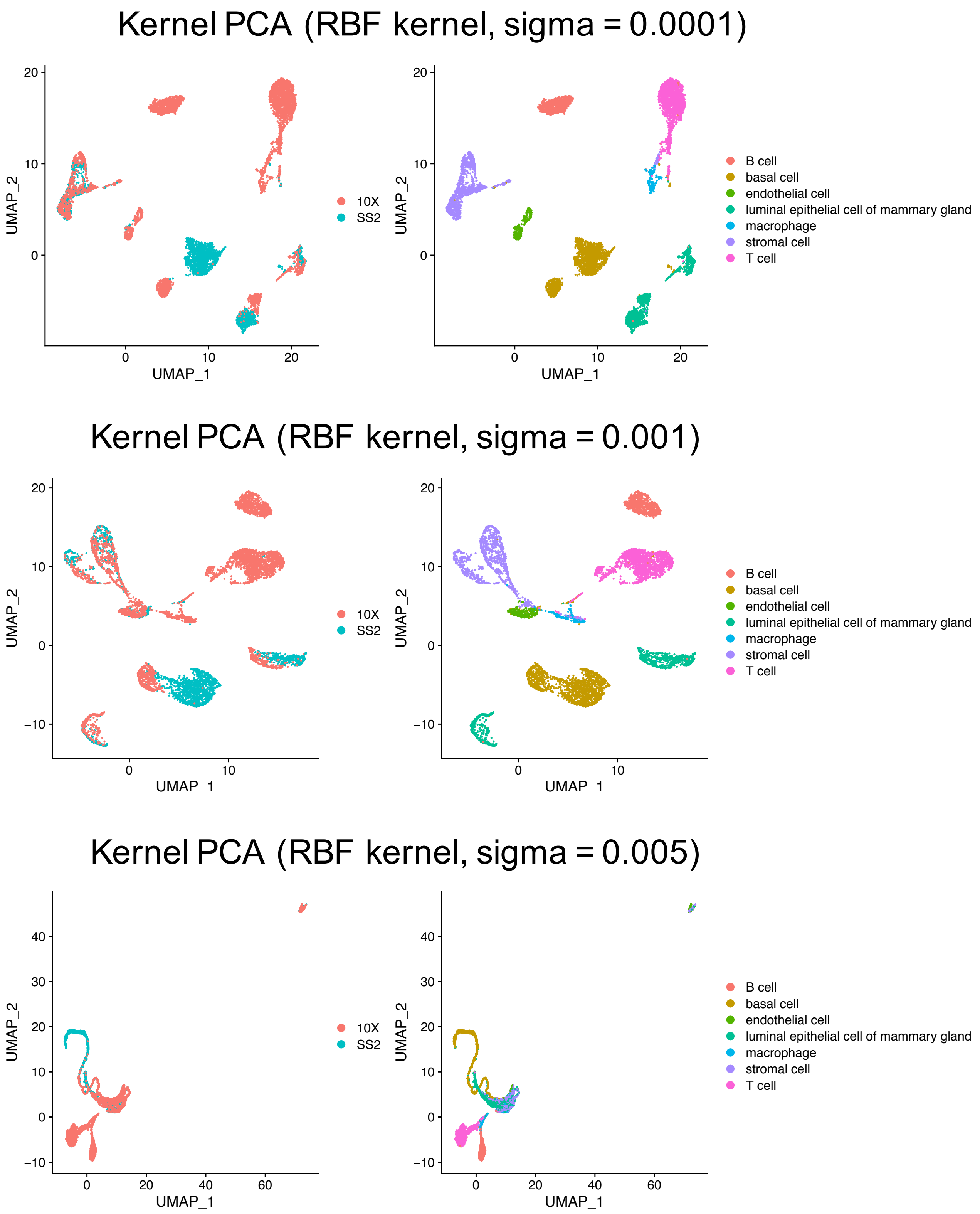

Supplementary Fig. 45 UMAP plots of the integrated scRNA-seq dataset for mammary gland from Tabula Muris using kernel PCA (the radial basis kernel function with different scale parameters: sigmas) as the dimension reduction method. The plots were colored by platform (left) and by cell type (right).

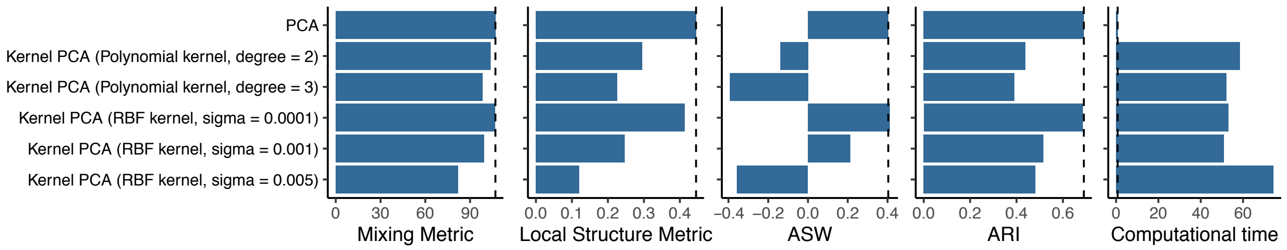

Supplementary Fig. 46 The metrics (mixing metric, local structure metric, ASW and ARI) for evaluating the integration performance and the computational time (in seconds) of the mammary gland data from Tabula Muris using different dimension reduction methods. The dashed lines were set at the values using PCA as reference lines.

We found that kernel PCA is quite sensitive to the parameters in the kernel function. Kernel PCA using RBF with sigma = 0.0001 has similar performance to PCA in terms of UMAP visualization and evaluation metrics. However, as the sigma increases (e.g., sigma = 0.001 and sigma = 0.005), kernel PCA using the RBF faces difficulties in separation different cell types, resulting in low ASW and ARI. Kernel PCA using the polynomial kernel function on this data shows a similar effect.

PCA is the simplest dimensionality reduction algorithm, and has a great advantage in computational efficiency. Compared with PCA, kernel PCA can be quite time-consuming. As shown in the rightmost panel of Supplementary Fig. 46, Kernel PCA took about 1 minute for the mammary gland data with 6886 cells, whereas PCA took less than 1 second.

Similar results are shown in Supplementary Fig. 47-50 for the limb muscle dataset generated using SS2 and 10X from Tabula Muris.

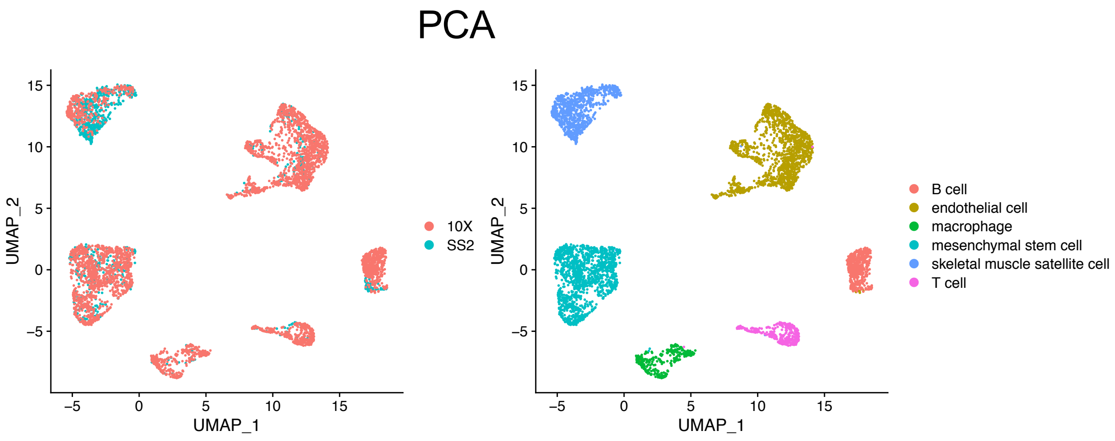

Supplementary Fig. 47 UMAP plots of the integrated scRNA-seq dataset for limb muscle from Tabula Muris using PCA as the dimension reduction method. The plots were colored by platform (left) and by cell type (right).

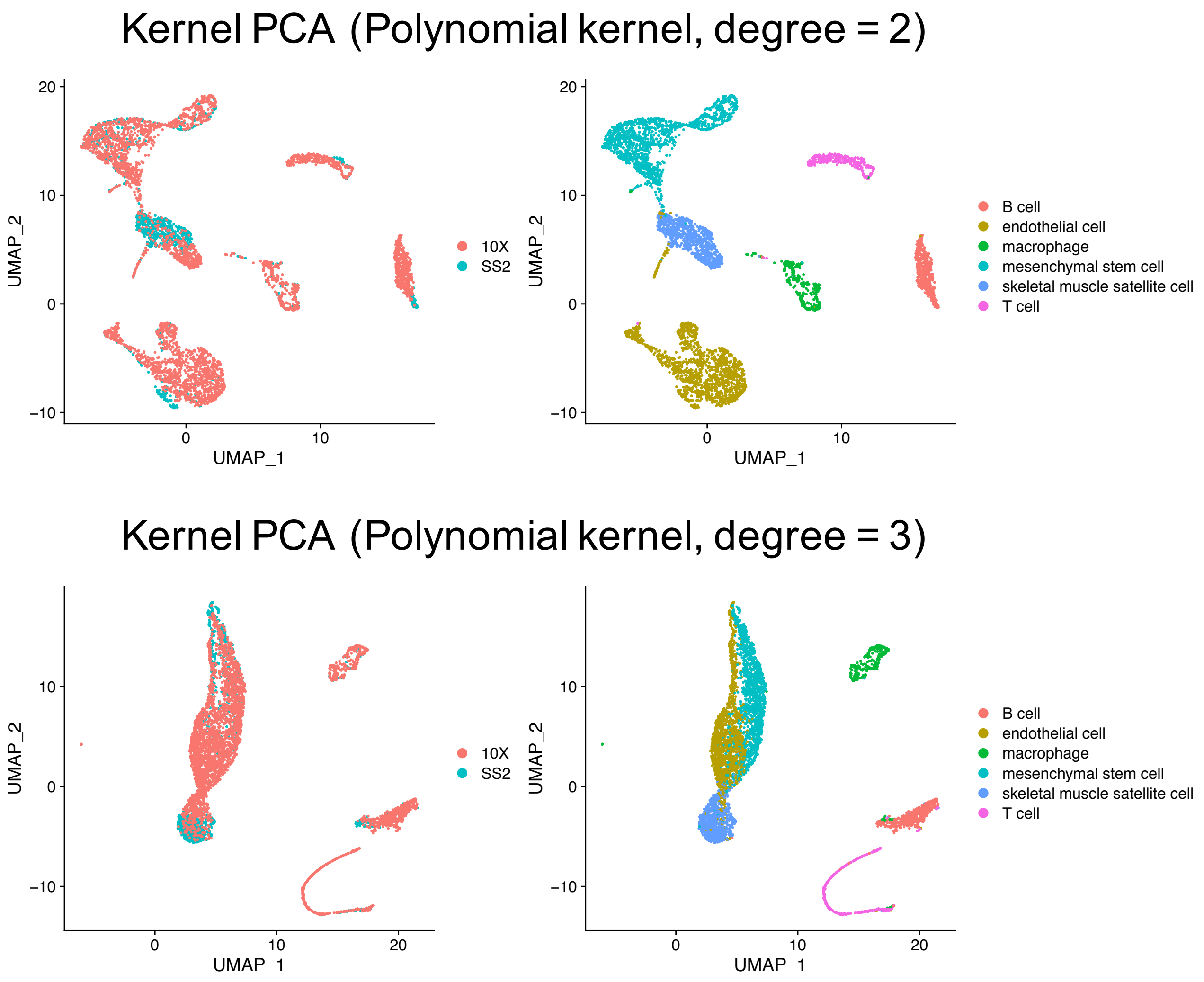

Supplementary Fig. 48 UMAP plots of the integrated scRNA-seq dataset for limb muscle from Tabula Muris using kernel PCA (the polynomial kernel function with different degrees) as the dimension reduction method. The plots were colored by platform (left) and by cell type (right).

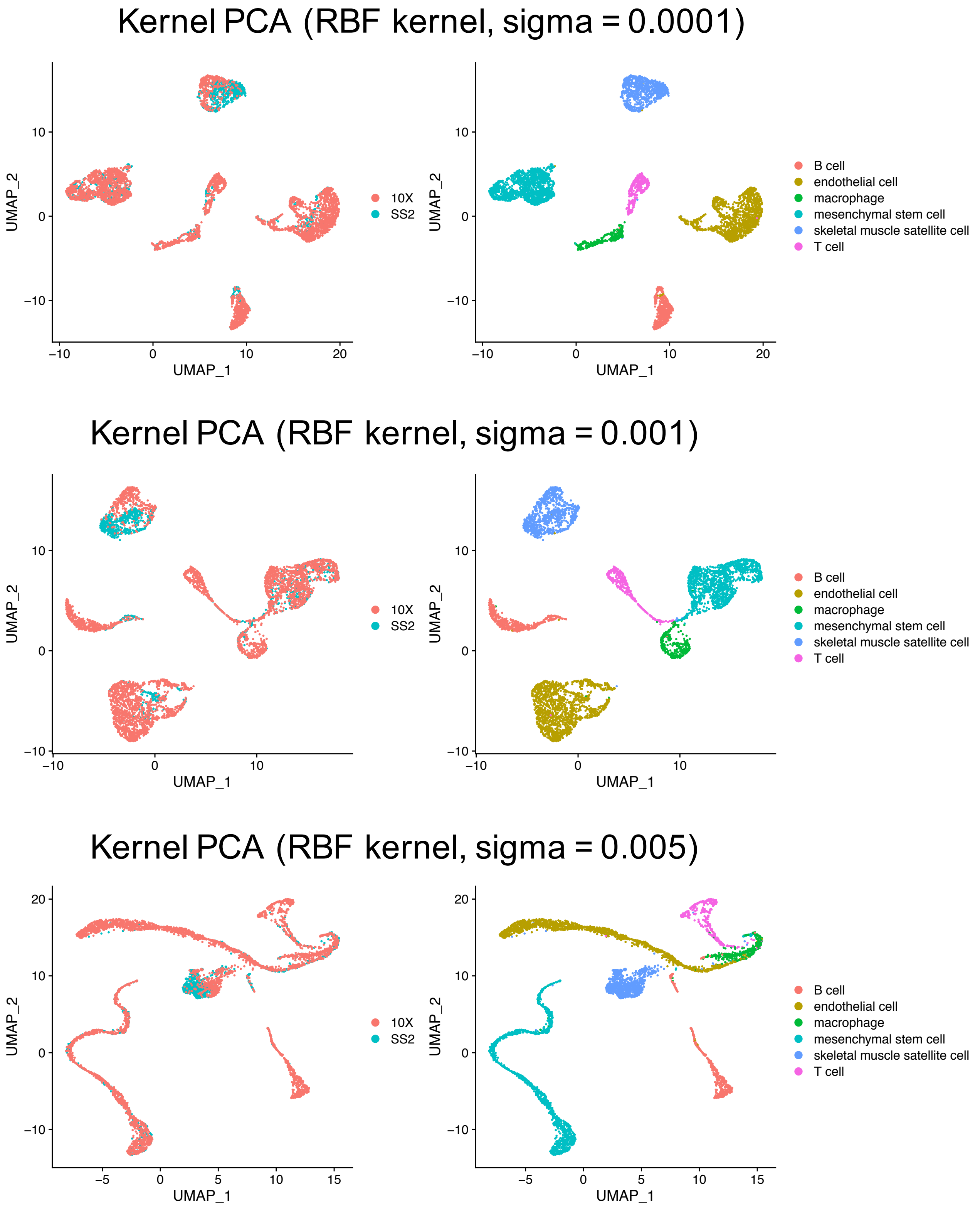

Supplementary Fig. 49 UMAP plots of the integrated scRNA-seq dataset for limb muscle from Tabula Muris using kernel PCA (the radial basis kernel function with different scaling parameters: sigmas) as the dimension reduction method. The plots were colored by platform (left) and by cell type (right).

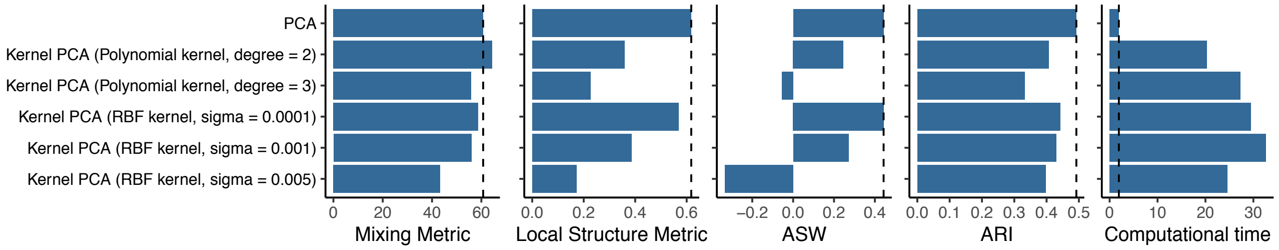

Supplementary Fig. 50 The metrics (mixing metric, local structure metric, ASW and ARI) for evaluating the integration performance and the computational time (in seconds) of the limb muscle data from Tabula Muris using different dimension reduction methods. The dashed lines were set at the values using PCA as reference lines.

References

1. Schaum N, Karkanias J, Neff NF, May AP, Quake SR, Wyss-Coray T, et al. Single-cell transcriptomics of 20 mouse organs creates a Tabula Muris. Nature. 2018;562(7727):367–72.
2. Stuart T, Butler A, Hoffman P, Hafemeister C, Papalexi E, Mauck WM, et al. Comprehensive Integration of Single-Cell Data. Cell. 2019;177(7):1888-1902.e21.
3. Consortium TTM, Ezran C, Liu S, Chang S, Ming J, Botvinnik O, et al. Tabula Microcebus: A transcriptomic cell atlas of mouse lemur, an emerging primate model organism. bioRxiv. 2021;2021.12.12.469460.
4. Travaglini KJ, Nabhan AN, Penland L, Sinha R, Gillich A, Sit R V., et al. A molecular cell atlas of the human lung from single-cell RNA sequencing. Nature. 2020;587(7835):619–25.
