# Supplementary material for: FIRM: Flexible integration of single-cell RNA-sequencing data for large-scale multi-tissue cell atlas datasets
Source: Brief Bioinform. 2022 May 14;23(5):bbac167. doi: 10.1093/bib/bbac167 (PMC9487597; doi:10.1093/bib/bbac167)
Supplement: 20220330_Supplementary-Table_bbac167 [file 20220330_supplementary-table_bbac167.pdf]

Supplementary Table. Problems of benchmarked methods for integration of SS2 and 10X datasets from Tabula Muris, Tabula Microcebus and Human Lung Cell Atlas.

Dataset			Figure	Method							
				scVI	ZINB-WaVE	BBKNN	BUSseq	LIGER	Harmony	MNN	Scanorama
Tabula Muris		Aorta	Supplementary Fig. 8	inadequate mixing	inadequate mixing				inappropriate mixing of the cardiac muscle cells and erythrocytes		
		Bladder	Supplementary Fig. 9	inadequate mixing	inadequate mixing	inadequate mixing	inadequate mixing				
		Limb muscle	Supplementary Fig. 10	inadequate mixing	inadequate mixing	inadequate mixing					
		Liver	Supplementary Fig. 11	inadequate mixing	inadequate mixing						
		Lung	Supplementary Fig. 12	inadequate mixing	inadequate mixing		inadequate mixing				
		Mammry gland	Figure 2	inadequate mixing	inadequate mixing	inadequate mixing low ARI		inappropriate mixing of the B cells, macrophages and T cells			
		Marrow	Supplementary Fig. 13	inadequate mixing	inadequate mixing		discretization of the granulocytes and granulocytopoietic cells				
		Spleen	Supplementary Fig. 14								inadequate mixing
		Trachea	Supplementary Fig. 15		inadequate mixing		discretization of the mesenchymal cells		discretization of the mesenchymal cells		
Tabula Microcebus	lemur 1	Blood	Supplementary Fig. 16	inadequate mixing			discretization of the neutrophils		discretization of the neutrophils	small clusters and low ARIs	
		Brain	Supplementary Fig. 17	inadequate mixing						small clusters and low ARIs	
		Lung	Supplementary Fig. 18	inadequate mixing					discretization of the neutrophils	small clusters and low ARIs	
	lemur 2	Bladder	Supplementary Fig. 19	inadequate mixing						small clusters and low ARIs	
		Blood	Supplementary Fig. 20	inadequate mixing		inadequate mixing	discretization of the neutrophils		inappropriate mixing of the capillary cells and neutrophils		
		Bone marrow	Supplementary Fig. 21	inadequate mixing	inadequate mixing				discretization of the neutrophils		
		Kidney	Supplementary Fig. 22	inadequate mixing	inadequate mixing						
		Lung	Supplementary Fig. 23	inadequate mixing	inadequate mixing		inadequate mixing				
		Spleen	Supplementary Fig. 24	inadequate mixing	inadequate mixing	inadequate mixing	inadequate mixing				inadequate mixing
	lemur 4	Bladder	Supplementary Fig. 25	inadequate mixing							
		Bone	Supplementary Fig. 26	inadequate mixing	inadequate mixing						
		Bone marrow	Supplementary Fig. 27				discretization of cells of the same type				
		Eye retina	Supplementary Fig. 28	inadequate mixing			inadequate mixing				
		Heart	Supplementary Fig. 29						small clusters		

Tabula Microcebus	lemur 4	Kidney	Supplementary Fig. 30	inadequate mixing	inadequate mixing			inappropriate mixing of the subtypes of natural killer cells and the T-cells			
		Liver	Supplementary Fig. 31	inadequate mixing					small clusters		
		Pancreas	Supplementary Fig. 32	inadequate mixing						small clusters and low ARIs	
		Small intestine	Supplementary Fig. 33	inadequate mixing	inadequate mixing			inappropriate mixing of the subtypes of natural killer cells and the T-cells			
		Spleen	Supplementary Fig. 34	inadequate mixing	inadequate mixing				inappropriate mixing of the eosinophils and B cells		inadequate mixing
		Testes	Supplementary Fig. 35	inadequate mixing	inadequate mixing		discretization of the pachytene spermatocytes, round spermatids, elongating spermatids, and elongated spermatids				
		Tongue	Supplementary Fig. 36	inadequate mixing				inappropriate mixing of the basal cells and the suprabasal cells			
Human Lung Cell Atlas			Supplementary Fig. 37	inadequate mixing	inadequate mixing	inadequate mixing	inadequate mixing	inappropriate mixing of the natural killer cells and the T-cells			
